# Synchronizing climate-carbon cycle heartbeats in the Phanerozoic vegetated icehouses

**DOI:** 10.1038/s41467-025-64238-9

**Published:** 2025-10-16

**Authors:** Qiang Fang, Huaichun Wu, Isabel P. Montañez, Shu-zhong Shen, Christian Zeeden, Xiangdong Wang, Shihong Zhang, David De Vleeschouwer

**Affiliations:** 1 State Key Laboratory of Geomicrobiology and Environmental Changes, China University of Geosciences, Beijing, China; 2 Frontiers Science Center for Deep-time Digital Earth, China University of Geosciences (Beijing), Beijing, China; 3 Key Laboratory of Polar Geology and Marine Mineral Resources (China University of Geosciences (Beijing)), Ministry of Education, Beijing, China; 4 Department of Earth and Planetary Sciences, University of California, Davis, One Shields Drive, Davis, CA USA; 5 State Key Laboratory of Critical Earth Material Cycling and Mineral Deposits, School of Earth Sciences and Engineering and Frontiers Science Center for Critical Earth Material Cycling, Nanjing University, Nanjing, China; 6 LIAG - Institute for Applied Geophysics, Hannover, Germany; 7 Institute of Geology and Palaeontology, University of Muenster, Münster, Germany

**Keywords:** Palaeoclimate, Palaeoceanography

## Abstract

Earth experienced state-specific climate-carbon cycle feedbacks during the Late Cenozoic Ice Age (LCIA). Whether similar feedbacks existed in the penultimate icehouse, the Late Paleozoic Ice Age (LPIA), remains uncertain. Here, we present phase relationships between eccentricity-paced climate cycles and carbonate carbon isotope across ~337–300 Ma. Up to 307 Ma, low-latitude continental carbon reservoirs expanded during eccentricity-forced coolings, resembling the Oligocene and Miocene climate-carbon cycle dynamics. After 307 Ma, this relationship reversed, analogous to the Plio-Pleistocene dynamics. We attribute this reversal to the increasing importance of high-latitude biome dynamics, comparable to what occurred at 6 Ma in the LCIA. Paralleling LPIA (335–301 Ma) and LCIA (past 34 Myr) records using this event reveals quasi-synchronization in the interaction of astronomical forcing, carbon cycling and glacial events from onset to apex of two icehouses. We propose that, despite different boundary conditions, extraterrestrial forcing shaped the evolutionary trajectory of Phanerozoic vegetated icehouses.

## Abstract (web summary)

Development of the Late Cenozoic Ice Age and Late Paleozoic Ice Age follows a comparable climate trajectory, involving secular trends superimposed with multiple astronomically forced climate-carbon cycles and transient climatic events.

## Introduction

The Cenozoic Era underwent a significant transition from a greenhouse to an icehouse state beginning ~34 million years ago (Ma)^1^, after which global climate followed a long-term cooling trend, leading to our current icehouse with bipolar ice sheets^2,3^. On an astronomical (Milanković) timescale (20–400 kyr), the Late Cenozoic Ice Age (LCIA) exhibited state-dependent patterns of climate-carbon cycle interactions^4,5^. Between 34 and 6 Ma, low-latitude continental carbon reservoirs expanded mostly during the astronomically-forced cool intervals, which changed at 6 Ma^5^, along with a decrease in atmospheric CO_2_ concentrations during late Miocene cooling^6^. Conversely, after 6 Ma to the present, global carbon cycle dynamics were likely influenced by the spatial redistribution of high-latitude biomes on orbital timescales due to their increasingly important dynamics^5^. As the tundra ecosystems have much smaller carbon storage capacities than the boreal forests^7^, the continental carbon reservoirs shrunk in the colder periods on eccentricity time scales^5^.

**Fig. 1:**
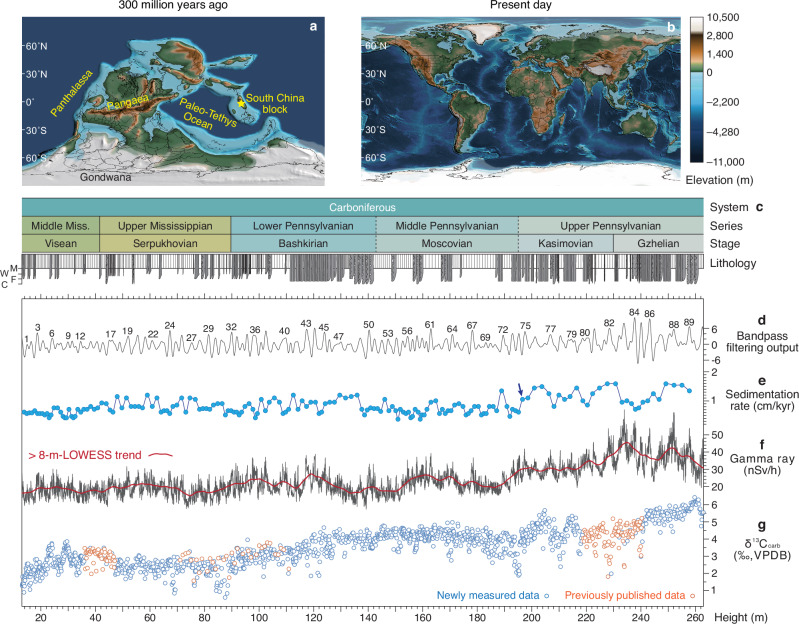
Geological background and datasets. **a** Late Carboniferous paleogeographic map^9^. **b** Modern topography and bathymetry map^9^. **c** Lithostratigraphy and chronostratigraphy of the Narao section. The basal Serpukhovian, Bashkirian and Gzhelian are determined at 41.8 m^24^, 89.9 m^25^ and 229.61 m^26^, respectively. M—lime mudstone; W—wackestone; F—fine-grained packstone; C—coarse-grained packstone; Miss—Mississippian. **d** Filtering output of the interpreted long eccentricity cycles. The Gaussian filter with a passband of 0.43 ± 0.28 cycles/m was used on raw gamma ray (GR) data. **e** Calculated sedimentation rate from 405-kyr astronomical calibration. An arrow points a sudden increase in the sedimentation rate. **f** GR series and its > 8-m LOWESS trend. **g** Carbonate δ^13^C (δ^13^C_carb_) series. Published data are after refs. ^24,25,67^.

Earth’s penultimate icehouse, the Late Paleozoic Ice Age (LPIA, ~340–280 Ma), is viewed as a deep-time analog for the current glacial world^8^. Despite very different boundary conditions determined by continental configurations (Fig. 1a, b) and oceanic circulation patterns^9^, the potential for parallels is suggested by similarities between the two icehouse periods. First, the LPIA archives a dynamic glacial evolution under low atmospheric CO_2_ concentrations comparable to those of the Pleistocene glacial cycles to present-day^10^, and secondly the paleo-continents were also covered by complex vegetation ecosystems^11^. Rich palaeobotanical evidence from the Pangaean tropics reveals repeated shifts in vegetation from glacial wetland forests (e.g., arborescent lycopsids and medullosan dominated) to interglacial seasonally-dry flora (e.g., cordaitalean, conifer, and marattialean tree fern dominated) throughout the late Carboniferous^12^, in response to eccentricity-paced variations in solar insolation and *p*CO_2_^10,13,14^. This was a finding consistent with simulations made using the Earth System^15^ and ecologic models^16^. Net positive terrestrial carbon sequestration occurred during late Carboniferous glacials driven by the predominance of wetland coal forests^10^. At 307 Ma, arborescent lycopsids abruptly disappeared from Euramerican biomes^17,18^, coincident with the onset of long-term tropical aridification^19^, remaining only in Cathaysia (China)^20,21^ that held everwet condition through the late Paleozoic^19^. The continental-scale aridification likely intensified the repeated vegetational turnovers that characterized the remainder of the LPIA^12^. Thus, it is possible that the widespread loss of tropical coal forests may have led to a change in the response of the global carbon cycle to astronomical forcing. This hypothesis, however, remains untested.

Here, we report a high-temporal resolution ( ~ 25 kyr) δ^13^C time series developed using ~1500 bulk carbonate samples collected from a 250-m deep-marine succession (Narao section) in South China (Fig. 1a and Supplementary Fig. 1), which we calibrated to an astronomical time scale constructed by using the cyclostratigraphic analysis on 5000 gamma ray (GR) data (Fig. 1f; see “Methods” section). This new δ^13^C_carb_, integrated with a GR time series, spans the interval from the onset ( ~ 335–330 Ma^8^) to the apex (300 ± 5 Ma^22^; referred to as 300 ± 1 Ma^23^ herein) of the LPIA, providing new insight into the response of global carbon cycling to astronomical forcing during the penultimate icehouse.

## Results and discussion

### Cyclostratigraphic analysis

**Fig. 2:**
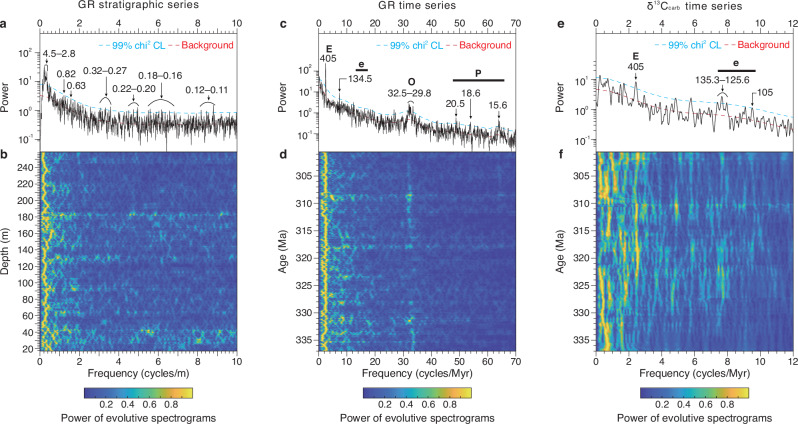
Cyclostratigraphic analysis of the datasets from the Narao section. **a** Smoothed window averaging (SWA) background fitting for the power spectrum of gamma ray (GR) stratigraphic series, including the standard Chi-squared (Chi^2^) 99% confidence level (CL). **b** Evolutionary spectrogram of GR series (8-m-analysis window and 0.1-m steps). **c** SWA background fitting for the power spectrum of 405-kyr-calibrated GR series. **d** Evolutionary spectrogram of 405-kyr-calibrated GR series (1-Myr-analysis window width and 0.05-Myr steps). **e** SWA background fitting for the power spectrum of 405-kyr-calibrated carbonate δ^13^C (δ^13^C_carb_) series. **f** Evolutionary spectrogram of 405-kyr-calibrated δ^13^C_carb_ series (6-Myr-analysis window width and 0.2-Myr steps).

The Viséan to Gzhelian sediments of the Narao section were deposited on an open marine deep-water (carbonate) slope^24,25^ and are characterized by an average sedimentation rate of ~0.7 cm/kyr. This estimate is derived from dividing the 187.81 m of stratigraphy between the basal Serpukhovian (41.8 m^24^) and basal Gzhelian (229.61 m^26^) (Fig. 1c), by a total duration of ~26.7 Myr^27^. Increased terrestrial input during sea-level falls would have provided more clay to the studied region^28^, resulting in higher GR values. Time series analysis of the new GR data reveals significant wavelengths of 4.5–2.8 m, 0.82 m, 0.63 m, 0.32–0.27 m, 0.22–0.20 m, 0.18–0.16 m, and 0.12–0.11 m, above a 99% Chi-squared confidence level (Fig. 2a; see “Methods” section), after removing a > 8 m trend (red line in Fig. 1f). These cycles can be clearly observed in the raw data series and data after applying different ways of detrending (Supplementary Fig. 2). Given the estimated sedimentation rate, the 4.5–2.8 m cycle most likely corresponds to the 405-kyr eccentricity cycle. Although the stability of the 405-kyr eccentricity cycle in deep time remains a topic of ongoing debate^29,30^, geochronological evidence supports its relatively stable period during much of the Carboniferous^31–33^, which is further substantiated by results derived from 64 solutions in the ZB23 astronomical solution^34^ that currently are available (Supplementary Fig. 3).

**Fig. 3:**
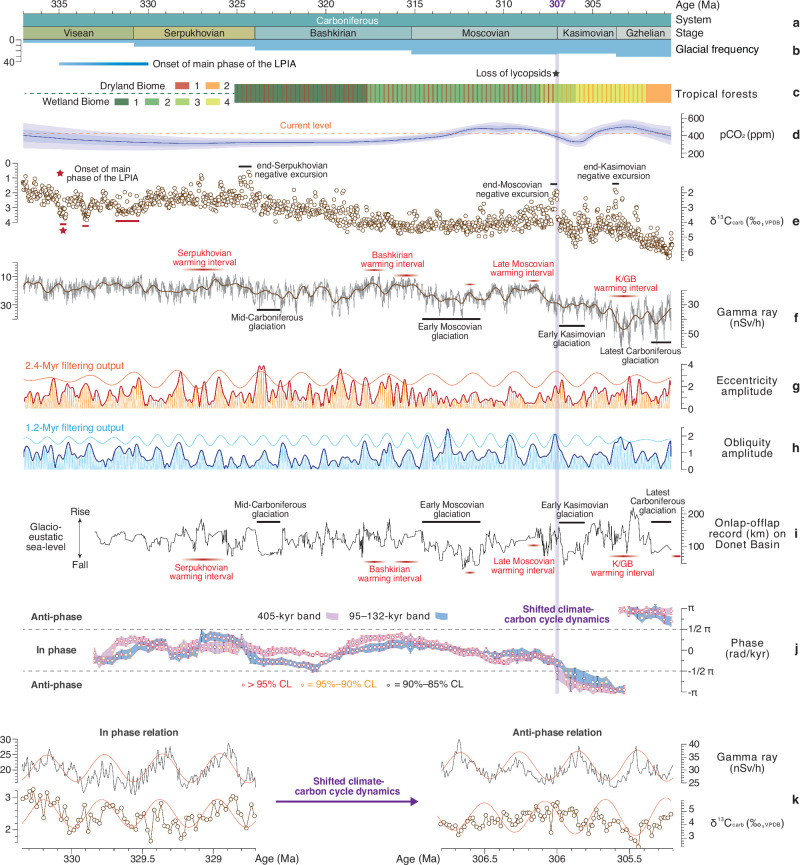
Astronomical forcing and geological records between 337–300.6 Ma. **a** Our refined Carboniferous chronostratigraphy with numerical ages for the basal Serpukhovian and Bashkirian, and ages for the basal Moscovian, Kasimovian and Gzhelian are after Global Time Scale (GTS) 2020^27^. **b** Glacial frequency^74^. LPIA—Late Paleozoic Ice Age; **c** Tropical forests including dryland biomes (DB) and wetland biomes (WB)^14^. WB 1—lycopsid-dominated forest; WB 2—lycopsids/cordaitalean co-dominated forest; WB 3—lycopsid-dominated forest, with an understory of marattialean tree ferns; WB 4—marattialean tree fern-dominated forest; DB 1—canopy cordaitalean-dominated forest; DB 2—walchian conifer-dominated open woodland. **d** Compiled atmospheric CO_2_ record^8^. **e** Astronomically calibrated carbonate δ^13^C (δ^13^C_carb_) series from Narao section. **f** Astronomically-calibrated gamma ray (GR) series from Narao section and its low-pass filter output (1.5 Myr^−1^). KGB—Kasimovian/Gzhelian boundary. **g** Amplitude modulation (AM) of orbital eccentricity obtained from our GR series. AM (orange bold curve) of the ~100-kyr short eccentricity band (9 ± 2 Myr^−1^) and its 2.4-Myr filter output (0.42 ± 0.08 Myr^−1^). **h** AM of obliquity obtained from our GR series. AM (blue bold curve) of the ~31-kyr obliquity band (32.7 ± 1.8 Myr^−1^) and its 1.2-Myr filtering output (0.85 ± 0.12 Myr^−1^). **i** Onlap (transgression)–offlap (regression) history from the Donets Basin^33^, adjusted to the GTS2020^27^. Glaciation events are indicated by the glacio-eustatic sea-level lowstands^33^. Warming intervals from refs. ^8,38^. **j** Evolution of phase-relationship between GR and δ^13^C_carb_ on 405-kyr (purple area) and 95–132-kyr (blue area) eccentricity time scales (4-Myr-analysis window width and 0.25-Myr steps). CL—confidence level. **k** Two intervals (i.e., 330.35–328.7 Ma and 306.8–305.2 Ma) demonstrating the inphase and anti-phase behaviors of two proxies on 405-kyr band. 405-kyr eccentricity signals (red curves) were obtained using a passband of 2.46 ± 0.15 Myr^−1^ on each data series.

Ninety 4.5–2.8 m cycles were recognized through bandpass filtering of the GR time series (Fig. 1d). Calibrating these cycles to the 405-kyr eccentricity period reveals dominant spectral peaks at 405 kyr (long eccentricity), 134.5 kyr (short eccentricity), 32.5–29.8 kyr (obliquity), and 20.5 kyr, 18.6 kyr and 15.6 kyr (precession; Fig. 2c, d), corresponding to average periods of the Carboniferous Milanković cycles (i.e., ~405 kyr, ~132 kyr, ~31.5 kyr, ~20.2 kyr, ~19.3 kyr and ~16.7 kyr)^34^ (Supplementary Fig. 3). The calculated change in sedimentation rates based on astronomical calibration (Fig. 1e) tracks with those obtained using an objective statistical methodology, evolutionary time scale optimization^35^ (Supplementary Fig. 4; see “Methods” section), providing confidence in our astronomical calibration. The Kasimovian/Gzhelian boundary at Narao succession was assigned a numerical age of 303.7 ± 0.10 Ma^27^, and an uncertainty of ±0.20 Myr (half a 405 kyr cycle) was given for the astronomical calibration. This yields a floating astronomical time scale (i.e., ~337–300 Ma) for the interval spanning the stepwise onset to peak of the LPIA, with a combined uncertainty of ±0.3 Myr (Fig. 3a; Supplementary Text 1). An abrupt increase in the sedimentation rate (dark blue arrow in Fig. 1e), accompanied by the enhanced terrestrial inputs (Fig. 1f), is observed at a stratigraphic depth of ~196 m, which has been astronomically calibrated to an age of ~306.9 Ma. We suggest that it may represent a regional response to glacio-eustatic sea-level lowering during the early Kasimovian^33^, occurring within the context of secular cooling towards the Carboniferous–Permian transition^22,23^.

Astronomical cycles of million-year-scale can be determined by the amplitude modulation assessment using Hilbert transforms^36^ (see “Methods” section). Amplitude modulation analysis was conducted on the bandpass-filtered ~32-kyr obliquity (Fig. 3h) and ~100-kyr short eccentricity signal (Fig. 3g). Results show main periods of ~1.17 Myr and ~2.36 Myr for the long-period obliquity and eccentricity amplitude modulation cycles, respectively (Supplementary Fig. 5c, d). The periods of these million-year-scale astronomical cycles during ~337–300 Ma are indistinguishable from those in the astronomical solution (e.g., ZB18a) for the past 36 Myr^37^ (Supplementary Fig. 6).

### Astrochronologic constraint on the Carboniferous climate-carbon cycle history

The astronomically calibrated GR time series for the Narao succession provides a high-resolution relative sea-level history (Fig. 3f), that aligns on the million-year time scale with inferred sea-level reconstructed for the late Viséan ( ~ 333 Ma) through the latest Carboniferous ( ~ 300 Ma) from other low-latitude records (i.e., Donets Basin, Ukraine^33^ and North America^38^; Fig. 3i) (Supplementary Text 2). On astronomical time scales, the onlap (transgression)–offlap (regression) record from the Donets Basin and the GR time series from the Narao section display a constant anti-phase relationship within the 1.17 Myr (–160° ± 18°) and 400-kyr (–173° ± 23°) bands (Supplementary Fig. 7a), indicating the potential synchronicity of the astronomically-paced eustasy at these low latitude locations. On the ~1.2 Myr band, the long-period obliquity amplitude modulation cycle shows a nearly consistent anti-phase relation with the Narao GR time series (–135° ± 18°; Supplementary Fig. 7b), and an in-phase relationship with the onlap–offlap record (40° ± 11°; Supplementary Fig. 7c). These phase relationships support a linkage between sea-level falls and obliquity nodes (minima of long-period obliquity amplitude modulation cycles) during the Carboniferous, this is congruent with findings for the LCIA^39^.

The astronomically calibrated marine δ^13^C_carb_ time series from the Narao section is globally correlated, and it provides high temporal resolution insight into the nature of carbon cycle perturbations. Three positive δ^13^C_carb_ excursions during the late Visean–earliest Serpukhovian interval (red bars in Fig. 3e) overlap with the proposed timings of the stepwise onset of the LPIA^8^ (Fig. 3b). Astrochronologic constraints for England shallow marine carbonates from the U.K. indicate a basal late Asbian age of 334.48 ± 0.35 Ma^40^. Thus, the δ^13^C_carb_ positive excursion at ~335–334.6 Ma is likely time-equivalent with a cooling pulse inferred from an earliest Asbian positive shift in biogenic apatite δ^18^O values and ice buildup^41^, suggesting a possible mechanistic link between the onset of the LPIA and the enhanced carbon burial. Three subsequent and prominent negative δ^13^C_carb_ excursions (black bars in Fig. 3e) at the end-Serpukhovian (excursion of ~ –1.2‰; ~324.9–324.4 Ma), the end-Moscovian ( ~ –2‰; ~307.3–307.0 Ma) and the end-Kasimovian ( ~ –2‰; ~303.8–303.7 Ma) were observed in the Narao record, which can be correlated to the excursions archived in western paleotropical Pangaea successions^42,43^ (Supplementary Fig. 8). Collectively, our new record provides an astronomically-calibrated, high-resolution stratigraphic reference for the evolution of low-latitude glacio-eustasy and changing carbon cycle dynamics for the time interval from ~337–300 Ma.

### Eccentricity-scale climate-carbon cycle

A time-evolutive phase analysis between the astronomically-calibrated GR and δ^13^C_carb_ time-series was used to differentiate in-phase and anti-phase behavior in the 405-kyr and 100-kyr eccentricity bands (Fig. 3j). For the interval of 337 to 307 Ma and on the 405-kyr eccentricity time scale, GR and δ^13^C_carb_ exhibit oscillations around an in-phase relationship, indicating that maxima in δ^13^C_carb_ are associated with eccentricity-paced glacial-eustatic sea-level falls. The in-phase behaviour inverts to anti-phase at ~307 Ma (purple shading in Fig. 3j), along with an abrupt increase in sedimentation rate and GR values (Fig. 1e, f). It suggests a major shift in the response of the continental carbon sink(s) to the glacial advances switched from expansion to contraction. This shifted phase relation occurred almost simultaneously in the short eccentricity band (blue shading in Fig. 3j and Supplementary Fig. 9). A similar shift in phasing is observed in another carbonate slope succession in South China (Naqing section; Supplementary Fig. 10). The shifted phase relation can also be observed via the stationary phase relation analysis between δ^13^C_carb_ from Narao and onlap–offlap record from the Donets Basin (Supplementary Fig. 7f, g), likely reflecting a global signal for the reorganization of climate-carbon cycle system on eccentricity time scale.

Based on these observed phase relationships and robust paleobotanical evidence^12,14^, we propose the climate-carbon cycle dynamics during the period of 335–307 Ma. The dynamics were mechanistically linked by the expansion of wetland coal forests and increased carbon sequestration and storage of isotopically light carbon (^12^C) through enhanced peat burial^12^ during eccentricity minima. Earth system^44^ and coupled climate and ecosystem modeling^45,46^ indicate that the cooler and wetter conditions of Carboniferous eccentricity minima promoted the widespread expansion of wetland forests. In turn, increased burial of ^12^C-enriched organic matter on land during eccentricity-forced glacial periods would have contributed to an increase in the δ^13^C of marine dissolved inorganic carbon, as is observed in the increases in the δ^13^C_carb_ at maxima of the eccentricity-paced GR cycle.

In contrast, we propose that for the subsequent interval between ~307–300 Ma, eccentricity minima led to cold and dry climate conditions, resulting in a shrinkage of the ^12^C-enriched continental carbon reservoirs at high latitudes. This shift in carbon cycle dynamics coincides with a global cooling ( ~ 307–304.5 Ma), characterized by ~50% drop in *p*CO_2_^10,47^ (Fig. 3d), a short-lived but intense glaciation^33^ (Fig. 3f, i) (Fig. 3c). The subsequent increase of two to three times in *p*CO_2_ during the Kasimovian–Gzhelian transition (Fig. 3d) appears to be insufficient to reverse this dynamic (Fig. 3j). We speculate that the center of terrestrial carbon burial may have shifted from the low to high latitudes in response to the onset of long-term tropical aridification at the close of the Middle Pennsylvanian. Key evidence supporting this hypothesis includes precipitous loss of coal forests and peat burial by the end of the Middle Pennsylvanian^14,48^, which served as the primary source of terrestrial carbon sequestration prior to 307 Ma^20^. Furthermore, ecosystem modeling of late Carboniferous terrestrial ecosystems indicates major changes in high-latitude forest cover during glacial and interglacial extremes in particular the higher tolerance of drought-adapted vascular plants to freezing conditions^16^, highlighting the potential for the increasing importance of different high-latitude carbon reservoirs, e.g., tundra and boreal forests, in response to climate deterioration and amelioration. We suggest that global carbon cycle dynamics could have been substantially influenced by such spatial redistribution in high-latitude biomes. Glacial advances during attenuated eccentricity cycles would have caused an upsurge in tundra areal extent at high latitudes, accounting for the anti-phase relationship observed between δ^13^C and GR after ~307 Ma.

Although we tend to support the biome areal competition hypothesis, other studies have stressed the relevance of organic carbon burial in the ocean during the LPIA^49–51^. Iron-rich dust flux likely increased substantially during late Carboniferous glacials, which may have stimulated marine productivity that would have enhanced marine carbon burial^49,51^. This hypothesis was proposed to explain the secular decoupling of continental weathering and *p*CO_2_ after ~303 Ma^50^. Despite more vigorous ocean circulation at glacial phases during late Carboniferous^52^, it remains uncertain as to which boundary conditions would have driven reversals in the response of organic carbon burial in the ocean to astronomical forcing. Given the great capacity of the terrestrial biosphere to influence the LPIA carbon cycling^10^, we view the areal competition between low- and high-latitude biomes as a more plausible explanation for the observed shift in phase relationships of the eccentricity-scale climate-carbon cycling at ~307 Ma. Future investigations will be necessary to determine whether climate-carbon cycle feedbacks shifted along major deglaciation events and vegetation turnovers during the early Permian period^53^.

### Climate- and carbon-cycle evolution in icehouse conditions

**Fig. 4:**
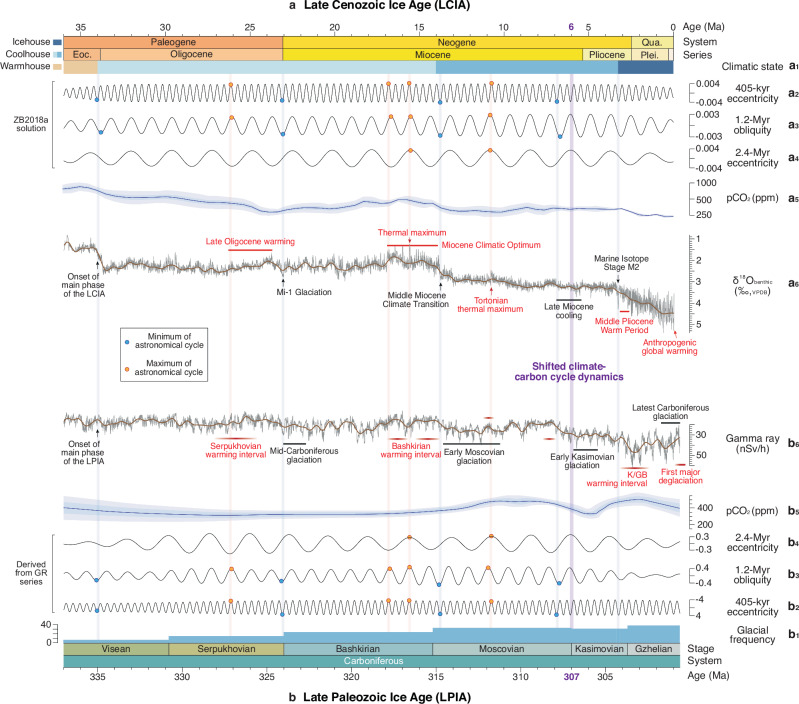
Records showing the climatic heartbeats of the Late Cenozoic Ice Age (LCIA) and Late Paleozoic Ice Age (LPIA). Red (blue) shadings indicate the alignment of the maxima (minima) of the orbital cycles. **a** Records of the LCIA. **a**_**1**_ Climate states^3^. **a**_**2**_**–a**_**4**_ 405-kyr eccentricity, 1.2-Myr obliquity and 2.4-Myr eccentricity cycles extracted from the ZB18a astronomical solution (details shown in Supplementary Fig. 6). **a**_**5**_ Atmospheric CO_2_ estimates^6^. **a**_**6**_ Cenozoic global reference benthic foraminifer δ^18^O (δ^18^O_benthic_) data^3^. Climatic events^2,3^. **b** Records of the LPIA. **b**_**1**_ Glacial frequency^74^. **b**_**2**_**–b**_**4**_ 405-kyr eccentricity, 1.2-Myr obliquity and 2.4-Myr eccentricity cycles extracted from the calibrated GR series from Narao (details shown in Fig. 3). **b**_**5**_ Atmospheric CO_2_ estimates^8^. **b**_**6**_ 405-kyr-calibrated GR series. Climate events are shown in Fig. 3i.

The shift in the carbon-climate cycle dynamics was linked to the growing significance of competing effects between high-latitude biomes during the LCIA^5^. This shift occurred at 6 Ma^5^, coinciding with maxima in ~1.2-Myr and ~2.4-Myr cycles (Fig. 4a_2–4_) under a declining atmospheric *p*CO_2_ concentration^6^ (Fig. 4a_5_). A comparable scenario is revealed at ~307 Ma for the LPIA (Fig. 4b_2–5_). Thus, this event occurred in high-amplitude insolation variations superimposed on secular cooling trend in both icehouses. This similarity indicates that the increased importance of high-latitude vegetation dynamics may be a critical threshold for the secular evolution of the vegetated icehouses. Since the late Paleozoic Era, continental areas in the Northern Hemisphere have consistently increased in extent relative to those in the Southern Hemisphere^9^. Specifically, the Pangaean supercontinent had large areas of southern high-latitude land and the Central Pangaean Mountains extended across the tropical continent (Fig. 1a), which is substantially different with the late Cenozoic paleogeography^9^ (Fig. 1b). The discovery in this study of analogous thresholds, despite the different paleogeographic background, enhances confidence in a more comprehensive comparison of the evolutionary patterns between the two icehouses, e.g., climate rhythmicity and transient events.

Our study documents the climate- and carbon-cycle evolution of the two icehouses follows similar rhythms from their onset to apex (Fig. 4). Astronomically tuned deep-sea benthic foraminifer δ^18^O and δ^13^C records are utilized to track glacial variation (Fig. 4a_6_) and the global carbon cycle (Supplementary Fig. 11) during the LCIA^3^. Paralleling the δ^18^O data (36–0 Ma) and GR series (337–301 Ma) shows a nearly in-phase relation on the pronounced “heartbeats” at ~405-kyr, ~1.2-Myr and ~2.4-Myr periods (Supplementary Fig. 7h), pacing with the multiple astronomical cycles (Fig. 4). The δ^13^C time series for the two icehouses show an in-phase relation for 405-kyr eccentricity cycles (Supplementary Fig. 7i), likely revealing the quasi-synchronicity of the climate-carbon cycle. It strengthens our hypothesis that astronomical forcing mediated the climate-carbon cycle dynamics on kilo-year time scale in the two icehouse periods. No stable relationship is observed between the δ^13^C time series of the two icehouses on million-year time scales, probably indicating a more complex carbon cycle dynamic on longer time scales.

We also observe that the timing of certain transient climatic events in the Carboniferous and late Cenozoic eras was approximately comparable, following the transition of Earth’s climate system into an icehouse state (Fig. 4a_6_, b_6_). Since the onset of the LCIA at ~34 Ma during the Eocene–Oligocene climatic transition, global climate was characterized by stepwise cooling occurring around ~23 Ma (Mi-1 Glaciation at Oligocene/Miocene boundary), ~14 Ma (onset of the middle Miocene climate transition), ~7 Ma (initiation of late Miocene cooling) and ~3.3 Ma (Marine Isotope Stage M2 in the late Pliocene, indicating the onset of the Pleistocene Ice Ages)^2,3^. These cooling trends were punctuated by distinct warming intervals or events, such as the late Oligocene warming ( ~ 26.3–23.7 Ma), the Miocene Climatic Optimum ( ~ 17–14 Ma) and the Middle Pliocene Warm Period ( ~ 3.3–3.0 Ma)^2,3^ (Fig. 4a_6_). The climate evolution of the LPIA (Fig. 4b_6_) exhibits a similar pattern to that observed in the LCIA. Stepwise expansion of continental ice sheets during the LPIA was associated with notable cooling events in the late Visean ( ~ 335 Ma), mid-Carboniferous ( ~ 324 Ma), early Moscovian ( ~ 315 Ma), early Kasimovian and latest Carboniferous ( ~ 302 Ma) glaciations. Overall cooling was interrupted by the warming intervals in Serpukhovian, Bashkirian and Kasimovian–Gzhelian transition^8,33,38^ (Fig. 4b_6_).

With a comprehensive understanding of the Cenozoic climate, there is a tendency to attribute the Cenozoic cooling/warming events to multiple contributing factors. Revisiting the Eocene–Oligocene climatic transition as an example, its primary drivers include the tectonic-oceanic hypothesis and the CO_2_ hypothesis. The first hypothesis posits that the enhanced thermal isolation of Antarctica, due to the widening of oceanic passages (such as the Drake and Tasman seaways), facilitated the expansion of Antarctic glaciation^54^. The second suggests that the dominating factor in the latest Eocene ice expansion is the decline in atmospheric CO_2_ levels down to a specific threshold^55^. However, the predominant forcing of climate changes remains a subject of debate, due to the intricate feedback mechanisms among the ocean, atmosphere, and Earth’s surface. Classically, climate regulation on Earth is predominantly associated with atmospheric *p*CO_2_ levels, which are influenced by the variations in carbon sources (e.g., volcanism) and sinks (e.g., continental weathering, organic carbon burial on land or in the ocean)^56^. There is no clear consensus regarding the factors responsible for long-term climatic shifts; debates often focus on whether carbon sources or sinks have been more influential. For instance, studies have connected both carbon sources or sinks to major climate changes of the LCIA^57–59^, and this is also the case for the LPIA^47,53,60^. Both factors were largely controlled by plate tectonics, resulting in gradual changes that predominantly occurred unidirectionally over multi-million-year timescales.

Beyond atmospheric CO_2_ levels, extraterrestrial factors may play a significant role in maintaining quasi-synchronization of these transient climatic events between two icehouses. We propose that tectonically controlled CO_2_ variations brought the climate to threshold conditions, and then orbital forcing may have caused these transient climatic events (thereby determining their timing), by pushing the system beyond the critical thresholds. The shifts in other boundary conditions—such as paleogeography and topography, oceanic gateway locations and bathymetry—cannot fully account for the climatic quasi-synchronization due to their independent changes within two icehouses. Efforts have been made to link astronomical forcing and cryospheric responses in the LCIA^2,61–63^, which would shed light on the climatic counterparts or analogs that occurred in the LPIA. The rapid cooling and glacial building may be associated with periods of low eccentricity and low-amplitude change in obliquity (see blue vertical lines in Figs. 4a_2–3_, 4b_2–3_), permitting the accumulation of summer snow becoming glaciation. Such orbital configurations may be one of the important forcings to tip the balance towards glaciation during intervals of declining CO_2_ levels below the threshold, initiating the glaciation (see the glaciation/cooling events in Figs. 4a_6_, 4b_6_). On the contrary, the secular cooling trends would have been interrupted by high eccentricity and 1.2-Myr obliquity-forced deglaciations (see red vertical lines in Figs. 4a_2–3_, 4b_2–3_). During eccentricity maxima, highly seasonal precipitation would have led to prolonged dry seasons, when the terrestrial organic carbon may have undergone oxidization due to seasonally drier climates^64^. Seasonal extremes would have been further amplified during orbital configurations of maxima in all 405-kyr eccentricity, 1.2-Myr obliquity, and 2.4-Myr eccentricity cycles, likely acting as positive feedbacks for the transient warming events, e.g., a thermal maximum at ~15.6 Ma during the Middle Miocene climatic optimum^65^ and the Tortonian thermal maximum at ~10.8 Ma^66^ (Fig. 4a_7_) in the LCIA, and short-term warming in middle Moscovian ( ~ 311.8 Ma^33^) in the LPIA.

### Implications for the deep-time climate change in icehouses

Our proxy-based study demonstrates that the development of the main phases of the LCIA and LPIA follows a comparable climate trajectory, even though significantly different boundary conditions exist in two icehouses. This trajectory involves secular trends superimposed with multiple astronomically forced climate-carbon cycles, transient climatic events and certain critical threshold. The LPIA should be regarded as a climate analog for the LCIA. Trends, rhythms, and aberrations in climate changes in one icehouse may provide critical information for their counterpart, thereby elucidating the dynamics, such as forcings, feedbacks, responses and climate thresholds, underlying the climate-carbon cycle linkages. The magnitude of environmental perturbations during the LPIA may have been amplified due to the super-continentality at the time, providing important insight into the functioning of physical, geochemical, and biological processes under a more dynamic glacial/interglacial variation. Moreover, the demise of the LPIA and turnover to greenhouse condition holds critical information regarding processes driving ice sheet destabilization, large-scale hydrological changes, and key feedback mechanisms influencing biological responses.

## Method

### Sampling and proxy data measurements

GR intensity was measured directly on the succession using an RS-230 Super Spec portable spectrometer, with a 150-s recording time for one data. A total of 5,000 data points were taken from the Narao section, measuring in total 250 m (13–263 m) with a sample distance of 0.05 m. Rock samples were consistently collected at ~10-cm intervals along the horizons where GR measurements were conducted. No lithological bias occurred during the field sampling process. Based on the established astronomical timescale, the temporal resolution of the δ^13^C_carb_ measurements is ~25-kyr. The Narao δ^13^C_carb_ record was generated using samples of micritic limestone matrix drilled from fresh rock surfaces. Carbonate powders were reacted with supersaturated 100% phosphoric acid at 70 °C using a Kiel IV Carbonate Device coupled with a Finnigan 253 plus mass spectrometer. The maximum number of samples measured each time is 88, of which 18 are standard samples. All isotope values are reported relative to Pee Dee Belemnite (PDB) using standard delta notation; analytical precision for both δ^13^C and δ^18^O is better than 0.1‰. The weak relation between δ^18^O_carb_ and δ^13^C_carb_ (Pearson’s r = 0.108) indicates that the influence of diagenesis on the δ^13^C_carb_ signal appears to be negligible. 1307 samples from the Narao section were analyzed for δ^13^C_carb_, and integrated with published δ^13^C_carb_ data (*n* = 193)^24,25,67^, which were used for further study. Bulk δ^13^C_carb_ from the adjacent Naqing succession was also conducted to further validate the findings obtained from the Narao succession. For the Naqing section, 475 samples were analyzed for δ^13^C_carb_ and integrated with 245 published data points^24,67^. The δ^13^C_carb_ time series were calibrated using a published astronomical timescale^28^ for cyclostratigraphy analysis (Supplementary Fig. 10).

### Cyclostratigraphic analysis

A trend ( > 8 m) in the GR data was calculated using the LOWESS method (locally weighted scatterplot smoothing method) and subtracted (Supplementary Fig. 4a). It is essential to identify regular cyclicity in successions that lack high-resolution dating. We employed smoothed window averaging (SWA)^68^ to determine the spectral background. The SWA for red-noise spectra represents an empirical, non-parametric approach that makes no assumption about the underlying cause of the sloping spectral background^68^. The final results of the SWA background fitting for the time series power spectrum include the standard Chi-squared 99% confidence level. In cyclostratigraphic studies, confidence levels of >99.9% may effectively minimize the risk of Type I (false positive) errors; however, such excessively stringent confidence levels may increase the likelihood of Type II (false negative) errors^69^. In this study, the confidence levels of 99–99.9% were employed to validate the presence of significant cycles. These levels could balance the probability of incorrectly rejecting and incorrectly accepting a null hypothesis of no astronomical forcing^69^. Sliding-window spectral analysis was conducted using evolutive Fast Fourier Transform spectrograms (eFFT)^70^. These analyses were conducted in Acycle^71^.

Astronomical calibration was used to transform data from depth to time domains by fixing cycles to a constant duration. Calibration was conducted using the inferred 405-kyr eccentricity cycles. We conducted evolutionary time scale optimization (eTimeOpt)^35^ using Astrochron package^72^ to track the variable sediment accumulation rates throughout the Narao (Supplementary Fig. 4b–d). Long-term cycles modulate short-term ones according to astronomical theory^29^, and TimeOpt is a statistical method to test each sedimentation accumulation rate using this well-known phenomenon. Short eccentricity amplitude modulation was used here, with a frequency of 1/405 for the long eccentricity band, and frequencies of 1/132, 1/124, 1/100 and 1/95 for the short eccentricity bands (ZB23 solution^34^; Supplementary Fig. 3). The main procedure of eTimeOpt includes: (1) the detrended Narao GR data are filtered using Taner bandpass (low- and high- frequency cut-off was here used as 1/160 and 1/80, respectively), and Hilbert transformed to isolate the short eccentricity index amplitude envelope, which is then linearly regressed on a synthetic dataset with long eccentricity. The regression result is shown with the squared Pearson correlation coefficient r^2^_envelope_ (Supplementary Fig. 4b); and (2) the data are linearly regressed on a synthetic dataset with long and short eccentricity index frequencies, and the regression result is shown together with the squared Pearson correlation coefficient r^2^_power_ (Supplementary Fig. 4c). The product (r^2^_opt_ = r^2^_envelope_ × r^2^_power_) can combine information from both r^2^_envelope_ and r^2^_power_ (Supplementary Fig. 4d). The test sedimentation rates (*n* = 200) are from 0 to 2 cm/kyr, with sliding windows of 8 m.

Amplitude modulation (AM) analysis was conducted as follows^36^: (1) the 405-kyr-calibrated GR time series was bandpass-filtered to isolate the interpreted short eccentricity or obliquity signal, and (2) the amplitude modulations were obtained from the filtered signal by application of the Hilbert transform.

A relationship (e.g., cause and effect) could be evaluated using the multivariate time series analysis^70^. Cross-spectral analysis to determine the coherency and phasing between the two separated records was performed using the Blackman–Tukey cross-spectral analysis in Analyseries 2.0.8^73^. To identify the exact age for the changed phase relation, we conducted the cross-spectral analysis between GR and δ^13^C_carb_ using a Bartlett window, with sliding windows of 4 Myr and moving steps of 0.25 Myr. For the results with error bars >95% (or >90% and >85%), non-zero coherence is higher than 0.549641 (or 0.476565 and 0.425021), and the error estimation on the power spectrum is between 0.489097 and 3.06605 (or between 0.54708 and 2.52863, and between 0.590019 and 2.24219).

## Supplementary information

Supplementary Information

Transparent Peer Review file

## Data Availability

Source Data are provided with this paper. The data generated in this study are available at 10.5281/zenodo.17096002.

## Code availability

The Astrochron^72^ used in this study is available at https://CRAN.Rproject.org/package=astrochron. Acycle^71^ is available at 10.5281/zenodo.3955018.
