# Supplementary material for: Synchronizing climate-carbon cycle heartbeats in the Phanerozoic vegetated icehouses
Source: Nat Commun. 2025 Oct 16;16:9196. doi: 10.1038/s41467-025-64238-9 (PMC12533023; doi:10.1038/s41467-025-64238-9)
Supplement: Supplementary file 1 — Supplementary Information [file 41467_2025_64238_MOESM1_ESM.pdf]

Supplementary Materials for

Synchronizing climate-carbon cycle heartbeats in the Phanerozoic vegetated icehouses

Fang *et al.*

Corresponding author: Huaichun Wu, whcgeo@cugb.edu.cn

The PDF file includes:

Supplementary Text 2

Supplementary Figures 1 to 11

Supplementary Text 1. Carboniferous astrochronology

The Carboniferous geochronologic framework of GTS2020 was established using geological records of tropical Pangea[1]. Detailed U-Pb dating of conodont-rich successions in the Donets Basin[2,3] and southern Urals[4] enables the recognition of eccentricity-forced sedimentary cycles (i.e., “cyclothems”) spanning the late Carboniferous, providing a high-resolution time scale for the Gzhelian, Kasimovian, Moscovian stages[1]. However, the uppermost Serpukhovian and Bashkirian cyclothems[5] fail to conform to the 405-kyr pattern[1]. This may reflect stratigraphic hiatuses driven by a major eustatic fall, at the Mississippian/Pennsylvanian boundary (MPB) widely recorded in Euramerican carbonate platform successions. For instance, the GSSP section for the MPB in Arrow Canyon, Nevada, is punctuated by several subaerial hiatuses defining several fourth-order glacio-eustatic cycles[6-8]. In the nearby Marble Canyon succession, the mid-Carboniferous lowstand is recognized as parasequences capped by exposure features despite the outermost platform to upper slope setting[9]. In the Appalachian Basin, stacked palaeo-valleys are developed across the MPB[10]. Upper Silesian Basin from eastern Pangea records the U-Pb age calibrated cyclothems throughout most of the Serpukhovian, yet the absence of sediments across the lower and upper stage boundaries[11]. Thus, the numerical ages of basal Serpukhovian (330.3 ± 0.4 Ma) and Bashkirian (323.2 ± 0.4 Ma) in GTS2020 were subjected to the interpolation with cubic splines[1]. In contrast, for the Narao succession, astronomically calibrated, deep-marine sediments archive the basal boundaries of the Serpukhovian and Bashkirian with robust biostratigraphic constraints[12,13] that permitted astronomical calibration of these boundaries to 330.8 ± 0.3 Ma and 324.1 ± 0.3 Ma, respectfully.

Supplementary Text 2. Synchronicity in Carboniferous low-latitude glacio-eustasy

The record of onlap (transgression)–offlap (regression) from the Donets Basin, Ukraine[2] provides robust evidence for eccentricity-scale glacio-eustatic sea-level fluctuations from the Serpukhovian through end-Carboniferous[9,14,15]. This record reveals three periods of major sea-level fall across the mid-Carboniferous, early Moscovian, early Kasimovian and latest Gzhelian[2] (Fig. 3i) that coincide with increases in GR at Narao (black bars in Fig. 3f). Conversely, warming intervals inferred from paleotropical successions of North America[12] (red bars in Fig. 2i), are contemporaneous with low-latitude transgressions and high-latitude deglaciations[2,14,17], as well as the decrease in GR at Narao (red bars in Fig. 2f). One exception is the anomalous increase in terrestrial input during a CO_2-forced warming across the Kasimovian/Gzhelian boundary[17,18].

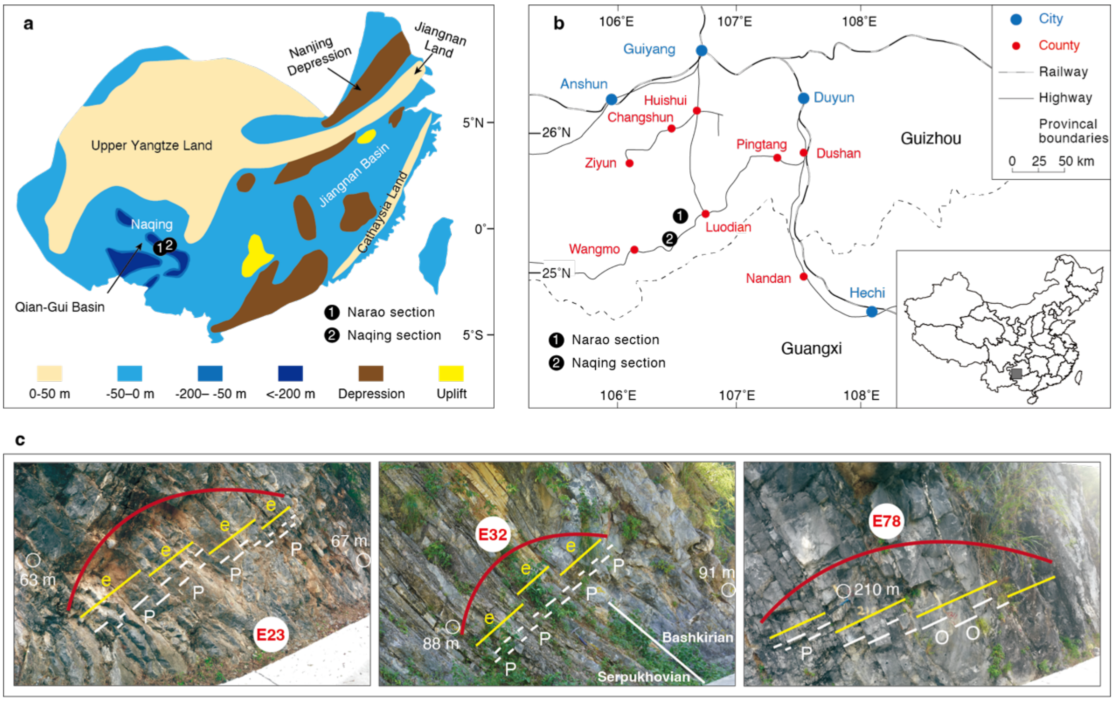

Supplementary Fig. 1. Geological and geographical backgrounds of the studied sections. a Lithofacies paleogeographic map of the South China block[19]. South China block straddled the paleo-equator along the eastern margin of the Paleo-Tethys Ocean during the Carboniferous–Permian time. **b** Location map of the Narao and Naqing sections of Luodian County, Guizhou province, China. Narao section is exposed along a lane close to Fengting County. **c** Milanković cycles recorded in the lithological combination in Narao section. Narao section consists of deep-marine slope deposits, including thin-bedded lime mudstones, intercalated with normal-graded intraclast-bearing wackestones to packstones. Five to six thin precession-scale beds ("P", white lines) are bundled into short orbital eccentricity cycles ("e", yellow lines), and four short eccentricity cycles are bundled into a long orbital eccentricity cycle ("E", red curve). The obliquity cycle ("O", white lines) dominates the E78 cycle.

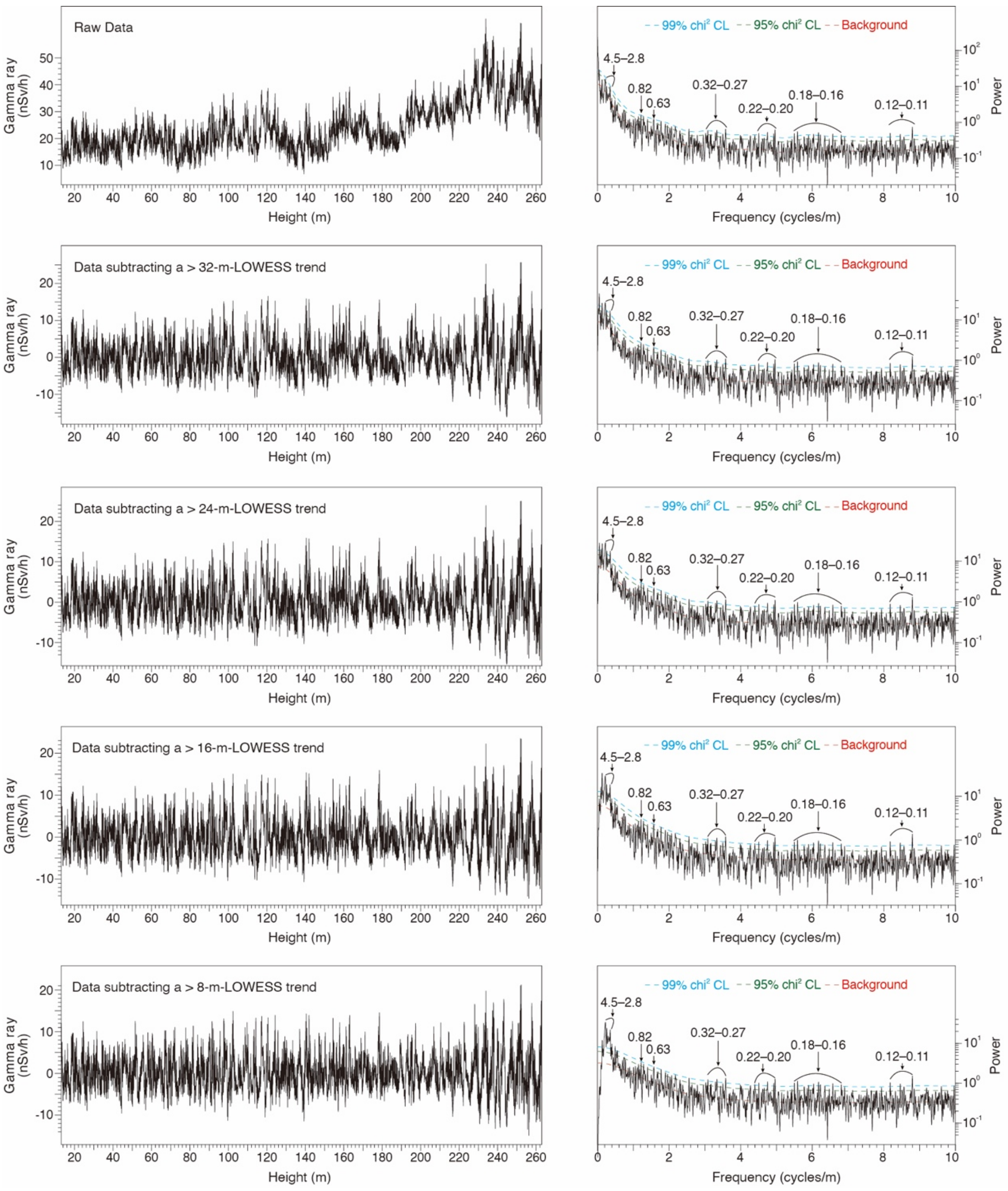

Supplementary Fig. 2. Different detrending processes and their impact on spectral analysis. Raw data and data subtracting a >32-m, >24-m, >16-m >8-m LOESS trend (left part), and their smoothed window averaging (SWA) background fitting for the power spectrum (right part), including the standard Chi-squared 99% and 95% confidence levels (CLs).

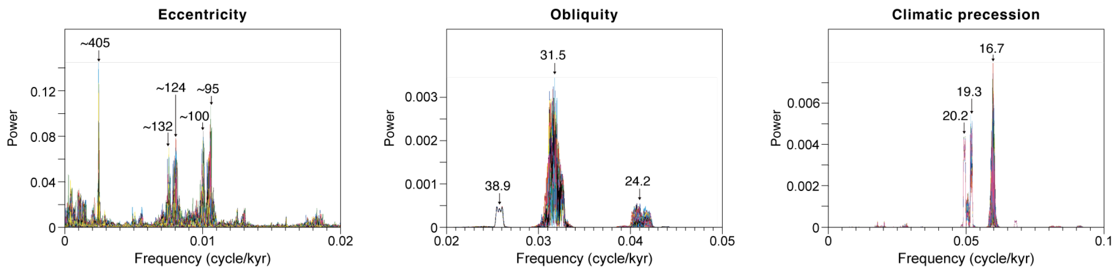

Supplementary Fig. 3. Periods of eccentricity, obliquity and climatic precession for a time interval of 337–300 Ma. There are 64 solutions from ZB23 solution[20] that used here to calculate the periods of eccentricity, obliquity and climatic precession cycles. Numbers in the figure were labelled in kiloyear.

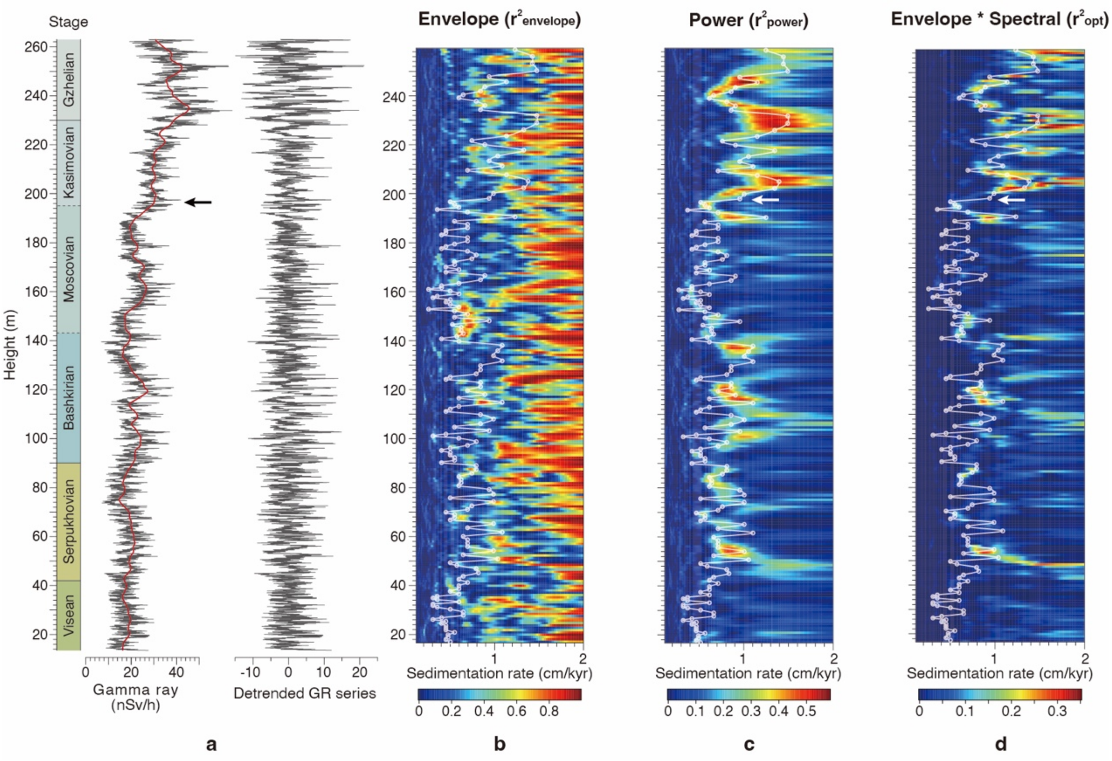

Supplementary Fig. 4. Sedimentation rate obtained from evolutionary time scale optimization (eTimeOpt). a Raw Narao GR data and detrended GR data after deleting an 8-m LOWESS curve. **b–d** Evolutive TimeOpt of GR data. The gray line is the calculated sedimentation rate from 405-kyr astronomical calibration. The arrows indicate a sudden increase in the sedimentation rate, which corresponds to the elevated levels of GR values.

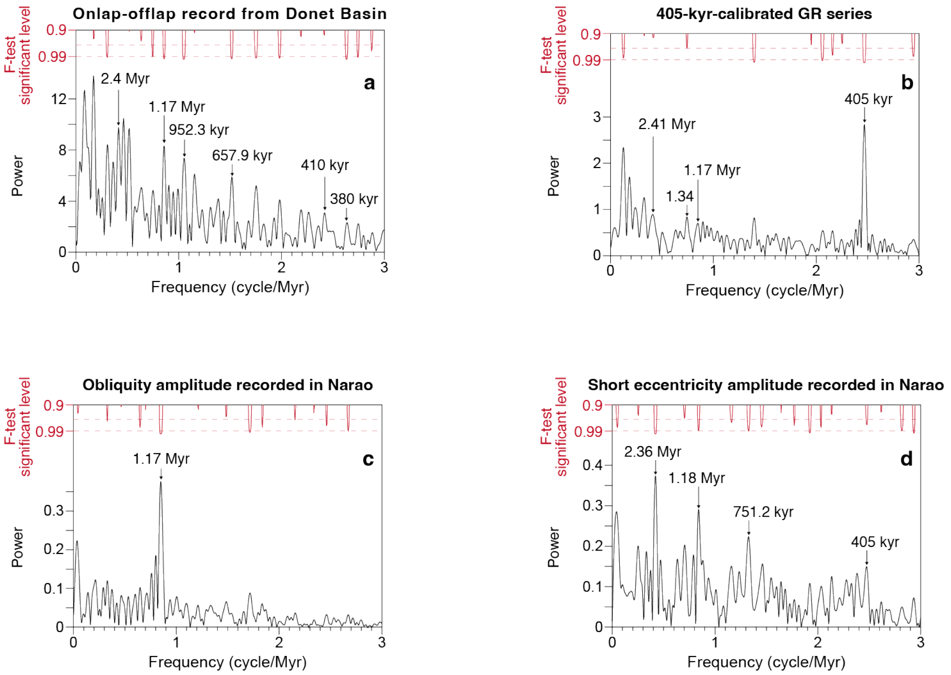

Supplementary Fig. 5. Periodograms (black curves) and *F*-test values (red curves; top) of the geological datasets during the Late Paleozoic Ice Age. A periodogram offers higher resolution compared to the MTM power spectrum[21]. We have combined these periodograms with *F*-test to test for the presence of regular cyclic signal components. All analyses were conducted in Acycle[22]. **a** Onlap–offlap history of the Donets Basin, Ukraine[2] (data shown in Fig. 2i). **b** 405-kyr-calibrated GR series from Narao (data shown in Fig. 2f). **c** Obliquity amplitude recorded in Narao section (data shown in Fig. 2h). **d** Short eccentricity amplitude recorded in Narao section (based on data shown in Fig. 2g).

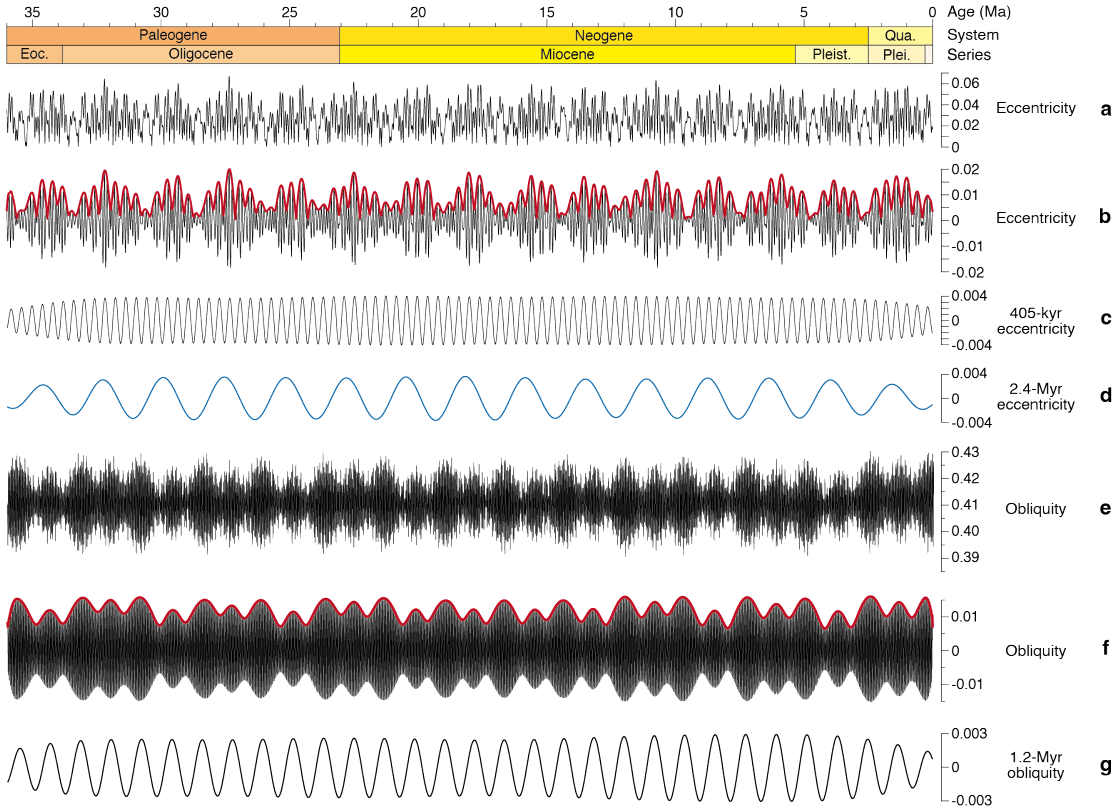

Supplementary Fig. 6. The solution of Earth's astronomical parameters (ZB18a) for the past 36 Myr. a Raw eccentricity signal from ZB18a solution[23]. **b** Amplitude modulation (red curve) of ~100-kyr short eccentricity band (black curve), extracted using a bandpass of 9 ± 2 Myr^{-1}. **c** 405-kyr eccentricity cycle signal, extracted using a bandpass of 2.46 ± 0.1 Myr^{-1} from the amplitude modulation of ~100-kyr short eccentricity cycle. **d** 2.4-Myr eccentricity cycle signal, extracted using a bandpass of 2.46 ± 0.1 Myr^{-1} from the amplitude modulation of ~100-kyr short eccentricity cycle. **e** Raw obliquity signal from ZB18a solution. **f** Amplitude modulation (red curve) of ~40-kyr obliquity band (black curve), extracted using a bandpass of 25 ± 1 Myr^{-1}. **g** 1.2-Myr obliquity cycle signal, extracted using a bandpass of 0.85 ± 0.1 Myr^{-1} from the amplitude modulation of ~40-kyr obliquity cycle.

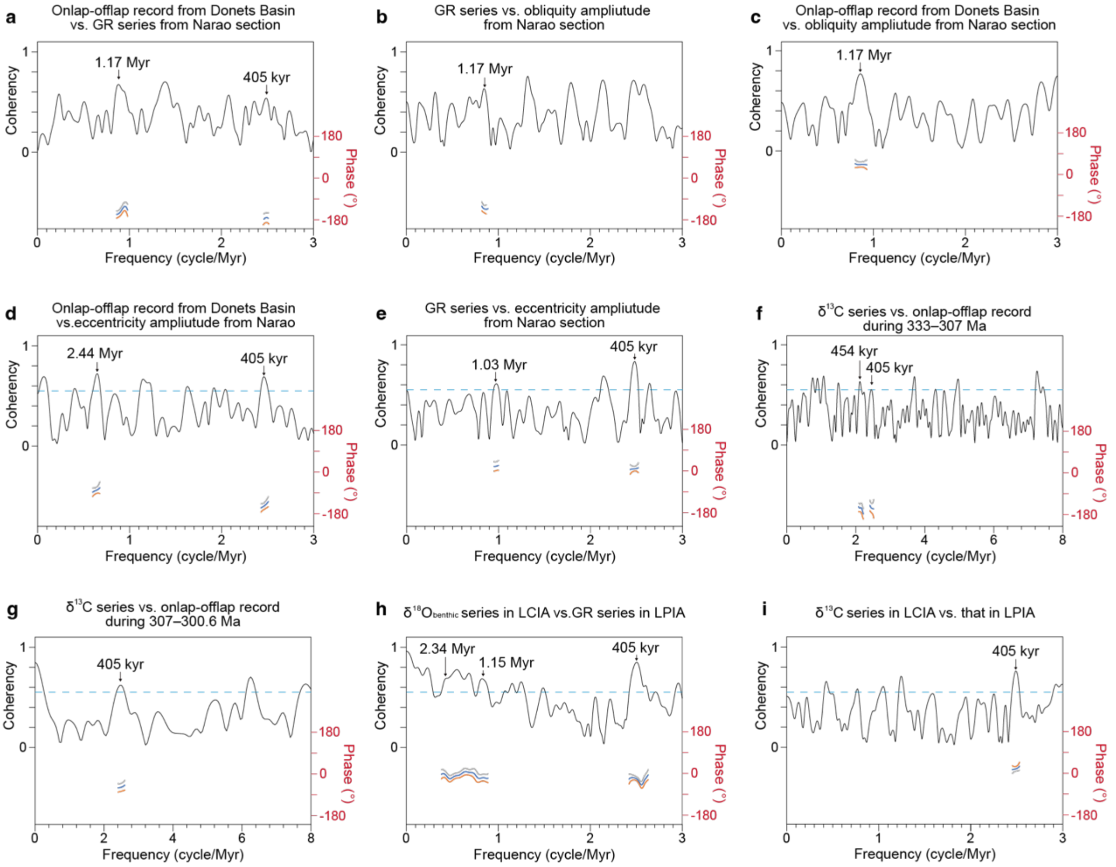

Supplementary Fig. 7. Coherency and cross-phase spectral analysis between datasets. a Onlap-offlap curve from Donets Basin (data shown in Fig. 3i) versus astronomically calibrated GR series from Narao (data shown in Fig. 3f). **b** Astronomically-calibrated GR series versus amplitude modulation of ~31-kyr obliquity cycle (data shown in Fig. 3h). **c** Onlap-offlap curve versus amplitude modulation of ~31-kyr obliquity cycle. **d** Onlap-offlap curve versus amplitude modulation of ~100-kyr obliquity cycle (data shown in Fig. 3g). **e** Astronomically-calibrated GR series versus amplitude modulation of ~100-kyr eccentricity cycle. **f** Astronomically-calibrated $\delta^{13}C_{carb}$ series from Narao (data shown in Fig. 3e) versus onlap-offlap curve from Donets Basin (data shown in Fig. 3i) during 333–307 Ma. **g** Astronomically-calibrated $\delta^{13}C_{carb}$ series from Narao (data shown in Fig. 3e) versus onlap-offlap curve from Donets Basin (data shown in Fig. 3i) during 307–300.6 Ma. **h** $\delta^{18}O$ series during LCIA (36–0 Ma; data shown in Fig. 4a_7) versus GR series during LPIA (337–301 Ma; data shown in Fig. 4b_7). **i** $\delta^{13}C$ series during LCIA (36–0 Ma) versus $\delta^{13}C$ series during LPIA (337–301 Ma). All the analyses were conducted on the *Analyseries 2.0.8*. Blackman-Tukey spectrum uses a Bartlett window with a bandwidth of 0.188679 Myr^{-1}. Non-zero coherence is higher than 0.552511. The error estimation on the power spectrum is between 0.487194 and 3.09553.

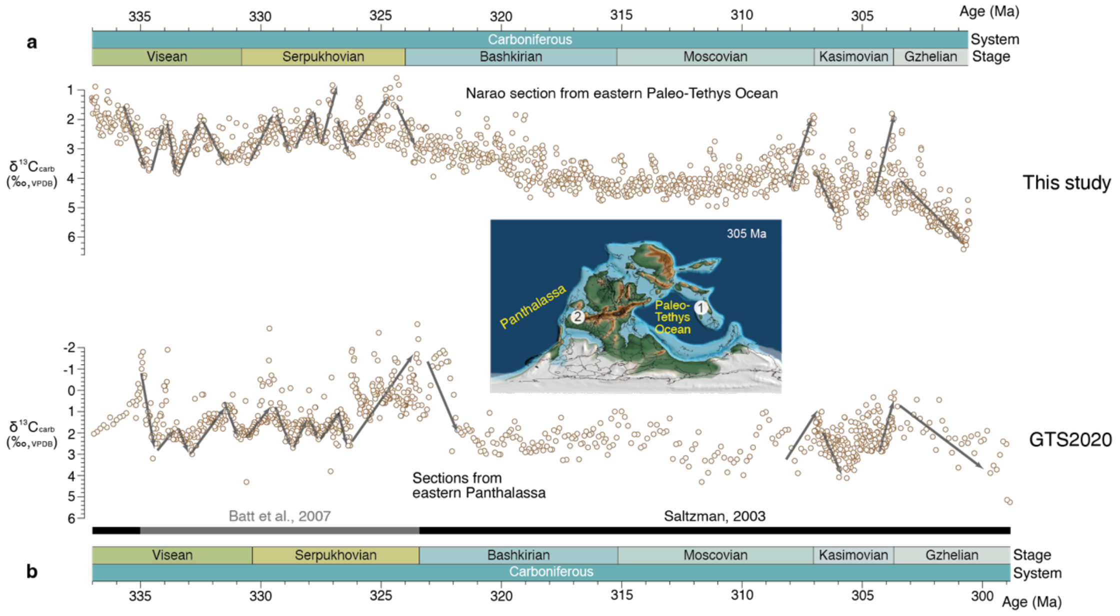

Supplementary Fig. 8. Global δ^{13}C chemostratigraphic correlation during the Carboniferous. a δ^{13}C$_{carb}$ data from Narao section from the eastern Paleo-Tethys Ocean, calibrated by newly constructed astronomical timescale in this study. **b** Composite δ^{13}C$_{carb}$ data from the sedimentary successions in the eastern Panthalassa[24,25], calibrated by the GTS2020 time scale[1]. Inserted graph[26] showing the paleogeography of the sedimentary successions.

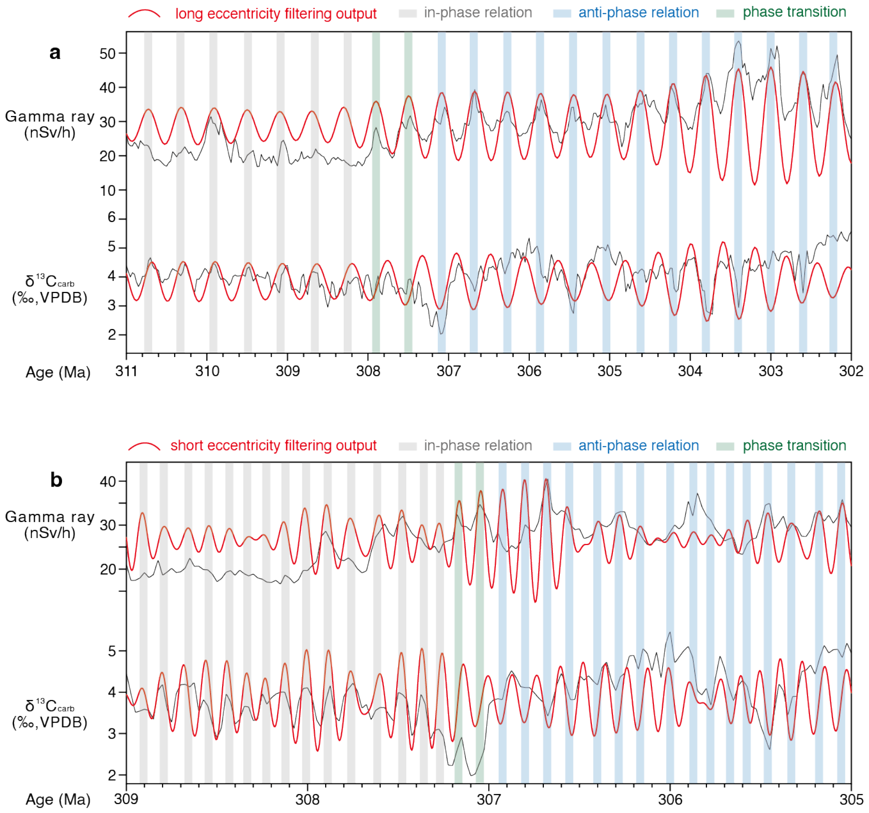

Supplementary Fig. 9. Phase relation between GR and δ^{13}C data from the Narao section on short and long eccentricity bands. **a** Phase relation between GR and δ^{13}C data on long eccentricity bands during 311−302 Ma. 405-kyr signals (red curves) were filtered using a passband of 2.46 ± 0.3 Myr^{-1}. b Phase relation between GR and δ^{13}C data on short eccentricity bands during 309−305 Ma. Short eccentricity signals (red curves) were filtered using a passband of 8.5 ± 1.5 Myr^{-1}. The in-phase behaviour inverts to anti-phase at ~307 Ma.

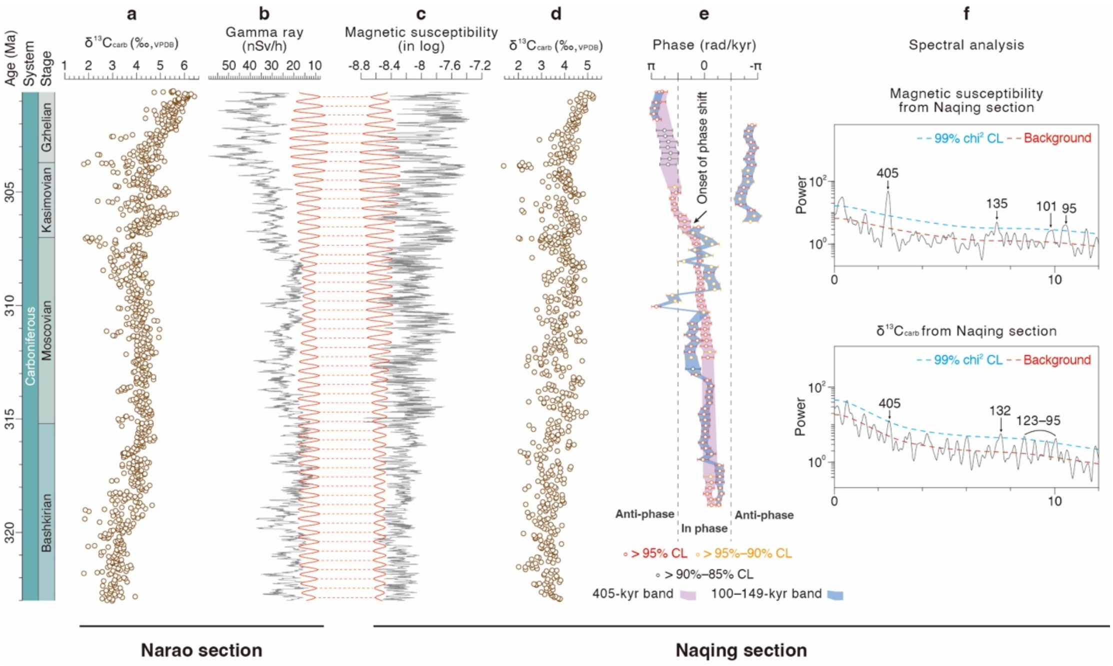

Supplementary Fig. 10. Climate-carbon cycle relation on eccentricity timescales from 322.8 to 300.6 Ma, recorded in the Naqing section. a $\delta^{13}C_{carb}$ data from Narao section in this study. **b** GR data from Narao. **c** 405-kyr-calibrated magnetic susceptibility from Naqing section[27]. All the 405-kyr eccentricity cycles (red curves) were extracted using 2.46 ± 0.2 Myr^{-1} bandpass. **d** 405-kyr-calibrated $\delta^{13}C_{carb}$ data from Naqing section (see "Methods" section). **e** Evolution of phase-relationship between magnetic susceptibility and $\delta^{13}C_{carb}$ on 405-kyr (purple area) and 95–140-kyr (blue area) eccentricity time scales, with the 4-Myr-width windows and 0.25-Myr steps. An abrupt shift from in-phase to anti-phase behaviour was observed at ~307 Ma. **f** Smoothed window averaging (SWA) background fitting for the power spectra of MS and $\delta^{13}C_{carb}$ from the Naqing section, including the standard Chi-squared 99% confidence level (CL).

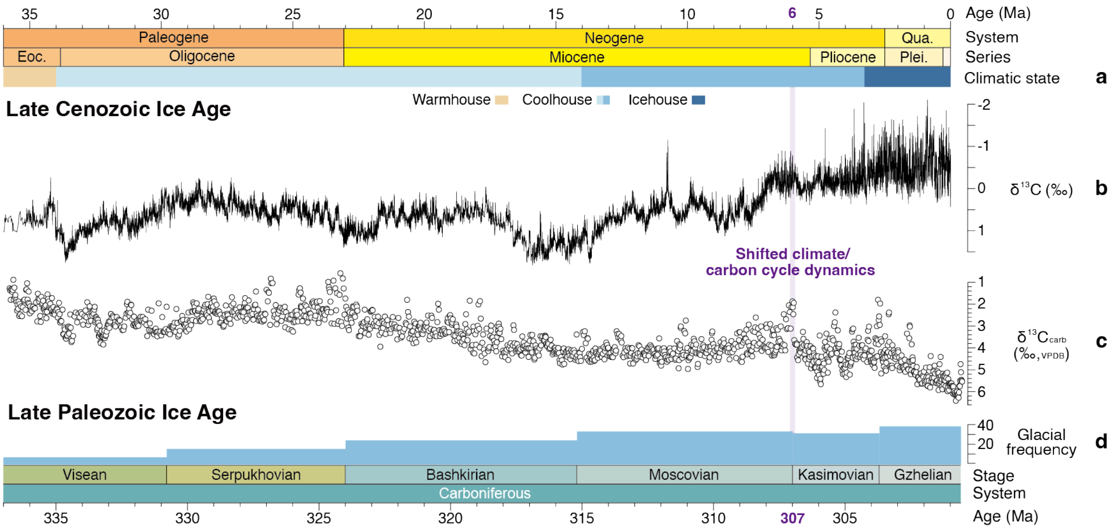

Supplementary Fig. 11. The paralleled carbon isotope series during the key stages of the Late Cenozoic Ice Age (LCIA) and Late Paleozoic Ice Age (LPIA). Purple shading indicates the times (6 Ma for LCIA and 307 Ma for LPIA) of the shifted climate/carbon cycle dynamics. **a** Climate state[28]. **b** Cenozoic global reference benthic foraminifer $\delta^{13}C$[28]. **c** 405-kyr-calibrated $\delta^{13}C$ series at Narao. **d** Glacial frequency[29].

Supplementary References

1 Aretz, M. et al. in *Geologic Time Scale 2020* (eds Felix M. Gradstein, James G. Ogg, Mark D. Schmitz, & Gabi M. Ogg) 811–874 (Elsevier, 2020).

2 Eros, J. M. et al. Sequence stratigraphy and onlap history of the Donets Basin, Ukraine: insight into Carboniferous icehouse dynamics. *Palaeogeogr. Palaeoclimatol. Palaeoecol.* **313**, 1–25 (2012).

3 Davydov, V. I., Crowley, J. L., Schmitz, M. D. & Poletaev, V. I. High-precision U-Pb zircon age calibration of the global Carboniferous time scale and Milankovitch band cyclicity in the Donets Basin, eastern Ukraine. *Geochem. Geophys. Geosyst.* **11**, Q0AA04 (2010).

4 Schmitz, M. D. & Davydov, V. I. Quantitative radiometric and biostratigraphic calibration of the Pennsylvanian–Early Permian (Cisuralian) time scale and pan-Euramerican chronostratigraphic correlation. *Geol. Soc. Am. Bull.* **124**, 549–577 (2012).

5 Heckel, P. Pennsylvanian stratigraphy of Northern Midcontinent Shelf and biostratigraphic correlation of cyclothems. *Stratigraphy* **10**, 3–39 (2013).

6 Barnett, A. J. & Wright, V. P. A sedimentological and cyclostratigraphic evaluation of the completeness of the Mississippian–Pennsylvanian (Mid-Carboniferous) global stratotype section and point, Arrow Canyon, Nevada, USA. *J. Geol. Soc.* **165**, 859–873 (2008).

7 Lane, H. R., Brenckle, P. L., Baesemann, J. & Richards, B. The IUGS boundary in the middle of the Carboniferous: Arrow Canyon, Nevada, USA. *Episodes* **22**, 272–283 (1999).

8 Bishop, J. W., Montañez, I. P., Gulbranson, E. L. & Brenckle, P. L. The onset of mid-Carboniferous glacio-eustasy: Sedimentologic and diagenetic constraints, Arrow Canyon, Nevada. *Palaeogeogr. Palaeoclimatol. Palaeoecol.* **276**, 217–243 (2009).

9 Martin, L. G., Montañez, I. P. & Bishop, J. W. A paleotropical carbonate-dominated archive of Carboniferous icehouse dynamics, Bird Spring Fm., Southern Great Basin, USA. *Palaeogeogr. Palaeoclimatol. Palaeoecol.* **329**, 64–82 (2012).

10 Blake, B. M. & Beuthin, J. D. Deciphering the mid-Carboniferous eustatic event in the central Appalachian foreland basin, southern West Virginia, USA. *Geol. Soc. Am.* **441**, 249–260 (2008).

11 Opluštil, S. et al. Coal-bearing fluvial cycles of the late Paleozoic tropics; astronomical control on sediment supply constrained by high-precision radioisotopic ages, Upper Silesian Basin. *Earth-Sci. Rev.* **228**, 103998 (2022).

12 Tian, X. X. et al. Glacio-eustasy and $\delta^{13}C$ across the Mississippian–Pennsylvanian boundary in the eastern Paleo-Tethys Ocean (South China): Implications for mid-Carboniferous major glaciation. *Geol. J.* **55,** 2704–2716.(2019).

13 Chen, J. T. et al. Coupled sedimentary and $\delta^{13}C$ records of late Mississippian platform-to-slope successions from South China: Insight into $\delta^{13}C$

chemostratigraphy. *Palaeogeogr. Palaeoclimatol. Palaeoecol.* **448**, 162–178 (2016).
14 Montañez, I. P. Current synthesis of the penultimate icehouse and its imprint on the Upper Devonian through Permian stratigraphic record. *Geol. Soc. Lond. Spec. Publ.* **512**, 213–245 (2022).
15 Montañez, I. P. et al. Carboniferous climate teleconnections archived in coupled bioapatite $\delta^{18}O_{PO4}$ and $^{87}Sr/^{86}Sr$ records from the epicontinental Donets Basin, Ukraine. *Earth Planet. Sci. Lett.* **492**, 89–101 (2018).
16 Davydov, V. Warm water benthic foraminifera document the Pennsylvanian–Permian warming and cooling events—The record from the Western Pangea tropical shelves. *Palaeogeogr. Palaeoclimatol. Palaeoecol.* **414**, 284–295 (2014).
17 Chen, J. T. et al. Marine anoxia linked to abrupt global warming during Earth's penultimate icehouse. *Proc. Natl. Acad. Sci. USA* **119**, e2115231119 (2022).
18 Richey, J. D. et al. Influence of temporally varying weatherability on CO_2-climate coupling and ecosystem change in the late Paleozoic. *Clim. Past* **16**, 1759–1775 (2020).
19 Wang, Y. & Jin, Y. G. Permian palaeogeographic evolution of the Jiangnan Basin, South China. *Palaeogeogr. Palaeoclimatol. Palaeoecol.* **160**, 35–44 (2000).
20 Zeebe, R. E. & Lantink, M. L. Milanković Forcing in Deep Time. *Paleoceanogr. Paleoclimatol.* **39**, e2024PA004861 (2024).
21 Kodama, K. P. & Hinnov, L. A. *Rock Magnetic Cyclostratigraphy*. 1–165 (John Wiley & Sons, 2015).
22 Li, M. S. , Hinnov, L. & Kump, L. Acycle: Time-series analysis software for paleoclimate research and education. *Comput. Geosci.* **127**, 12–22 (2019).
23 Zeebe, R. & Lourens, L. Geologically constrained astronomical solutions for the Cenozoic era. *Earth Planet. Sci. Lett.* **592**, 117595 (2022).
24 Batt, L. S. et al. Multi-carbonate component reconstruction of mid-Carboniferous (Chesterian) seawater $\delta^{13}C$. *Palaeogeogr. Palaeoclimatol. Palaeoecol.* **256**, 298–318 (2007).
25 Saltzman, M. R. Late Paleozoic ice age: Oceanic gateway or pCO_2? *Geology* **31**, 151–154 (2003).
26 Scotese, C. R. An atlas of Phanerozoic paleogeographic maps: the seas come in and the seas go out. *Annu. Rev. Earth Planet. Sci.* **49**, 679–728 (2021).
27 Wu, H. C. et al. An ~34 m.y. astronomical time scale for the uppermost Mississippian through Pennsylvanian of the Carboniferous System of the Paleo-Tethyan realm. *Geology* **47**, 83–86 (2019).
28 Westerhold, T. et al. An astronomically dated record of Earth's climate and its predictability over the last 66 million years. *Science* **369**, 1383–1387 (2020).
29 Soreghan, G. S., Soreghan, M. J. & Heavens, N. G. Explosive volcanism as a key driver of the late Paleozoic ice age. *Geology* **47**, 600–604 (2019).
