# Supplementary material for: Synchronizing climate-carbon cycle heartbeats in the Phanerozoic vegetated icehouses
Source: Nat Commun. 2025 Oct 16;16:9196. doi: 10.1038/s41467-025-64238-9 (PMC12533023; doi:10.1038/s41467-025-64238-9)
Supplement: Supplementary file 2 — Transparent Peer Review file [file 41467_2025_64238_MOESM2_ESM.pdf]

nature portfolio

Peer Review File

Synchronizing climate-carbon cycle heartbeats in the Phanerozoic vegetated icehouses

Corresponding Author: Professor Huaichun Wu

Parts of this Peer Review File have been redacted as indicated to remove third-party material.

This file contains all reviewer reports in order by version, followed by all author rebuttals in order by version.

Attachments originally included by the reviewers as part of their assessment can be found at the end of this file.

Version 0:

Reviewer comments:

Reviewer #1

(Remarks to the Author)
In their manuscript (ms), Fang et al. present new gamma ray (GR) and d13C bulk-rock carbonate measurements of the upper Carboniferous Narao section (China) to investigate astronomical climate forcing and carbon cycle dynamics during the Late Paleozoic Ice Age. Using time-series analysis, the authors identify a shift from in-phase to anti-phase behavior between variations in GR (a proxy for glacio-eustatic sea level (SL) change) and d13C on eccentricity timescales around the Moscovian-Kasimovian boundary (~307 Ma), that is, from coupled low SL-high d13C to low SL-low d13C values. This shift is interpreted as a reorganization of Earth's climate-carbon cycle system in response to orbital forcing – from a growing terrestrial biosphere during glacial intervals to a shrinking terrestrial biosphere during glacial intervals (?) – analogous to the shift from in-phase to antiphase behavior between benthic foraminifera d18O and d13C during the Late Cenozoic.

The proposed phase shift and analogy to the Late Cenozoic Ice Age is fascinating, if true. However, I see a couple of potential issues that need to be addressed first and/or require further clarification before I can recommend publication:

1. Insufficient info on d13C. At the moment, I cannot properly assess the robustness of the proposed phase relation shift between GR and d13C on eccentricity timescales, because no results of cyclostratigraphic analysis (Milanković hypothesis testing) are presented for the Narao d13C record (at least as far as I am aware). If the goal is to investigate phase relationships between changes in global SL/ice volume and the carbon cycle in response to orbital forcing, one also needs to show (convincingly) that both components are indeed controlled by orbital forcing in the first place. In addition, the authors need to provide more information about the sampling strategy – in particular, how are d13C sample depths related to GR depths? Currently, the Methods section mentions that 5000 GR data points from the Narao section were taken "..with an average sample distance of 0.05 m", and a total of 1307 samples for d13C (lines. 473-477). In the Excel data file, however, I find d13C data interpolated also at 0.05 m steps, so no raw depths are given. What was the original sampling density of d13C, and how is it related to GR? If, for example, d13C data are offset from GR by 1-2 m, this could artificially introduce a change from in-phase to anti-phase behavior. Also, I would recommend to include a figure in the ms that shows to readers a zoom-in of a typical/well-expressed eccentricity cycle in GR and d13C from where these proxies are in phase, and one from where they are out of phase. (So in addition to the phase-relationship plot in Fig. 3j).

2. Uncertainty in the astronomical timescale –> this should definitely get more attention in the ms (currently uncertainty is not discussed at all). Specifically, what would be the consequences of errors in the astrochronologic framework for the inferred phase relationships? First, tuning was done based on the long (~405 kyr) eccentricity cycle, which the authors refer to as ".. a stable astronomical component throughout the geological history." However, see the paper by Zeebe and Lantink (2024), which shows that the long eccentricity cycle can become unstable on 10^8-10^9 year scales. Second, I find it very hard to believe that a complete record of Milanković forcing of ~37 Myrs long is preserved in a single section, especially considering the nature of the sedimentary environment ("deep-marine slope deposits", see l. 602). I think the longest deep-sea (composite) record used in Westerhold et al. (2020) is about ~20 Myr. The Newark-Hartford record of Olsen et al (2019) is also on that order; astronomical signal quality and the density of independent chronostratigraphic constraints is much higher for these records than is the case for the Narao data (which has zero independent U-Pb constraints…). What about the

possibility of turbidites or hiatuses associated with sea level change? In Extended Data Fig. 1c, I can clearly see irregular bedding above cycle E7 and E32. In Fig. 2, the EHA plot shows a disruption of the (inferred) long eccentricity-scale signal around 0-30 m and ~165-185 height (just before the inferred phase shift occurs).

3. A global signal. It's a big leap from GR and d13C bulk-rock carbonate data of one section (Narao) to interpreting changes in the global climate-carbon cycle. The GR and d13C curves from the Narao section have been correlated to time-equivalent records from Donets and the GTS2020 site, indicating they may be representative for global-scale processes (Fig. 3i, Extended Data Fig. 7). However, the Donets and GTS datasets are of a lower resolution and have not been analyzed at (and correlated on) Milanković timescales. So while the general trend in the Narao series may indeed reflect a global signal, I wouldn't dare to say that the correlation to Donets and GTS provides direct support for global-scale climate-carbon cycle processes on orbital timescales. (Cyclostratigraphic study has supposedly been conducted also one data from the lateral Naqing section – see l. 136-137 and Extended Data Fig. 8 - but the actual analyses are not shown in the current ms and a precise cyclostratigraphic correlation to Narao is also lacking).

Minor points:

l. 42-43: "low-latitude continental carbon reservoirs expanded mostly during the astronomically-forced cool intervals (4) -> Is the reference to Kirkland-Turner (2014) appropriate here? Because from my understanding, that paper talks about a "gradual expansion of vegetated terrestrial biosphere during warm intervals".

l. 65 "to test this hypothesis" -> elaborate.

l. 74-75: how is it possible that no significant short eccentricity-scale peaks are identified in the raw depth series, only after tuning to a 405 kyr cycle?

l.83-86: "The calculated change in sedimentation rate…tracks with those obtained from two objective statistical methodologies, i.e.,…. and evolutionaly correlation coefficient." I've attached a document to this review from prof. Stephen Meyers (personal comm.). In this document Meyers explains why he thinks that the eCOCO method of Li et al. (2018) is unfortunately flawed. Meyers has given me permission to share this document with the authors of the present ms. I haven't discussed the current ms (Fang et al.) with Meyers.

l. 93-96: the ~1.2 and ~2.4 Myr Grand cycles are not stable in deep time.

l. 108-111: I don't understand how the long-term obliquity AM cycle can show an anti-phase relation with the Narao GR series and in-phase relation with the onlap-offlap record of the Donets, if Donets and Narao GR record display a constant antiphase relationship (l. 105-106).

l. 134-135: "This shifted phase relation is delayed by ~0.5 Myr for the 95-132 kyr eccentricity band.." -> in Extended Data Fig. 2, I see no concentration of spectral lower in the short eccentricity range for Kasimovian-Gzehlian. Are the result of phase analysis then reliable for this interval?

References:

Olsen, Paul E., Jacques Laskar, Dennis V. Kent, Sean T. Kinney, David J. Reynolds, Jingeng Sha, and Jessica H. Whiteside. "Mapping solar system chaos with the Geological Orrery." Proceedings of the National Academy of Sciences 116, no. 22 (2019): 10664-10673.

Zeebe, R. E., & Lantink, M. L. (2024). A Secular Solar System Resonance that Disrupts the Dominant Cycle in Earth's Orbital Eccentricity (g 2– g 5): Implications for Astrochronology. The Astronomical Journal, 167(5), 204.

Reviewer #2

(Remarks to the Author)
This study presents an impressive record of novel bulk carbonate δ18O measurements, with a new astronomical time scale based on gamma-ray data. The authors argue that the Late Paleozoic Ice Age shows interesting parallels to our current glacial world, and that this record can be used to test this hypothesis. I think the work is significant and warrants publication.

However, there are some major issues with the astronomical solutions and data analysis that should be addressed, requiring major revisions.

See attached PDF for the Major and Minor comments. I have also included a quick reproduction of the spectral analysis.

[Editorial Note: The PDF has been appended to the end of this file]

Reviewer #3

(Remarks to the Author)

The manuscript uses a new dataset of densely sampled marine carbon isotopes from the Carboniferous of China to argue for the cyclicity of orbital forcing to be driving the patterns and then draws comparison between Paleozoic and Cenozoic ice ages to argue that have a similar time course due to their shared influence of orbital forcing. It is a provocative perspective and was interesting to read, but it felt increasingly tenuous as the manuscript progressed and the later sections took off at a gallop with more and more connections and new ideas thrown in, each stated as a positive truth without distinguishing between what was directly observable in the rock record versus a modeling-based suggestion or what was a global pattern versus a local pattern, and with no reflection on what it would mean if any of the assertions expressed with certainty happened not to be true. And once the end was reached it was surprising to see that there never was acknowledgment of all the other factors that might have been driving climate that were left untouched or that it was a choice to truncate the length of the Paleozoic ice age to match up with the time course of the Cenozoic ice age and there was no justification of why that choice was appropriate. Finally, much of the material is far from my expertise and there were many interesting assertions that were new to me backed up by literature that I didn't know, so I had homework to do. And in following up with those references, they were not always appropriate for how they were being used. Honestly, the manuscript would be stronger if the second half was cut off and the authors focused in on the isotopic record, the potential periodicity in that record, and comparison to Cenozoic glacial forcing.

First, it is true that there has been a big change in perspective in recent years regarding the causes of glaciation. In previous decades, continental configurations and how they channeled ocean currents with their heat transport and salinity differences were given great emphasis with the opening of the Southern Ocean and the closing of the Isthmus of Panama. Recent literature has focused much more heavily on CO2 as the key driver above all else. So, the emphasis here has precedent, and yet the comparison is between the Cenozoic (when continents have been maximally dispersed) and the Carboniferous (with Euramerica and the coalbeds discussed in this paper being the key link in the assembly of Pangea). Shouldn't there be some discussion of Pangea versus Cenozoic geography, even if just to argue that it doesn't matter and justify why? At the least the geometry of Pangea should matter for the discussion of the changing latitudes of coal deposits—in part, that reflects where there was land in a pangean world.

As another example of what is not considered, the authors draw their record of glaciation through the Carboniferous from reference 55 (Soreghan et al. 2019), but that paper is an argument that volcanism is a crucial driver of Paleozoic volcanism —as has also been argued for the Ordovician glaciation in recent years. And yet, there is no mention of volcanism at all in the current manuscript. It fits into a general concern with the manuscript in that arguments build out further and further in a straight line—culminating in orbital forcing causing both human and tetrapod evolution—but there never seems to be consideration of what it would mean if any of the logical connections in the series might be wrong or incomplete.

Another bigger thing to consider is that the manuscript is built around the comparison of Cenozoic and Carboniferous time series and how both go through a transition about 7 Myr before the end, and there is even the fortuitous simplicity of the durations and dates matching up with 35-0 Ma for the Cenozoic and 335-300 Ma for the Carboniferous. The explicit sentence in the Abstract is "Paralleling LPIA (335–301 Ma) and LCIA (34 Ma to the present) records using this event, we reveal the quasi-synchronization in the interaction of astronomical forcing, carbon cycling and glacial events from the onset to apex of two icehouses." However, only one of those time series is complete. That Soreghan reference source that is used has glaciation continually from the Late Devonian through most of the Permian. How could there be quasi-synchronization from onset to apex if the Paleozoic onset is about 30 Myr earlier than the truncated onset of the include time series and the Paleozoic peak is in the Permian just after the manuscript's time series ends. Overall, the included Paleozoic time series only about 40% of the total time series and includes neither onset nor apex.

And finally, there is a series of problematic referencing that adds to the tenuousness:
"Net positive terrestrial carbon sequestration occurred during late Carboniferous glacials driven by the predominance of wetland forests (13)." (Lines 58-60)
"A loss of arborescent lycopsids would decrease the terrestrial carbon burial rates at low latitudes during glacial periods (13), which would have shifted the centre of terrestrial carbon burial from low to high latitudes." (Lines 156-158)
None of that is really in reference 13. The only mention of glaciation is a reference to the same DiMichele 2014 paper that is already reference 10 in this manuscript (although incorrectly cited as DiMichele & Herendeen 2014). And no claim is made that lycopsids are uniquely important; instead it is emphasized that carbon sequestration remains high once the transition occurs to tree fern dominance.

"At ~307 Ma, most lycopsids abruptly disappeared (14), which potentially invokes a significant change in the global carbon cycle to orbital forcing." (Lines 60-61)
This is cast as a global statement, but that reference is specific to Peoria, Illinois as a local venue to speak about Euramerica. What about all of the lycopsids that persisted in eastern Pangea and China?

"Both Earth System modeling (33) and empirical records (35) reveal that shifts in tundra and boreal forest ecosystems occurred in response to orbitally driven climatic change during the late Carboniferous. Global carbon cycle dynamics may have been affected by such changes in land cover given major differences in the carbon storage capacities between the high-latitude biomes (33)." (Lines 160-164)
That empirical record 35 is from a single place and time, how could it record a shift? And no mention is made of orbital forcing in 35. Reference 33 does address the topic of that first sentence regarding tundra versus forest and orbital forcing, but it does not touch on the carbon storage of that second citation. However, even the first 33 reference feels like a misrepresentation as far as the way that reference is used. The modeling employs modern biome characteristics and applies them to the Paleozoic even though the conifers, flowering plants, and even the lichens of those modern biomes had not

evolved in the Paleozoic. What they did in that paper was correct in the context of that paper, which was show that they couldn't get the glacial cycling without including a dynamic vegetation model, but that does not amount to endorsing a particular vegetational distribution from model outputs or that the characteristics of the vegetation in the model reflects reality.

"First, Carboniferous climate simulations reveal that ocean circulation strengthened during glacials (36). Stronger ocean circulation during glacial phases would have favored enhanced transfer of organic matter to deep waters via the biological pump, causing a decrease in deep-sea δ13CDIC. (Lines 170-173)
Why would stronger circulation enhance the biological pump? Is this about nutrient upwelling? No explanation is offered and that the reference included with the lead-in sentence does not mention the biologic pump.

"Third, reduced atmospheric pCO2 would have had a profound impact on the paleo-plants' physiological forcing on the hydrological cycle that would have altered weathering patterns (38) and regional marine carbon burial." (Lines 178-181)
Weathering comes up only once, and it is an aside mention that if evapotranspiration decreased, then runoff would increase and maybe that would affect weathering. But, as they note, that would only be true if precipitation does not change with evapotranspiration, when surely it does. So, again, there is no relevance here.

Version 1:

Reviewer comments:

Reviewer #1

(Remarks to the Author)
The various actions that the authors have taken to address my concerns are comprehensive, which I appreciate.

I'm still intrigued by the observation that the inferred (abrupt) change from in-phase to anti-phase relation between d13C and gamma ray signal around ~189 m height (~307 Ma) coincides almost exactly with an abrupt change (i.e., increase) in inferred sedimentation rate (according to the cyclostratigraphic interpretation/405 kyr-tuned astrochronologic framework). Is that a pure coincidence, or could there be a link here? OR: could it be the case that an alternative cyclostratigraphic interpretation is possible for this upper interval -> e.g. can you exclude the possibility that in this upper/younger part of the record you're looking in fact at a different expression of dominant Milanković cycles in the GR series (and not necessarily a change in sedimentation rate)? For example, if the large-amplitude swings with a thickness of 4-5 m that start to appear above 189 m are not ~405 kyr eccentricity but instead obliquity amplitude modulation cycles around ~1 Myr, that would mean sedimentation rate remained relatively constant. And in terms of the phase relationship analysis, this would mean that one is comparing apples with oranges (i.e., eccentricity vs eccentricity between d13C and GR in the lower part of the record, eccentricity vs obliquity amplitude in the upper part of the record). I guess the alternative obliquity interpretation has not been tested with eTimeOpt, which does not consider obliquity + its AM components.

I recommend to discuss this alternative possibility in the manuscript and/or explain why you think this is not a valid option, but then also explain the coincidence between an abrupt change in sedimentation rate and phase relationship.

Secondly: l. 382: "Mudstones with low calcium carbonate content were excluded." -> this is potentially problematic, since the interpreted eccentricity-scale fluctuations in GR signal are reflecting alternations in clay minerals.. See the controversy between Mitchell et al. 2008 and Lanci et al. 2010 about the orbital phase relationship of Cretaceous deep marine anoxia (whether they occurred during eccentricity maxima or minima). It turned out that Lanci et al. had arrived at an opposite phase relationship a the one proposed by Mitchell et al., 2008 because they had excluded the black cherts from the CaCO3 analysis, which are the main recorder of the astronomical cyclicity (Batenburg et al. 2012 EGU abstract). For the present manuscript, I understand the argument about the sufficient sampling resolution to capture short eccentricity-scale variability in d13C (resolution of ~25 kyr), but that does not exclude the possibility of bias arising from systematically not sampling a particular lithology that is in fact are key recorder of the astronomical-induced climate cyclicity. Or am I missing something here?

Minor points:

l. 93-94 "which is further substantiated by results derived from the ZB23-N64 astronomical solution (Zeebe and Lantink 2024b)" -> the fact that a disruption of the ~405 kyr eccentricity cycle does not occur in the 64 solutions of ZB23 for the late Carboniferous time interval is not an argument that it could not have happened. See p. 5 of Zeebe and Lantink 2024a: the estimated timescale for a possible σ12-resonance occurrence is of order 10^8–10^9 yr. Because so far only 64 solutions have been tested, the youngest possible age of a σ12-resonance interval that could be detectable in the geologic record is yet unknown. So just say that it did not occur in the 64 solutions that currently are available. Also: I would refer to the solutions as "ZB23" not "ZB23-N64".
l.116-121: The chaotic nature of secular planetary orbital motion within our solar system was shown decades ago based on astronomical (numerical) evidence (Laskar, 1989) and has been independently confirmed by geological evidence (Ma et al., 2017; Zeebe and Lourens, 2019; Olsen et al., 2019). Hence the phrase "were proposed to be chaotic" is not appropriate as it suggests this is still a topic of debate. In addition, the phrase "It likely supports the non-chaotic behavior of the solar system" is somewhat misleading, because you strictly can't speak of chaotic vs. non-chaotic behavior if the system is inherently chaotic/ not precisely predictable. So I would rephrase (and condense) this part by simply mentioning that the results of amplitude modulation analysis suggest that the periods of the very long eccentricity and obliquity amplitude modulation cycles were similar to those of the more recent past.

l .115: “long-term obliquity” -> long-period obliquity amplitude modulation cycle

l. 112-113 “ Modulation analysis..” -> Amplitude modulation analysis

l. 426 “ZB23-N64 solution” -> “ZB23”

Fig. 2ace: Power spectra are way too small; I can’t distinguish areas of increased spectral power from areas of lower spectral power (idem for Supplementary Fig. 2)

I recommend that the authors include Supplementary Fig. 9 in the main article, because it shows the inferred phase relation shift much more clearly than Fig. 3.

Supplementary Fig. 4: the translucent gray line (indicating the interpreted average sedimentation rate) is barely visible -> can’t you use a thick white line instead?

Check for spelling errors – e.g. “ampliutude” an “Costal onlap” in Supplementary Fig. 7.

References:

Batenburg, S. J., Montanari, A., Sprovieri, M., Hilgen, F. J., Coccioni, R., & Gale, A. S. (2012, April). Astronomical tuning of black cherts in the Cenomanian Scaglia Bianca as precursors of the Bonarelli level (OAE2) at Furlo, Italy. In EGU General Assembly Conference Abstracts (p. 6185).

Lanci, L., Muttoni, G., & Erba, E. (2010). Astronomical tuning of the Cenomanian Scaglia Bianca Formation at Furlo, Italy. Earth and Planetary Science Letters, 292(1-2), 231-237.

Mitchell, R. N., Bice, D. M., Montanari, A., Cleaveland, L. C., Christianson, K. T., Coccioni, R., & Hinnov, L. A. (2008). Oceanic anoxic cycles? Orbital prelude to the Bonarelli Level (OAE 2). Earth and Planetary Science Letters, 267(1-2), 1-16.

Reviewer #2

(Remarks to the Author)
The authors have addressed all critiques that I raised in my review, mostly to satisfaction. This must have taken quite some effort, and I commend them for their work. I think that some of the additions to the supplement warrant a mention in the main text, so that readers are aware of the extra analyses that were performed.

The resolution of figure 13/S2 is too low to be able to tell if the spectral analysis on the raw/undetrended record shows the same spectral peaks or not. The spectral peaks associated with long eccentricity at 4.5-2 cycles/m seem to fall below the 99% chi-squared confidence level that the authors use throughout the text, but it's hard to see.

The new paragraph that was added starting at L116 is confusing, however, and needs some revision. The solar system has long been known to be chaotic, see e.g., Laskar 1989, Peterson 1993, and many others. Hence, the first sentence needs to be revised. The next sentence needs a qualifier that periods *in the studied time interval* are indistinguishable from those of the past 36 Myr. The concluding sentence is also confusing: the chaos in the solar system is not invalidated, even though the particular feature of the resonance transition that in some solutions resulted in a weak 405 kyr cycle has not manifested in the solutions for this time interval. I would revise to something along the lines of “the long eccentricity cycle appears stable in all currently available numerical solutions for this time interval.” Furthermore, I appreciate that the authors performed spectral analysis on the available astronomical solutions for this time interval (ZB23-N64). Their spectral analysis figure shows that in some of the solutions, the spectral power of the long eccentricity cycle is much lower (about a quarter) than the others, indicating variability in the expression of the long eccentricity cycle.

In the response to another reviewer I saw that the authors wrote “Mudstones with low calcium carbonate content were excluded.” This sentence made me worry about mudstones appearing regularly, potentially excluding a part of the cycle. It is essential to explain where these mudstones that were excluded occurred in the record and how frequent they were to make sure that the cyclostratigraphic interpretation is not based on a partial signal.

L416: These analyses were conducted in the Acycle72. -> remove 'the' or add 'software'. Similar for L458--460. Software names are singular.

Version 2:

Reviewer comments:

Reviewer #1

(Remarks to the Author)
I have no further major comments – congratulations with this thought-provoking paper. (The suggested similarities between secular climatic trends of the late Paleozoic versus late Cenozoic are intriguing.) One recommendation for any follow-up study (?) could be to look in much higher resolution (i.e., resolving precession and obliquity-scale signals) at the key interval where the hypothesized transition in phase relation in the eccentricity band took place. Also, I would still be in favor of adding a zoom-in to Fig. 3 (or something) that shows the phase relation pattern in the raw data (such as in Fig. 1 of Turner, 2014)).

Minor suggestions:
l. 113: (*dark* blue arrow in Fig. 1e)
l. 206: “ices”?
l. 258: “We also discovered” -> “We also note/observe…”
l. 296-299: This sentence is a bit confusing to me. Do you mean that astronomical forcing played (only) a “passive” role i.e. by modulating/pacing climate change, or actively caused climate change by pushing the system beyond a critical threshold?

Reviewer #2

(Remarks to the Author)
The revised manuscript addresses most of the remaining critiques.

However, now that we have higher-resolution figures of the spectra, I cannot agree with this newly added sentence on line 90 and 91: "These cycles can be clearly observed in the raw data series and data after applying different ways of detrending (Supplementary Fig. 2)."

The revised fig S2 shows that ONLY the peak near 8.8 cycles/m as well as the lowest-frequency peak near 0.1 cycles/m cross the 5% FDR. The remainder of the peaks in the raw data do not cross the 5% FDR level. The indicated bands do cross the 99% chi-square CL.

Further down in the methods, you state:

415 A confidence level of ≥ 99% was utilized to validate the existence of significant
416 stratigraphic cycles, minimizing Type II (false negative) errors70. Astronomical
417 calibration can partially reduce disruptions caused by variation in sedimentation rate. To
418 avoid erroneously accepting a null hypothesis indicating no astronomical forcing, a false
419 discovery rate (FDR) threshold of ≤ 5% was established to detect robust signals of
420 astronomical cycles after astronomical calibration, avoiding Type I (false positive)
421 errors69.

So which is it, are you applying a 99% chi-square CL or a 5% FDR?

If the methods would say you choose to use the 99% level in favor of the 5% FDR and articulate why, it would be consistent. But you cannot say you apply both criteria and then not meet those of one of them.

Sorry for being pedantic about the statistics, but this subject has been a hot topic for debate in our field and having a paper published in nature with such confusing wording/inconsistency would only worsen the situation.

Open Access This Peer Review File is licensed under a Creative Commons Attribution 4.0 International License, which permits use, sharing, adaptation, distribution and reproduction in any medium or format, as long as you give appropriate credit to the original author(s) and the source, provide a link to the Creative Commons license, and indicate if changes were made.
In cases where reviewers are anonymous, credit should be given to 'Anonymous Referee' and the source.
The images or other third party material in this Peer Review File are included in the article's Creative Commons license, unless indicated otherwise in a credit line to the material. If material is not included in the article's Creative Commons license and your intended use is not permitted by statutory regulation or exceeds the permitted use, you will need to obtain permission directly from the copyright holder.
To view a copy of this license, visit https://creativecommons.org/licenses/by/4.0/

Revision notes:

Responses to reviewers:

We appreciate the reviewers' comments, leading to significant improvements of our manuscript. Below the reviewers' comments, and we provide our responses to reviewer comments in blue text.

Response to reviewer #1:

General comments:

In their manuscript (ms), Fang et al. present new gamma ray (GR) and $\delta^{13}C$ bulk-rock carbonate measurements of the upper Carboniferous Narao section (China) to investigate astronomical climate forcing and carbon cycle dynamics during the Late Paleozoic Ice Age. Using time-series analysis, the authors identify a shift from in-phase to anti-phase behavior between variations in GR (a proxy for glacio-eustatic sea level (SL) change) and $\delta^{13}C$ on eccentricity timescales around the Moscovian-Kasimovian boundary (~307 Ma), that is, from coupled low SL-high $\delta^{13}C$ to low SL-low $\delta^{13}C$ values. This shift is interpreted as a reorganization of Earth's climate-carbon cycle system in response to orbital forcing – from a growing terrestrial biosphere during glacial intervals to a shrinking terrestrial biosphere during glacial intervals – analogous to the shift from in-phase to antiphase behavior between benthic foraminifera $\delta^{18}O$ and $\delta^{13}C$ during the Late Cenozoic. **The proposed phase shift and analogy to the Late Cenozoic Ice Age is fascinating, if true**. **However, I see a couple of potential issues that need to be addressed first and/or require further clarification before I can recommend publication**.

Response: Thank you very much for your positive comments on our work. We have thoroughly considered all your suggestions and conducted further data analysis. The results of these analyses are presented below, and some have also been included in the supplementary material.

Major comments:

1. Insufficient info on $\delta^{13}C$. At the moment, I cannot properly assess the robustness of the proposed phase relation shift between GR and $\delta^{13}C$ on eccentricity timescales, because no results of cyclostratigraphic analysis (Milanković hypothesis testing) are presented for the Narao $\delta^{13}C$ record (at least as far as I am aware). If the goal is to investigate phase relationships between changes in global SL/ice volume and the carbon cycle in response to orbital forcing, one also needs to show (convincingly) that both components are indeed controlled by orbital forcing in the first place. In addition, the authors need to provide more information about the sampling strategy – in particular, how are $\delta^{13}C$ sample depths related to GR depths? Currently, the Methods section mentions that 5000 GR data points from the Narao section were taken "..with an average sample distance of 0.05 m", and a total of 1307 samples for $\delta^{13}C$ (lines. 473-477). In the Excel data file, however, I find $\delta^{13}C$ data interpolated also at 0.05 m steps, so no raw depths are given. What was the original sampling density of $\delta^{13}C$, and how is it related to GR? If, for example, $\delta^{13}C$ data are offset from GR by 1-2 m, this could artificially introduce a change from in-phase to anti-phase behavior. Also, I would recommend to include a figure in the ms that shows to readers a zoom-in of a

typical/well-expressed eccentricity cycle in GR and $\delta^{13}C$ from where these proxies are in phase, and one from where they are out of phase. (So in addition to the phase-relationship plot in Fig. 3j).

Response: Thank you for your valuable suggestions. In the revised manuscript, we address your concerns and suggestions as follows. First, to detect the robust evidence for cyclicity in the datasets, we identified the spectral background using a method named 'smoothed window averaging' (SWA) for red-noise spectra. This method represents an empirical, non-parametric procedure that does not assume the underlying cause of the sloping spectral background (Weedon et al., 2019; Weedon, 2022). Figure 1 (below) shows the results of the SWA background fitting for the GR and $\delta^{13}C$ spectra, including the standard Chi-squared 99% confidence level and the 5% false discovery rate (FDR) level. Evolutionary spectrograms are also shown. Spectral analysis of the 405-kyr-calibrated GR time series shows very distinct periods of the Milanković cycle, including the 405 kyr (long eccentricity), the 134.5 kyr (short eccentricity), 32.5–29.8 kyr (obliquity) and the 20.5 kyr, 18.6 kyr and 15.6 kyr (precession) periodicities. Because the average temporal resolution of the $\delta^{13}C$ data is ~25 kyr, we are not able to identify any cycles of < 50 kyr periodicity. Spectral analysis of the 405 kyr-calibrated $\delta^{13}C$ time series shows the 405 kyr and 131 kyr periodicities exceeding the 5% FDR level. Other periodicities related to short eccentricity are identified for 135.3 kyr, 125.6 kyr and 105 kyr, exceeding the Chi-squared 99% confidence level. These results indicate strong eccentricity forcing in the $\delta^{13}C$ series. We have added this figure to the main manuscript as 'Figure 2c, d, e, f'.

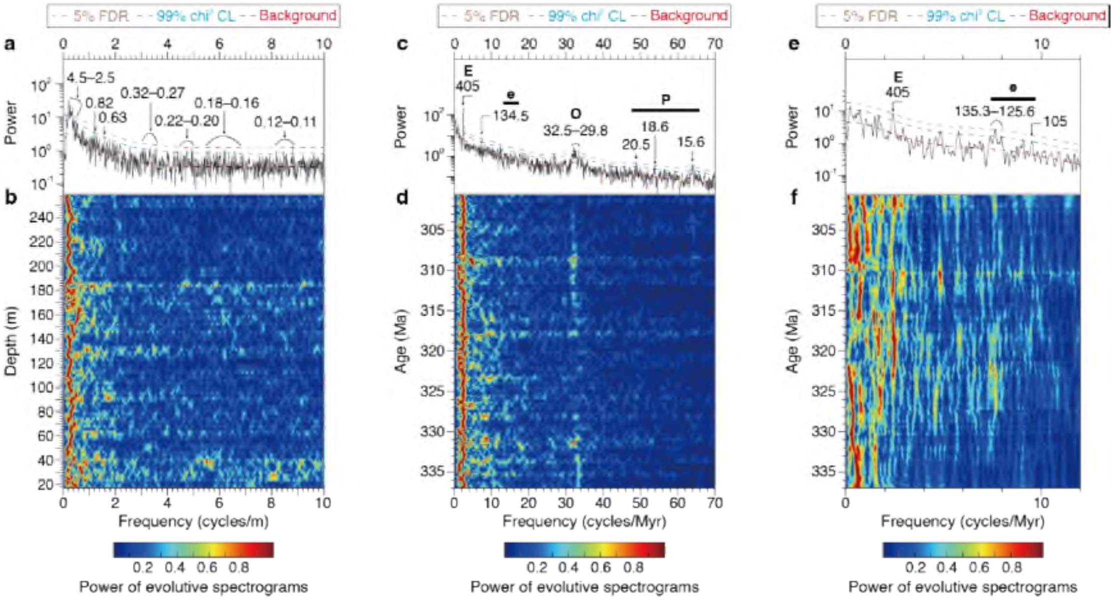

figure 1 Cyclostratigraphic analysis of the datasets from the Narao section.
a Smoothed window averaging (SWA) background fitting for the power spectrum of GR stratigraphic series, including the standard Chi-squared 99% confidence level (CL) and the 5% false discovery rate (FDR) level. **b** Evolutionary spectrogram of GR series (8-m-analysis window and 0.1-m steps). **c** SWA background fitting for the power spectrum of 405-kyr-calibrated GR series. **d** Evolutionary spectrogram of 405-kyr-calibrated GR series (1-Myr-analysis window width and 0.05-Myr steps). **e** SWA background fitting for the power spectrum of 405-kyr-

calibrated $\delta^{13}C$ series. **f** Evolutionary spectrogram of 405-kyr-calibrated $\delta^{13}C$ series (6-Myr-analysis window width and 0.2-Myr steps).

Second, to address concern about the sampling strategy, we refer to the Excel data file (sheet 'Figure 1c-f' of Source Data Fig. 1) provided with the original submission. In that file, we present two sets of $\delta^{13}C$ values: one for our newly measured $\delta^{13}C$ values for the Narao succession (1307 data points) and the other of published $\delta^{13}C$ data for the Narao succession (193 data points; Chen et al., 2016; Tian et al., 2019; Qi et al., 2021). The details were illustrated using different colours in Figure 1f of the main manuscript. We collected rock samples for C isotope analysis at 10-cm intervals calibrated to the horizons where we conducted GR measurements. Based on the established astronomical timescale, this indicates that the $\delta^{13}C_{carb}$ data correspond to ~25-kyr intervals (please see figure 2a below). The temporal resolution of the $\delta^{13}C_{carb}$ data points within the 'shifted phase relation' interval (e.g., 312–302 Ma) is ~14–25 kyr, which is sufficient to robustly evaluate the phase relationship of the ~100 kyr and 405 kyr eccentricity bands (please see figure 2b below). Also, considering the residence time of carbon in the oceans (10^4 yr), a higher temporal resolution for the $\delta^{13}C$ data may not produce a more robust result. We thus consider that the sampling resolution is sufficient to avoid signal aliasing of lower frequency periods and to detect eccentricity cycles. We added to the 'method' section the following statements regarding our sampling strategy: (1) '**Limestone samples were obtained at 10-cm intervals along the horizon where GR measurements were conducted. Based on the established astronomical timescale, the temporal resolution of the δ13Ccarb measurements is ~25-kyr. The Narao δ13Ccarb record was generated using samples of micritic limestone matrix drilled from fresh rock surfaces. Mudstones with low calcium carbonate content were excluded." (2) '1,307 samples from the Narao section were analyzed for $\delta^{13}C_{carb}$, and integrated with published $\delta^{13}C_{carb}$ data (n=193)[24,25,67], which were used for further study. Bulk $\delta^{13}C_{carb}$ from the adjacent Naqing succession was also conducted to further validate the findings obtained from the Narao succession. For the Naqing section, 475 samples were analyzed for $\delta^{13}C_{carb}$ and integrated with 245 published data points[24,67]. The $\delta^{13}C_{carb}$ time series were calibrated using a published astronomical timescale[37] for cyclostratigraphy analysis (Supplementary Fig. 10)."**

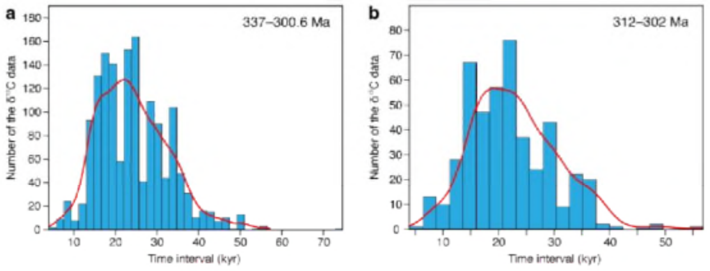

figure 2 The intervals of the $\delta^{13}C$ data points before and after astronomical calibration. **a** Distribution of the time-interval of the time-calibrated $\delta^{13}C$ data points throughout the Narao section. **b** Distribution of the time-interval of the time-calibrated $\delta^{13}C$ data points during the shifted phase relation period (312–302 Ma).

Thirdly, we have added a figure (Supplementary Fig. 9) in the supplementary material that provides readers with a detailed view of the phase shift in GR and $\delta^{13}C_{carb}$ on the long and short eccentricity bands. This is also shown in figure 3 below. The filtering outputs of long and short eccentricity from GR and $\delta^{13}C$ show that the final transition from in-phase to anti-phase relations occurred at ~307 Ma.

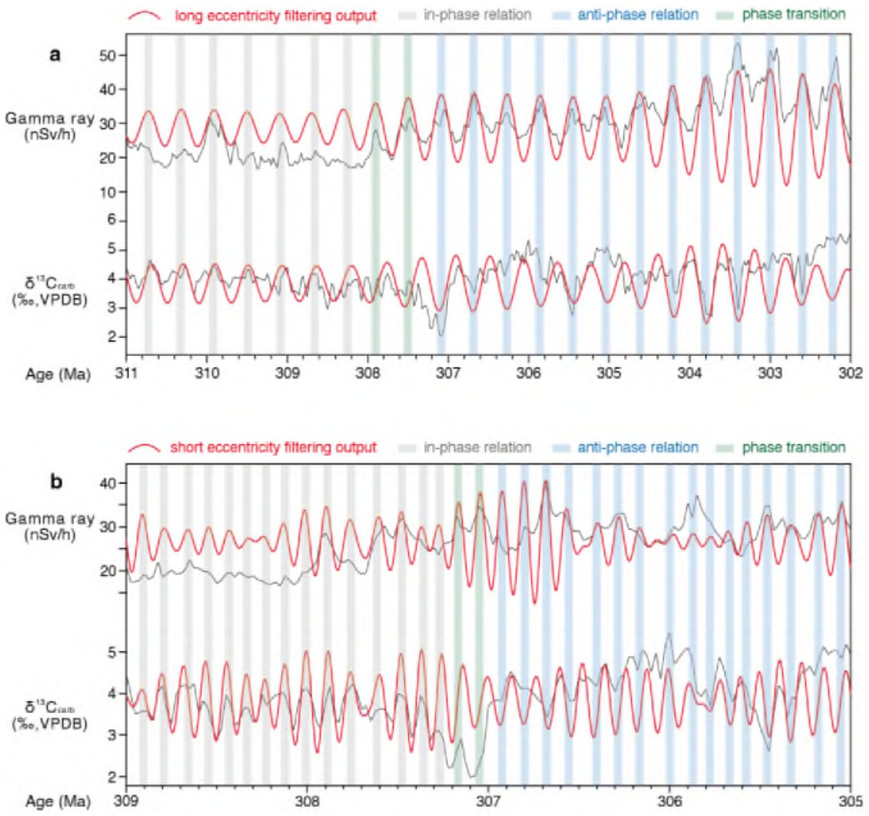

figure 3 Phase relation between GR and $\delta^{13}C_{carb}$ data from Narao section on short and long eccentricity bands.

a Phase relation between GR and $\delta^{13}C_{carb}$ data on the long eccentricity bands over the 311−302 Ma interval. 405-kyr signals (red curves) were filtered using a passband of 2.46 ± 0.3 Myr^{-1}. **b** Phase relation between GR and $\delta^{13}C_{carb}$ on the short eccentricity bands over the 309−305 Ma interval. Short eccentricity signals (red curves) were filtered using a passband of 8.5 ± 1.5 Myr^{-1}. Note that the in-phase behaviour inverts to anti-phase at ~307 Ma.

2. Uncertainty in the astronomical timescale –> this should definitely get more attention in the ms (currently uncertainty is not discussed at all). Specifically, what would be the consequences of errors in the astrochronologic framework for the inferred phase relationships? First, tuning was done based on the long (~405 kyr) eccentricity cycle, which the authors refer to as ".. a stable astronomical component throughout the geological history." However, see the paper by Zeebe and Lantink (2024), which shows that the long eccentricity cycle can become unstable on 10^8-10^9 year scales. Second, I find it very hard to believe that a complete record of Milanković forcing of ~37 Myrs long is preserved in a single section, especially considering the nature of the sedimentary environment ("deep-marine slope deposits", see l. 602). I think the longest

deep-sea (composite) record used in Westerhold et al. (2020) is about ~20 Myr. The Newark-Hartford record of Olsen et al (2019) is also on that order; astronomical signal quality and the density of independent chronostratigraphic constraints is much higher for these records than is the case for the Narao data (which has zero independent U-Pb constraints…). What about the possibility of turbidites or hiatuses associated with sea level change? In Extended Data Fig. 1c, I can clearly see irregular bedding above cycle E7 and E32. In Fig. 2, the EHA plot shows a disruption of the (inferred) long eccentricity-scale signal around 0-30 m and ~165-185 height (just before the inferred phase shift occurs).

Response: Thank you very much for the insightful comments.

First, sedimentological and geochronological evidence from Carboniferous and early Permian successions worldwide support the stability of the period of the 405-kyr-eccentricity over this interval of time. The "cyclothem" defined as 'a succession of lithologies, typically a few metres to tens of metres in thickness, recording alternation of nonmarine and marine depositional conditions, formed under low accommodation and low sediment supply, most widely though not exclusively in the palaeotropics of the late Palaeozoic" (Fielding, 2021). The Pennsylvanian cyclothems of North America have long been thought to represent 400-kyr-scale cycling (Heckel, 1986, 2008). High-precision CA-IDTIMS U-Pb dating of single zircons in volcanic ashes of the Carboniferous succession, Donets Basin, Ukraine, supports this timing, together with correlation of cyclothems of the Donets Basin succession with North American cyclothems (Heckel et al. 2007; Davydov et al., 2010; Schmitz and Davydov, 2012; Eros et al., 2012). We calculated the period of Milanković cycle in the ZB23-N64 solution (Zeebe and Lantink, 2024), and the result yields an average period of 405 kyr for eccentricity recorded in the Narao succession (Supplementary Fig. 3). However, it remains challenging to ascertain whether this eccentricity cycle precisely corresponds to a duration of exactly 405 kyr due to absence of robust astronomical solution. It is important to note that in the deep time, minor errors within several kiloyears may not significantly impact the results presented in our manuscript. We hold Zeebe and Lantink's (2024) work in high regard and consider the 'stability of the 405-kyr-eccentricity' as an ongoing topic for discussion. Thus, we have added some comments in the main text, as "**Although the stability of the 405-kyr eccentricity cycle in the deep time remains a topic of ongoing debate[28,29], geochronological evidence supports its relatively stable period during much of the Carboniferous[30-32], which is further substantiated by results derived from the ZB23-N64 astronomical solution[33] (Supplementary Fig. 3)**".

Second, the original statement referring to the Narao section as a deep marine environment is better referred to as an open-marine deep water succession. We revised this to '**The Viséan to Gzhelian sediments of the Narao section were deposited on an open marine deep-water (carbonate) slope[24,25] and are characterized by an average sedimentation rate of ~0.7 cm/kyr**'.

Third, the reliability of the Milanković signal at the Narao section was verified through TimeOpt and the spectral analysis of the 405-kyr-calibrated GR series. TimeOpt or Time Scale Optimization, emphasizes that the evaluation of time-scale uncertainty should be a central component of astrochronologic testing. At the core of the method is an assessment of two attributes that are diagnostic of an astronomical signal: (1) the concentration of spectral power at predicted astronomical frequencies (405 kyr, 132 kyr, 124 kyr, 100 kyr and 95 kyr in our study), and (2) the amplitude modulation of astronomical periods (long-eccentricity amplitude modulation of the short eccentricity band in our study). These two astronomical attributes are evaluated using a probabilistic regression model framework. The $r^2_{spectral}$ of our GR series is relatively high, but the $r^2_{envelope}$ of our GR series is relatively low. This may indicate that the Narao GR time series robustly records the frequencies of eccentricity, and records relatively well the amplitude modulation of eccentricity. In comparison, the Newark Basin record show different patterns in the TimeOpt results. The $r^2_{envelope}$ of lithological rank data is very high, but the $r^2_{spectral}$ is relatively low. The r^2_{opt} of both records, however, show near equal value (r^2_{opt}= ~0.2–0.3). These results can be interpreted as Milanković forcing is robustly recorded in both records. Moreover, after calibrating to the 405-kyr period, the Narao GR time series reveals the full Milanković cycle ensemble, including the 405 kyr (long eccentricity), 134.5 kyr (short eccentricity), 32.5–29.8 kyr (obliquity) and 20.5 kyr, 18.6 kyr and 15.6 kyr (precession) signals exceeding the 5% FDR level (> Chi-squared 99.9% confidence level). No other important period popped out after the astronomical calibration. We consider these results as the primary evidence to the Milanković signals in our record. We added some comment on it in main text, as "**Calibrating these cycles to the 405-kyr eccentricity period reveals dominant spectral peaks at 405 kyr (long eccentricity), 134.5 kyr (short eccentricity), 32.5–29.8 kyr (obliquity), and 20.5 kyr, 18.6 kyr and 15.6 kyr (precession; Fig. 2c, d), corresponding to average periods of the Carboniferous Milanković cycles (i.e., ~405 kyr, ~132 kyr, ~31.5 kyr, ~20.2 kyr, ~19.3 kyr and ~16.7 kyr)[33] (Supplementary Fig. 3).**".

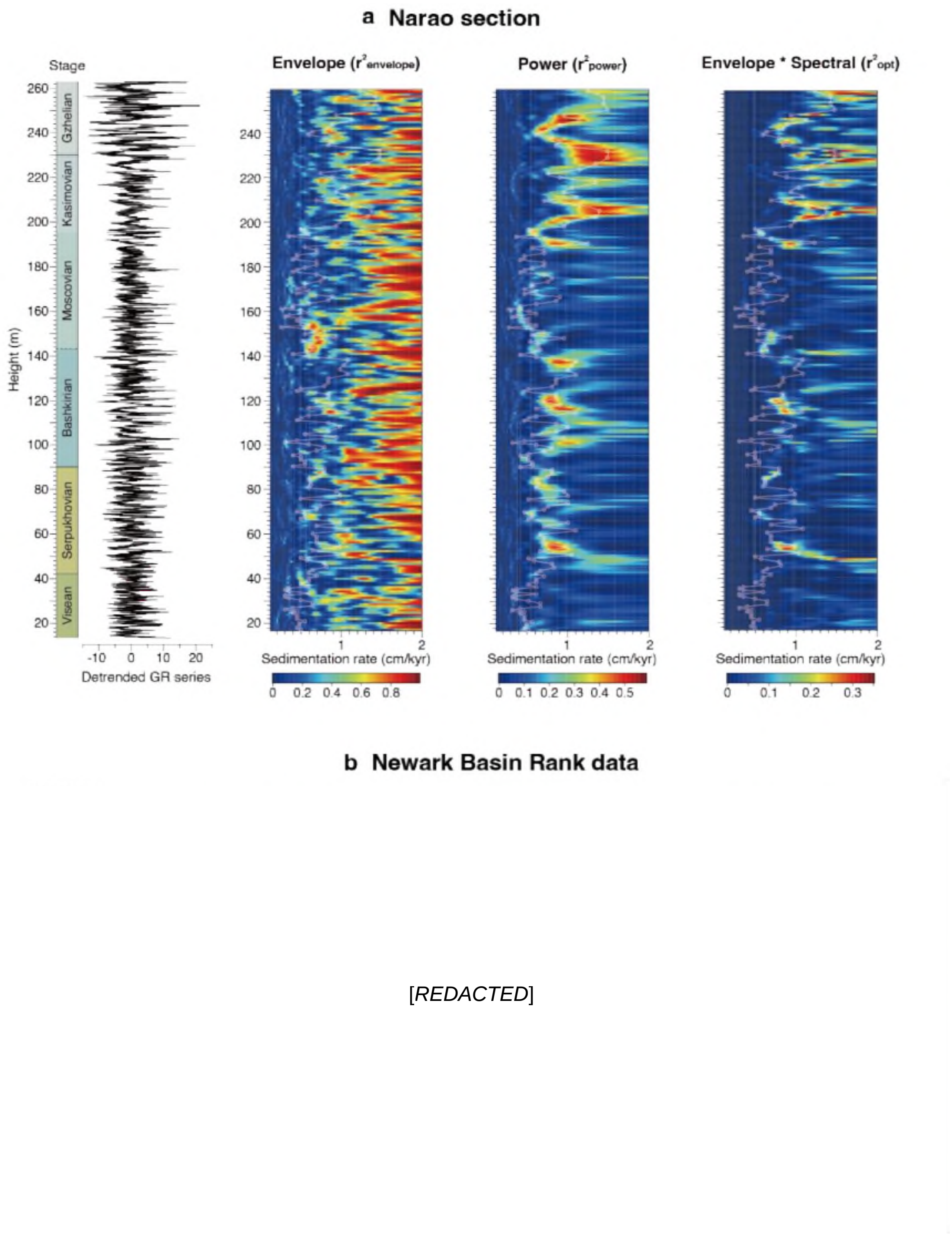

figure 4 Comparation of the eTimeopt results for the Narao (our study) and the Newark Basin successions (Meyers, 2019).

Fourth, the Narao section is characterized by thin-bedded lime mudstones intercalated with calciturbidites, a composition frequently observed in marine slope settings. The presence of turbidites may not significantly disrupt the Milanković signal; conversely, they may serve to preserve these signals. Examples of this phenomenon can be found in published studies (e.g., Weltje and de Boer, 1993; Heard et al., 2008; Payros and Martínez-Braceras, 2014). Narao sediments were predominantly deposited below the

photic zone, at water depths exceeding 200 m. Notably, glacio-eustatic sea-level variations did not result in significant hiatuses. To illustrate this point further, an extreme example can be drawn from the Mississippian/Pennsylvanian boundary (MPB), where widespread records indicate sea-level fall and associated hiatuses on a global scale (Saunders and Ramsbottom, 1986). The Global Stratotype Section and Point (GSSP) for the MPB located in Arrow Canyon, Nevada, is marked by several subaerial hiatuses represented by palaeosols or palaeokarst features that define multiple fourth-order glacio-eustatic cycles (Barnett and Wright, 2008; Lane et al., 1999; Bishop et al. 2009). Although a major glacio-eustatic fall, denoted by a slump deposit (see figure 5 below) is recorded at the MPB is in the Narao succession, stratigraphic and sedimentologic relationships/characteristics in the succession indicate that the carbonate slope remained submerged throughout its depositional history (with the exception of the MPB interval) and thus is considered a near-continuous succession (Chen et al. 2016; Tian et al., 2019).

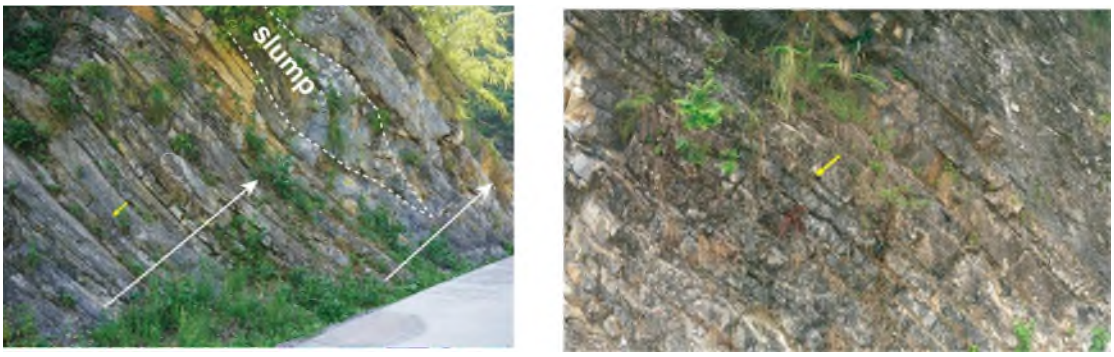

figure 5 Field photo showing the sampling trajectory and irregular bedding in Narao.

Fifth, the irregular bedding observed infrequently in the Narao section (yellow arrows in figure 5), may suggest the presence of small-scale hiatuses that likely to have minor impact on the overall completeness of the geological record given their uncommon existence in the succession. It is important to note that all stratigraphic sections inherently contain undetected hiatuses at various scales. The completeness of stratigraphic successions can only be meaningfully assessed at specific observational scales. For instance, a section representing a multi-million-year time span is likely to archive sediments representative of each 20-kyr interval; if this is indeed the case, it would be considered complete at the 20-kyr scale. However, this same section is considerably less likely to include sediment deposits from every hundred years. In other words, completeness at a century scale is substantially lower than that at a 20,000-year scale. The irregular bedding constitutes only a minor portion (2-3 cm) of thickness within one lithological bed and typically does not represent an extensive duration—especially when compared to the desired detection cycle of 20 kyr. Generally speaking, open-marine deepwater sections tend to exhibit greater completeness than continental shelf and terrestrial sections (Sadler, 1981). If present hiatuses are relatively minor in nature (as is the case for the Narao succession), then it is reasonable to consider the time series as complete at the scale of cycles of interest.

Six, a disruption of the inferred long eccentricity-scale signal is observed at depths of ~0–30 m and ~165–185 m. These disruptions were partially addressed through astronomical calibration. The geologic records from 13–30 m (337–333.3 Ma) and 165–185 m (312–308.6 Ma), and their power spectra are separately shown in figure 6a, b (below). The power associated with short eccentricity is relatively low compared to the strong obliquity forcing in these two intervals, which may be attributed to specific responses to astronomical forcing. The dominant climatic responses to astronomical forcing are well reflected in the Cenozoic world (please see Fig. 3 in the Westerhold et al., 2020; Science). However, how strongly a given orbital cycle is recorded through geologic time is influenced by various environmental factors. Modelled insolation-driven global temperature variability on the astronomical time scales suggests that different temperature-response regimes exist. Temperature response is dominated by eccentricity in the low latitudes, precession in the mid-latitudes, and by obliquity in the high latitudes (Laepple and Lohmann, 2009). For the LPIA, we propose that eccentricity cycles dominate the proxy time series in the early part of the record, indicating a strong influence of low-latitude processes on climate variations. With continued global cooling and ice sheet expansion, the obliquity-band response steadily increases (see figure 6c below). This hypothesis requires further research to be confirmed.

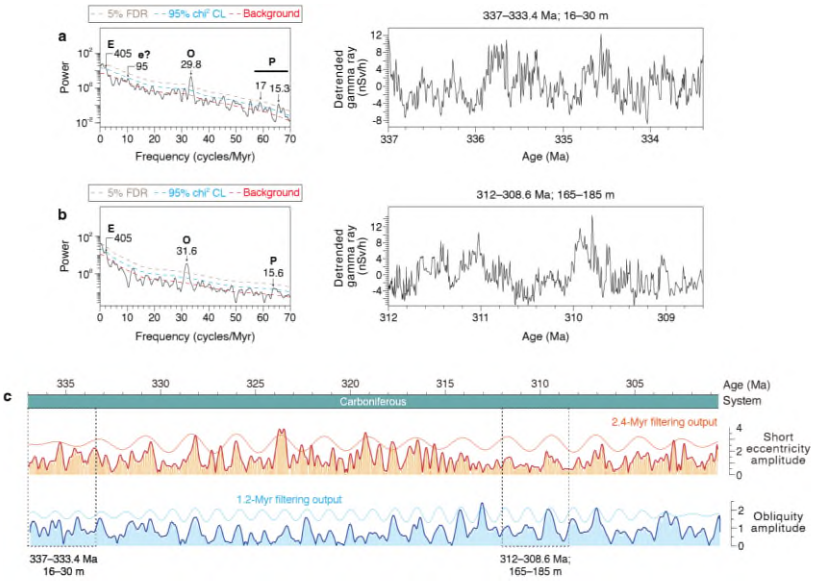

figure 6 Spectral analysis of the data at depths of ~13–30 m and ~165–185 m. **a** Spectral analysis of the astronomically-calibrated GR data (13–30 m) with the standard Chi-squared 95% confidence level and the 5% FDR level. **b** Spectral analysis of the astronomically-calibrated GR data (165–185 m) with the standard Chi-squared 95% confidence level and the 5% FDR level. **c** Short eccentricity and obliquity amplitude (power) throughout the section.

3. A global signal. It's a big leap from GR and $\delta^{13}C$ bulk-rock carbonate data of one section (Narao) to interpreting changes in the global climate-carbon cycle. The GR and $\delta^{13}C$ curves from the Narao section have been correlated to time-equivalent records from Donets and the GTS2020 site, indicating they may be representative for global-scale processes (Fig. 3i, Extended Data Fig. 7). However, the Donets and GTS datasets

are of a lower resolution and have not been analyzed at (and correlated on) Milanković timescales. So while the general trend in the Narao series may indeed reflect a global signal, I wouldn't dare to say that the correlation to Donets and GTS provides direct support for global-scale climate-carbon cycle processes on orbital timescales. (Cyclostratigraphic study has supposedly been conducted also one data from the lateral Naqing section – see l. 136-137 and Extended Data Fig. 8 - but the actual analyses are not shown in the current ms and a precise cyclostratigraphic correlation to Narao is also lacking).
Response: Thank you very much for your suggestion to improve our interpretations. Although the Donets succession has not been astronomically tuned (one of the authors Montañez have some discussion with Linda Hinnov and Steve Meyer about testing the potential to astronomically tune the Donets section, and awaits data retrieval for the Donets Basin), the bundling of cyclothems (242 of them) into larger-scale eccentricity scale cycles (i.e., the high-precision U-Pb ages for the Donets in some cases permit identification of the 100 kyr signal of cycles; see Davydov et al. 2010; Eros et al. 2012) — all calibrated by a dozen high-precision U-Pb zircon ages distributed throughout the succession, indicates that the Donets cyclic succession records Milankovitch cyclicity. Please refer to figure 7a below (the same one in Supplementary Figure 5a).

First, the Donets record was constrained using a dozen high-resolution ID-TIMS ages and detailed conodont biostratigraphy correlated to the North American midcontinent cyclothems (Heckel et al. 2007). The sea-level history is based on transgressions and regressions inferred from the stratigraphic facies. Although the resulting record is one of 'onlap and offlap' and not actually eustasy, evidence of the sea-level changes and approximate scale (m vs. 10s of m) is robust given that integrated subsurface and outcrop data permitted meter-scale correlation of the 242 biostratigraphically constrained cycles across ~250 km of the Donets Basin and characterization of their basinwide architecture. In turn, permitted the construction of an onlap–offlap history at a sub-400 kyr scale over the 30 Myr interval of the late Early and Late Carboniferous using the hierarchy of sequences and the geographic and stratigraphic positions of shifts in base-level sensitive facies across the Donets ramp. The Narao succession may have been more sensitive to sea-level changes given its open-water slope environment and thus is may be more suitable for accurately determining the age of the climate-carbon reorganization events. We conducted a stationary phase relation analysis using the onlap-offlap reconstruction from the Donets Basin and the $\delta^{13}C$ time series from the Narao section for the interval before and after 307 Ma. The results indicate that prior to 307 Ma, $\delta^{13}C$ maxima were associated with eccentricity-driven glacial-eustatic sea-level falls (see figure 7b below); conversely, after 307 Ma, $\delta^{13}C$ minima corresponded to eccentricity-driven sea-level falls (see figure 7c below). These findings strongly support our argument. Additionally, we have included these results in the main text, as "**The shifted phase relation can also be observed via the stationary phase relation analysis between $\delta^{13}C_{carb}$ from Narao and onlap–offlap record from the Donets Basin (Supplementary Fig. 7f, g), likely reflecting a global signal for the reorganization of climate-carbon cycle system on eccentricity time scale.**"

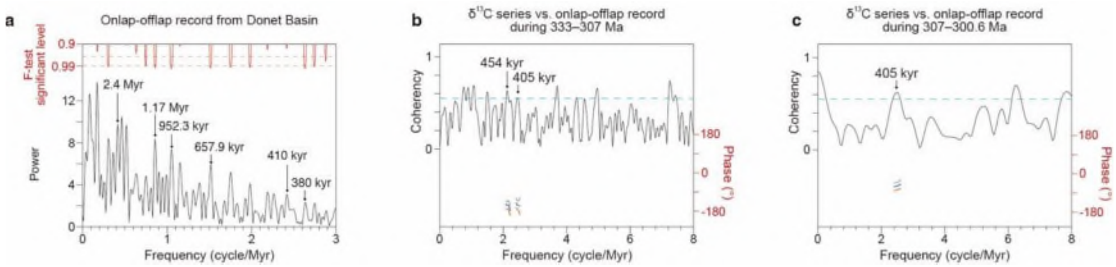

figure 7 Coherency and cross-phase spectral analysis between $\delta^{13}C$ time series from the Donets Basin and Narao succession

a Periodogram (black curve) and F-test values (red curve) of the costal onlap-offlap record from Donets Basin during 333–300.6 Ma. **b** Astronomically-calibrated $\delta^{13}C$ series versus onlap-offlap record during 333–307 Ma. **c** Astronomically-calibrated $\delta^{13}C$ series versus onlap-offlap record during the 307–300.6 Ma interval.

Second, as you suggested, we have provided additional details regarding the analysis of the Naqing section. This includes a cyclostratigraphic correlation between the Narao and Naqing sections (please see figure 8b, c below), spectral analyses of magnetic susceptibility and $\delta^{13}C$ data—which reveal distinct Milanković cycles (figure 8f below)—as well as phase analyses between the magnetic susceptibility and the $\delta^{13}C_{carb}$ data from the Naqing section (figure 8e below). These results further substantiate our argument. Additionally, we have included this information in the supplementary materials (Supplementary Fig. 10).

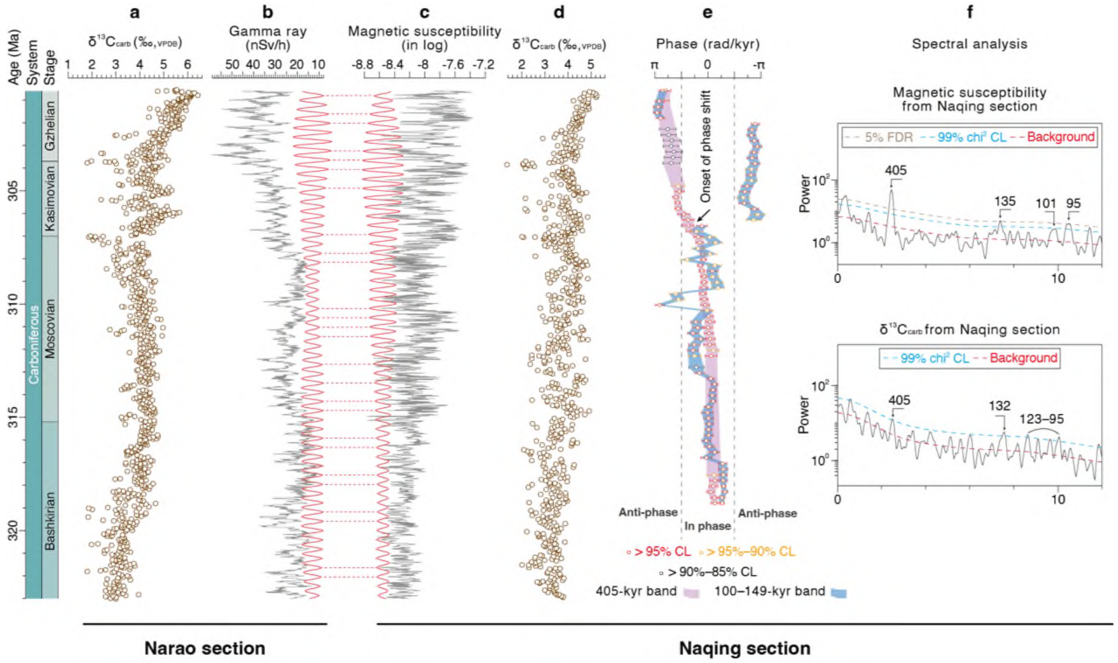

figure 8 Climate-carbon cycle relationships on the eccentricity timescales over the 322.8–300.6 Ma interval, recorded in the Naqing succession.

a $\delta^{13}C_{carb}$ time series from the Narao section. **b** GR time series from the Narao section. **c** 405-kyr-calibrated magnetic susceptibility time series for the Naqing section[24]. All 405-kyr eccentricity cycles (red curves) were extracted using a 2.46 ± 0.2 Myr^{-1} bandpass. **d** 405-kyr-calibrated $\delta^{13}C_{carb}$ time series from the Naqing section (see "Methods" section). **e** Evolution of phase-

relationship between magnetic susceptibility and $\delta^{13}C_{carb}$ on the 405-kyr (purple area) and 95–140-kyr (blue area) eccentricity time scales, with 4-Myr-width windows and 0.25-Myr steps. An abrupt shift from in-phase to anti-phase relationship is observed at ~307 Ma (proximal to the Moscovian-Kasimovian boundary). **f** Smoothed window averaging (SWA) background fitting for the power spectrum of MS and $\delta^{13}C_{carb}$, Naqing section, including the standard Chi-squared 99% confidence level (CL) and the 5% false discovery rate (FDR) level.

The uniqueness of our work is also reflected in the significance of the study sections. The $\delta^{13}C$ time series for the GTS was derived from the Arrow Canyon succession, Nevada. A divergence between the North American and European $\delta^{13}C$ records across the Mississippian-Pennsylvanian transition has been interpreted as the influence on oceanic circulation with closure of a subequatorial oceanic gateway during assembly of Pangaea (Saltzman, 2003). Notably, the slope and basinal environments of the South China microcontinent were well connected to the open ocean throughout Pennsylvanian through early Permian (Cisuralian). Thus, the $\delta^{13}C_{carb}$ values likely record a global seawater signal, as posited by several studies (Buggisch et al., 2011; Chen et al., 2018, 2022; Yang et al., 2023). On the basis of all of the aforementioned characteristics, we propose that there are no other high-resolution $\delta^{13}C$ records as well suited for analysis on the eccentricity scale during the LPIA than those of the Naqing and Narao sections presented here. —It is widely acknowledged that asserting any deep-time research as entirely 'global' poses significant challenges. For the LPIA, our research serves as an essential starting point; further integration of high-resolution data with these records will enhance these deep-time records.

Minor comments:

Line 42-43: “low-latitude continental carbon reservoirs expanded mostly during the astronomically-forced cool intervals (4) -> Is the reference to Kirkland-Turner (2014) appropriate here? Because from my understanding, that paper talks about a “gradual expansion of vegetated terrestrial biosphere during warm intervals”.

Response: We have changed the reference at your suggestion.

Line 65 “to test this hypothesis” -> elaborate.

Response: We have changed this sentence into “**At 307 Ma, arborescent lycopsids abruptly disappeared from Euramerican biomes[17,18], coincident with the onset of long-term tropical aridification[18], remaining only in Cathaysia (China)[20,21] that hold everwet condition through the late Paleozoic[19]. The continental-scale aridification likely intensified the repeated vegetational turnovers that characterized the remainder of the LPIA[12]. Thus, it is possible that the widespread loss of tropical ‘coal’ forests may have led to a change in the response of the global carbon cycle to astronomical forcing. This hypothesis, however, remains untested.**”

Line 74-75: how is it possible that no significant short eccentricity-scale peaks are identified in the raw depth series, only after tuning to a 405 kyr cycle?

Response: Thank you very much for pointing this out. We have changed the method for spectral analysis from the MTM spectrum with a background estimation using an AR-1 model to a SWA method with standard Chi-squared 99% confidence level. The

short eccentricity-scale peaks (0.82 and 0.63 m) are identified in the series (figure 1a above). Furthermore, the weak signal of short eccentricity is also partially associated with its varying periods, this could be seen after the astronomical calibration (figure 1d).

Line 83-86: “The calculated change in sedimentation rate…tracks with those obtained from two objective statistical methodologies, i.e.,…. and evolutionaly correlation coefficient.” I’ve attached a document to this review from prof. Stephen Meyers (personal comm.). In this document Meyers explains why he thinks that the eCOCO method of Li et al. (2018) is unfortunately flawed. Meyers has given me permission to share this document with the authors of the present ms. I haven’t discussed the current ms (Fang et al.) with Meyers.

Response: Thank you very much for sharing this information. As you and many researchers have indicated, (e)COCO may be flawed. Thus, we kept only the eTimeOpt result in the revised version.

Line 93-96: the ~1.2 and ~2.4 Myr Grand cycles are not stable in deep time.

Response: Thank you very much for your suggestion. We have changed into “**Even though the orbits of planets in the Solar System were proposed to be chaotic at timescales of 10–100 Myr[33], the periods of million-year-scale astronomical cycles are indistinguishable from those obtained by the astronomical solution (e.g., ZB18a astronomical solution) from the past 36 Ma[36] (Supplementary Fig. 6). It likely supports the non-chaotic behavior of the solar system during the main phase of the penultimate icehouse.**”

Line 108-111: I don’t understand how the long-term obliquity AM cycle can show an anti-phase relation with the Narao GR series and in-phase relation with the onlap-offlap record of the Donets, if Donets and Narao GR record display a constant antiphase relationship (l. 105-106).

Response: We show these phase relations in detail in figure 9 below. The minima of the obliquity AM cycle corresponds to the sea-level low, which was also indicated by higher GR values. Thus, the long-term obliquity AM cycle shows an anti-phase relation with the Narao GR for the 1.2-Myr band (see figure 9a below). Also, the minima of the obliquity AM cycle corresponds to the sea-level low, which was also indicated by lower values in the onlap-offlap record. Thus, the long-term obliquity AM cycle can show an in-phase relation with the onlap-offlap record of the Donets.

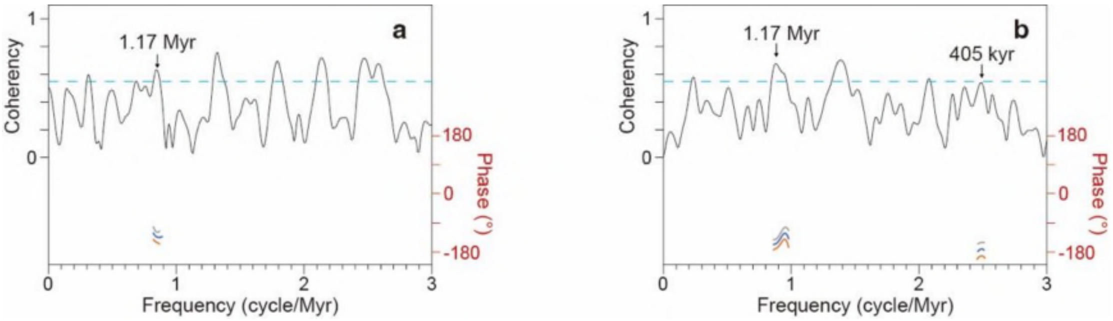

figure 9 Phase relationships between the Narao GR time series, obliquity amplitude modulation and onlap-offlap record from the Donets Basin.

a Astronomically-calibrated GR series vs. the amplitude modulation of the ~31-kyr obliquity cycle. **b** Astronomically-calibrated GR time series vs. the onlap-offlap record from the Donets Basin.

Line 134-135: "This shifted phase relation is delayed by ~0.5 Myr for the 95-132 kyr eccentricity band.." -> in Extended Data Fig. 2, I see no concentration of spectral lower in the short eccentricity range for Kasimovian-Gzehlian. Are the result of phase analysis then reliable for this interval?

Response: We appreciate the reviewer raising this issue. The signal of short eccentricity is robust (figure 6c above). The phase changes of the short eccentricity band can be seen in figure 3 above.

Response to reviewer #2:

General comments:

1 Review

This study presents an impressive record of novel bulk carbonate $\delta^{13}C$ measurements, with a new astronomical time scale based on gamma-ray data. The authors argue that the Late Paleozoic Ice Age shows interesting parallels to our current glacial world, and that this record can be used to test this hypothesis. **I think the work is significant and warrants publication.** However, there are some major issues with the astronomical solutions and data analysis that should be addressed, requiring major revisions. See attached PDF for the Major and Minor comments. I have also included a quick reproduction of the spectral analysis.

Major comments:

2.1 Astronomical Solutions

It is problematic to make a comparison between astronomical forcing at ~300.6–337.0 Ma to astronomical forcing for the past 35 Myr without mentioning this much more explicitly than just "in the Cenozoic Era" (lines 95 and 96). The inherent chaos in the solar system makes it impossible to identify a single astronomical solution for this time interval based on physical models alone. This means that it is highly unlikely that the astronomical forcing at around 300 Ma was similar enough to 0–34 Ma to make a direct comparison.

It is surprising to see the authors choose to use astronomical solution La10d because up to the Cretaceous–Paleogene boundary, solutions ZB18a, ZB20x, and to a lesser extent La10a, La10b, and La10c are most compatible with data (Zeebe & Lourens, 2022, EPSL). The La10x orbital solutions do not include the obliquity and climatic precession components, but only the eccentricity. How did the authors get these? (i.e. L93: "long-term obliquity" and L641: "Raw obliquity signal from La2010d nominal solution24"). Assuming that Acycle was used, it would be based on the methodology presented in Wu et al., (2013), who are not cited here. Furthermore, this approach is problematic because it applies a Fortran script from Laskar et al. (1993) that assumes that input is given the ecliptic reference frame to the La10x solutions, which are given in the invariant reference frame, leading to incorrect results for the obliquity.

The authors also use the La04 solution for determining the target periods for the eCOCO routine. The La04 solution eccentricity is already invalid for time periods prior to ~40 Myr (e.g., Laskar 2011). The obliquity and climatic precession estimates have even more uncertainty and should therefore not be used for a time period of ~300 Ma. There is a recent ensemble of 64 astronomical solutions available that cover this time period (ZB23-N64, between 0 and -3.5 Gyr). These solutions do include the climatic precession and obliquity (Zeebe and Lantink, 2024, Astronomical Journal, https://doi.org/10.3847/1538-3881/ad32cf. Solutions available on https://www.soest.hawaii.edu/oceanography/faculty/ zeebe_files/Astro.html).

Response: Thank you very much for the suggestion that significantly improved our manuscript. As to the astronomical solutions for eccentricity and obliquity (36–0 Ma), we have changed them into solution ZB18a which may be the most compatible with

geological data (Zeebe and Lourens, 2022). Please see figure 10 (below) and we also added this figure in the Supplementary materials (Supplementary Fig. 6). We added a comment to the revised manuscript: "**Even though the orbits of planets in the Solar System were proposed to be chaotic at timescales of 10–100 Myr[33], the periods of million-year-scale astronomical cycles are indistinguishable from those obtained by the astronomical solution (e.g., ZB18a astronomical solution) from the past 36 Ma[36] (Supplementary Fig. 6). It likely supports the non-chaotic behavior of the solar system during the main phase of the penultimate icehouse.**"

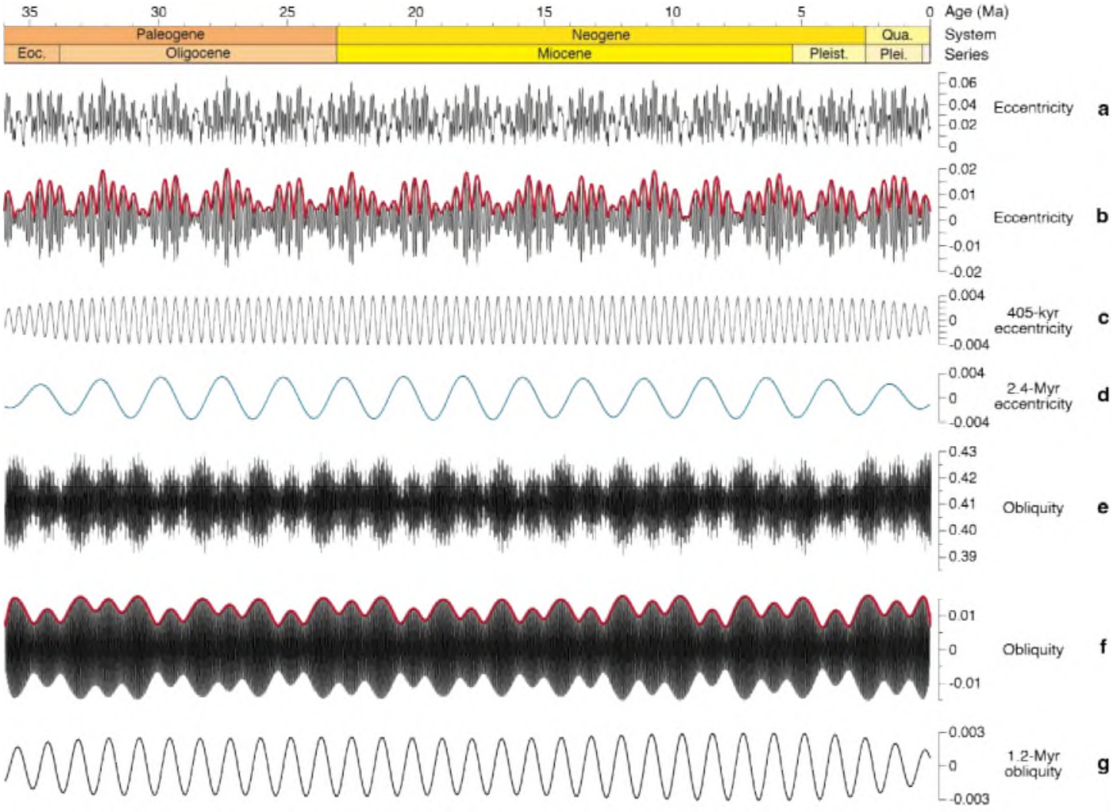

figure 10 The solution of Earth's astronomical parameters (ZB18a) for the past 36 Myr. **a** Raw eccentricity signal from ZB18a solution[19]. **b** Amplitude modulation (red curve) of ~100-kyr short eccentricity band (black curve), extracted using a bandpass of 9 ± 2 Myr^{-1}. **c** 405-kyr eccentricity cycle signal, extracted using a bandpass of 2.46 ± 0.1 Myr^{-1} from the amplitude modulation of the ~100-kyr short eccentricity cycle. **d** 2.4-Myr eccentricity cycle signal, extracted using a bandpass of 2.46 ± 0.1 Myr^{-1} from the amplitude modulation of the ~100-kyr short eccentricity cycle. **e** Raw obliquity signal from ZB18a solution. **f** Amplitude modulation (red curve) of the ~40-kyr obliquity band (black curve), extracted using a bandpass of 25 ± 1 Myr^{-1}. g 1.2-Myr obliquity cycle signal, extracted using a bandpass of 0.85 ± 0.1 Myr^{-1} from the amplitude modulation of the ~40-kyr obliquity cycle.

As for the target periods of the astronomical cycles in the Carboniferous, it is true that the obliquity and precession estimates have even more uncertainty and should therefore, not be used for the pre-Mesozoic era. Based on your suggestion, we calculated the periods for eccentricity, obliquity and climatic precession during the 337–300 Ma interval, using the ZB23-N64 solution (Zeebe and Lantink, 2024). The result is shown

in figure 11 below. Eccentricity periods include the ~405 kyr, ~132 kyr, ~124 kyr, ~100 kyr and ~95 kyr periodicities; obliquity periods include the 38.9 kyr, 31.5 kyr and 24.2 kyr periodicities; climatic precession periods include the 20.2 kyr, 19.3 kyr and 16.7 kyr periodicities. These periods of the astronomical cycles are totally consistent with the geological evidence in our study, which consists of the 405 kyr (long eccentricity), 134.5 kyr (short eccentricity), 32.5–29.8 kyr (obliquity) and 20.5 kyr, 18.6 kyr and 15.6 kyr signals (precession). We have addressed this in the revised manuscript, as "**Calibrating these cycles to the 405-kyr eccentricity period reveals dominant spectral peaks at 405 kyr (long eccentricity), 134.5 kyr (short eccentricity), 32.5–29.8 kyr (obliquity), and 20.5 kyr, 18.6 kyr and 15.6 kyr (precession; Fig. 2c, d), corresponding to average periods of the Carboniferous Milanković cycles (i.e., ~405 kyr, ~132 kyr, ~31.5 kyr, ~20.2 kyr, ~19.3 kyr and ~16.7 kyr)[33] (Supplementary Fig. 3).**" We also added the following figure into the Supplementary materials (Supplementary Fig. 3).

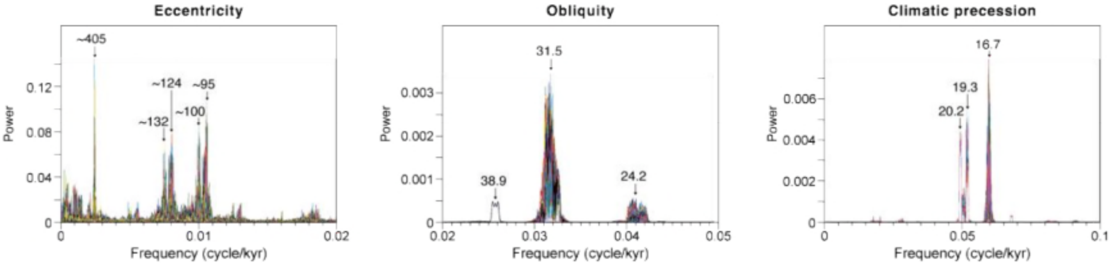

figure 11 Periods of eccentricity, obliquity and precession for the time interval of 337–300 Ma. There are 64 solutions using the ZB23-N64 solution (Zeebe and Lantink, 2024) used here to calculate the periods of eccentricity, obliquity and climatic precession cycles. Numbers are kiloyears.

Furthermore, it was recently demonstrated that the long eccentricity cycle (the "405 kyr cycle") is not stable over long time periods as previously thought (Zeebe & Lantink 2024, Astronomical Journal). In the ensemble of simulations, most LEC cycles appear stable over the past 300 Myr, but the period starts to vary and some solutions diverge significantly after several hundred million years. This needs to be addressed, because the authors rely on this assumption to anchor their 4.5–2.5 m cycles to a regular 405 kyr cycle (perhaps it is also relevant to SI L17: "However, the uppermost Serpukhovian and Bashkirian cyclothems70 fail to conform to the 405-kyr pattern18.").
Response: Thank you for your comments. We respect Zeebe and Lantink (2024)'s work and regard the 'stability of the 405-kyr-eccentricity' as an open discussion. Thus, we have addressed this in to the main manuscript as "**Although the stability of the 405-kyr eccentricity cycle in the deep time remains a topic of ongoing debate[28,29], geochronological evidence supports its relatively stable period during much of the Carboniferous[30-32], which is further substantiated by results derived from the ZB23-N64 astronomical solution[33] (Supplementary Fig. 3)**"

With regard to the uppermost Serpukhovian and Bashkirian cyclothems that do not conform to the 405-kyr pattern', we propose that this may reflect stratigraphic hiatuses

driven by a major eustatic fall, at the Mississippian/Pennsylvanian boundary (MPB) widely recorded in Euramerican carbonate platform successions. For instance, the GSSP section for the MPB in Arrow Canyon, Nevada, is punctuated by several subaerial hiatuses defining several fourth-order glacio-eustatic cycles (Barnett and Wright, 2008; Lane et al., 1999; Bishop et al. 2009). In the nearby Marble Canyon succession, the mid-Carboniferous lowstand is recognized as parasequences capped by exposure features despite the outermost platform to upper slope setting (Martin et al., 2012). In the Appalachian Basin, stacked palaeo-valleys are developed across the MPB (Blake and Beuthin, 2008). We have added this comment in the "Supplementary Text 1. Carboniferous astrochronology".

2.2 Spectral analysis

The authors make use of an MTM spectrum and plot a background estimation using an AR-1 model as well as the 95% significance level. They refer to the spectral peaks that cross this 95% CL as "significant spectral peaks", implying statistical null hypothesis significance testing. Previous studies (Weedon 2022, Earth-Science Reviews https://doi.org/10.1016/ j.earscirev.2022.104261; Smith 2023 P3 https://doi.org/10.1016/j.palaeo.2023.111744) have indicated problems with this application of spectral peak identification, and it appears from the extended data figure 2 that the authors are aware of this because they also present their analysis using an alternative SWA method with 5% false discovery rates.

However, they do not comment on why they present this alternative analysis or which they prefer, and the main manuscript figure still only uses MTM and refers to spectral peaks that stick out above the offset spectral background estimation as "significant". I realize that this debate is not settled yet and there are as yet no recommended "best practices" that everyone agrees on in the field of cyclostratigraphy, but I believe some mention of this is warranted.

Response: Thank you very much for your comment. As the reviewer appreciates the scientific community acknowledges these issues (e.g., Vaughan et al., 2011, 2015). The modeling results of Vaughan et al. (2011) demonstrate that confidence levels of <99% are unlikely to balance the respective probabilities of type I (false positive) and type II (false negative) errors, but instead lead to high probability of type I errors. Kemp (2016) advocated that confidence levels of 99–99.9% should be used to validate astronomical forcing in strata. Weedon (2018, 2022) suggested that Type I errors are more detrimental to rigorous studies than Type II errors in the cyclostratigraphic analysis and proposed using an alternative SWA method with 5% false discovery rates (likely corresponding to 99.9899% CL) to find true cyclicity. Nevertheless, using critical confidence levels of >99.9% risks a high likelihood of type II errors, and sedimentation rate variation sets an upper limit on the attainable significance of spectral peaks (Hinnov et al., 2016; Kemp, 2016). Thus, the 99% confidence level is advocated here to find the stratigraphic cycles, so as not to reject a null hypothesis of no astronomical forcing. Astronomical calibration can partially reduce the adverse effect of sedimentation rate variations. The significance of astronomical cyclicity is also enhanced after astronomical calibration. Thus, the 5% FDR we used in this study to find the cycles after the astronomical

calibration was chosen so as to not falsely accept a null hypothesis of no astronomical forcing. We have addressed this in the 'Methods' section, as "**It is essential to identify regular cyclicity in successions that lack high-resolution dating. We employed 'smoothed window averaging' (SWA)[69] to determine the spectral background. The SWA for red-noise spectra represents an empirical, non-parametric approach that makes no assumption about the underlying cause of the sloping spectral background[69]. The final results of the SWA background fitting for the time series power spectrum include the standard Chi-squared 99% confidence level and the 5% false discovery rate (FDR) level[69]. Variations in sediment accumulation rates can shift frequencies within a cyclostratigraphic series, allowing a significant cycle to be recorded across multiple frequencies at a fraction of its potential power if accumulation rates were constant. A confidence level of ≥ 99% was utilized to validate the existence of significant stratigraphic cycles, minimizing Type II (false negative) errors[70]. Astronomical calibration can partially reduce disruptions caused by variation in sedimentation rate. To avoid erroneously accepting a null hypothesis indicating no astronomical forcing, a false discovery rate (FDR) threshold of ≤ 5% was established to detect robust signals of astronomical cycles after astronomical calibration, avoiding Type I (false positive) errors[69]. Sliding-window spectral analysis was conducted using evolutive Fast Fourier Transform spectrograms (eFFT)[71]. These analyses were conducted in the Acycle[72].**" Please see the newly revised Figure 2 in the main manuscript, and we also show it as figure 12 below.

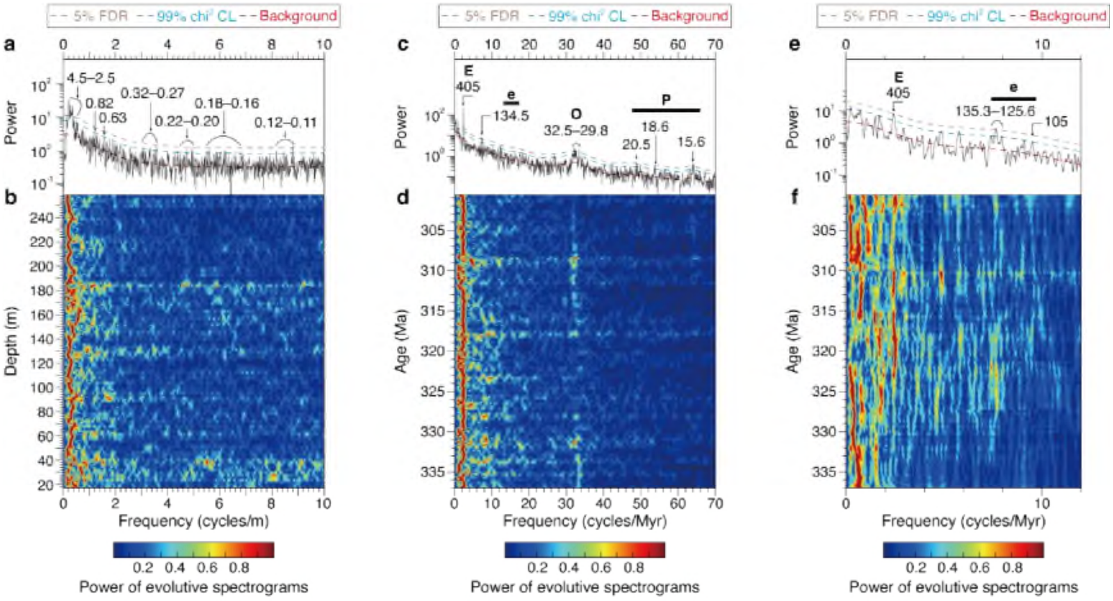

figure 12 Cyclostratigraphic analysis of the datasets from the Narao section.

a Smoothed Window Averaging (SWA) background fitting for the power spectrum of the GR stratigraphic time series, including the standard Chi-squared 99% confidence level (CL) and the 5 % false discovery rate (FDR) level. b Evolutionary spectrogram of the GR time series (sliding window = 8 m; step = 0.1 m). c SWA background fitting for the power spectrum of the 405-kyr-calibrated GR time series. d Evolutionary spectrogram of the 405-kyr-calibrated GR time series (sliding window = 1 Myr; step = 50 kyr). e SWA background fitting for the power spectrum of the

405-kyr-calibrated $\delta^{13}C$ time series. f Evolutionary spectrogram of the 405-kyr-calibrated $\delta^{13}C$ time series (sliding window = 6 Myr; step = 200 kyr).

L75: is this 8 m detrending also done prior to spectral analysis? The effects of first removing the 8-m trend on the spectral analysis are not discussed. See attached "Analysis" section for an illustration of how detrending the data prior to spectral analysis hardly affects the spectrum, but shifts the spectral background estimation of the AR-1 process down a lot, leading to more significant peaks. Using eTimeOpt requires inputting frequencies for the long and short eccentricity bands, but there are very few constraints on the periods of these cycles for this time interval. From L512 it is unclear what these periods are based on.

Then, for the description of the eCOCO routine at L535 the authors describe that they arrive at these periods by extrapolating from the La04 solution's 249 Ma. As described above, the La04 solution eccentricity is already invalid for time periods prior to ~40 Myr already (Laskar 2011). The obliquity and climatic precession estimates have even more uncertainty and should therefore not be used for a time period of ~300 Ma.

Response: Thank you very much for the comments. It is so true that the shift of the spectral background estimation of the AR-1 process leads to more significant peaks. We have changed the method for the spectral background estimation. We applied SWA on the results of deleting different LOWESS trends for red-noise spectra. Very stable frequencies were shown in the outputs. See figure 13 below. We added this figure into the Supplementary Materials (Supplementary Fig. S2). With regard to the periods of the long and short eccentricity cycles, we used ~405 kyr, ~132 kyr, ~124 kyr, ~100 kyr and ~95 kyr as the targets to conduct eTimeopt. These periods are calculated from the ZB23-N64 solution (Zeebe and Lantink, 2024). Please see figure 11 above.

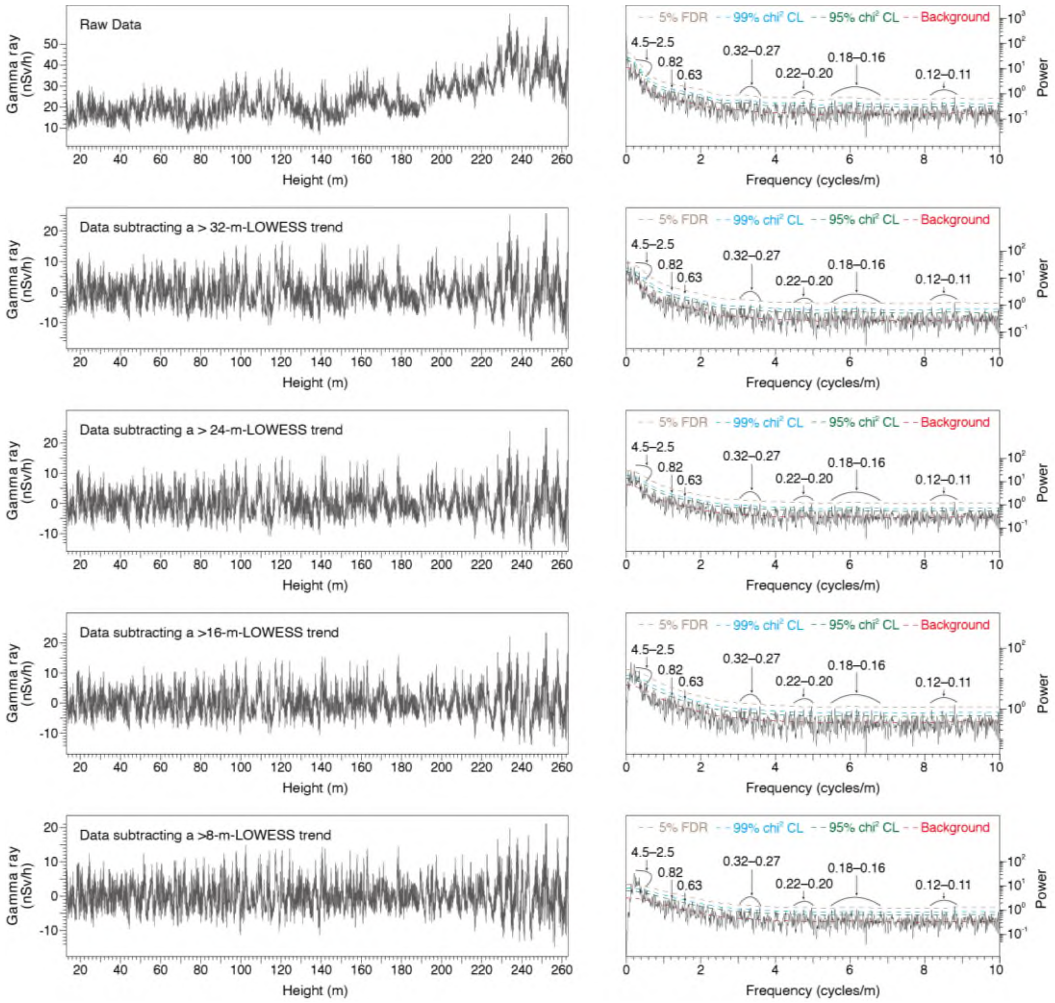

figure 13 Different detrending processes and their impact on the spectral analysis. Raw data and data subtracting a >32-m, >24-m, >16-m >8-m LOESS trend (left part), and their smoothed window averaging (SWA) background fitting for the power spectrum (right part), including the standard Chi-squared 99% and 95% confidence levels (CLs), and the 5% false discovery rate (FDR) level.

Extended data fig 2: "FDR at 5% is equivalent to the conventional 95% CL" This is factually incorrect. The false discovery rate is:

FDR = (*N* reject null is true)/ (*N* reject null is true + *N* reject null is false)

It is way more strict than the 95% CL. According to Weedon (2018, Geological Magazine, https://doi.org/10.1017/S0016756818000808, Fig. 3) it corresponds to the 99.9899% CL. In this case I recommend elaborating more on what the FDR is or removing this sentence.

Response: Thank you very much for pointing out our careless mistake. We have already deleted it.

L618: Extended data fig. 3: estimated sedrate of 0.7 cm/kyr, but tested sedimentation rate range was limited to 0 to 2 cm. Have the authors tried this analysis with a larger range of sedimentation rates?

Response: We have tried the analysis with a larger range of the sedimentation rates (see figure 14 below). Most of the calculated changes in sedimentation rates based on astronomical calibration tracks with those obtained from the eTimeOpt. One exception occurs in the 160-190 m interval. An average sedimentation rate of ~2 cm/kyr was calculated via eTimeOpt, and this value is four times that obtained by astronomical calibration. Tuning the GR data based on this sedimentation rate would have reduced 10 eccentricity cycles observed in this interval to 2~3 cycles, and ~2 Myr of time would 'disappear' incompatible with the biostratigraphic framework. We believe that it is necessary to work within the constraints of the initial temporal framework when studying deep-time cyclostratigraphy. Breaking away from other geochronological constraints and only following mathematical methods may lead to very incorrect conclusions. In fact, no lithological or facies changes can be observed in this horizon during the field work, which makes it difficult to support a sudden increase in sedimentation rates. We hence keep the original results of eTimeopt.

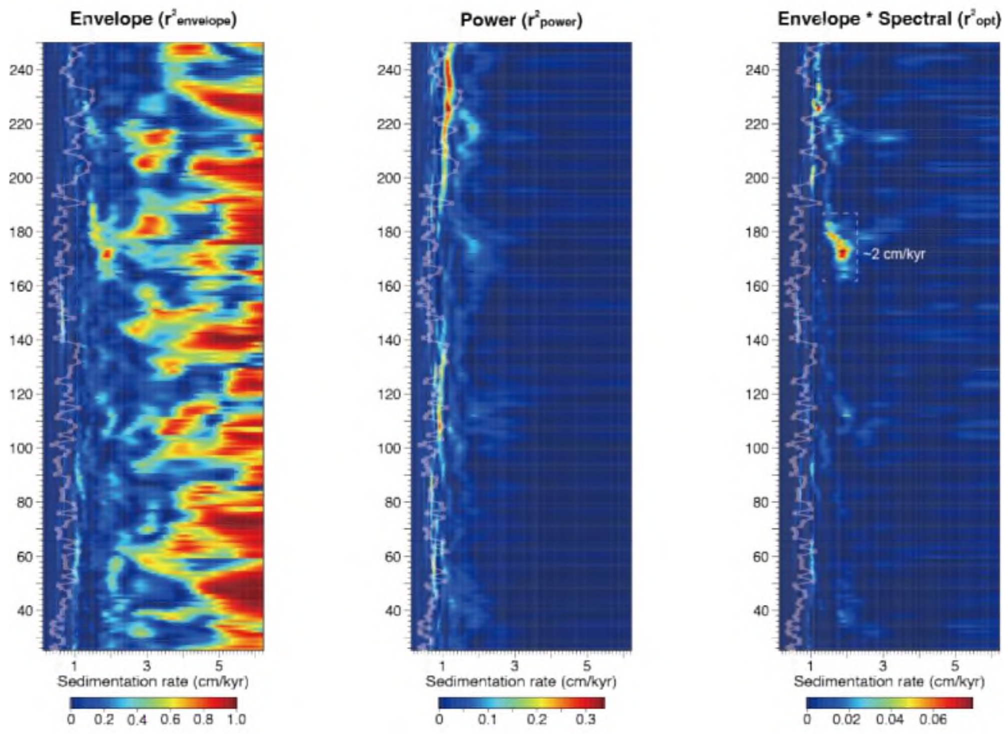

figure 14 eTimeOpt for the GR data from the Narao section

Test sedimentation rates (n = 200) are from ~0-6 cm/kyr, with sliding windows of 25 m and a step of 1 m. The gray line in all panels is the calculated sedimentation rate based on the 405-kyr astronomical calibration.

Minor comments:

3 minor points

L39: Why do you refrain from using the kyr and Myr abbreviations commonly used for durations? i.e. 104–105-yr !10 kyr to 100 kyr etc.

Response: Our mistake; this has been changed.

L70: The carbonates from … were deposited in a deep-marine environment.

Response: Resolved.

L78: "stable component throughout the geological history" needs to be changed in light of major comments.

Response: Thank you very much for pointing it out. We have changed into '**Although the stability of the 405-kyr eccentricity cycle in the deep time remains a topic of ongoing debate[28,29], geochronological evidence supports its relatively stable period during much of the Carboniferous[30-32], which is further substantiated by results derived from the ZB23-N64 astronomical solution[33] (Supplementary Fig. 3).**'

L95: "These periods are indistinguishable to those obtained for the astronomical solution (~1.2 Myr and ~2.4 Myr) in the Cenozoic Era19,24 (Extended Data Fig. 5)." There are many different astronomical solutions. Even in the two citations given, there are already 5 (La04, La10a-d).

Response: We have changed it into "**Even though the orbits of planets in the Solar System were proposed to be chaotic at timescales of 10–100 Myr[33], the periods of million-year-scale astronomical cycles are indistinguishable from those obtained by the astronomical solution (e.g., ZB18a astronomical solution) from the past 36 Ma[36] (Supplementary Fig. 6). It likely supports the non-chaotic behavior of the solar system during the main phase of the penultimate icehouse.**"

L597: Photos in Extended data fig 1. seem washed out (backlit, from the glare in the top right of the middle bottom photo?) and could do with some contrast enhancement. It is hard to see the patterns indicated with the lines.

Response: The Narao section is situated along a narrow country road that features a steep cliff, which poses challenges for capturing panoramic views in photographs. We have tried our best to modify and replace the photos to make them look clearer. Please see figure 15 below (it is included in the revised Supplementary Fig. 1.

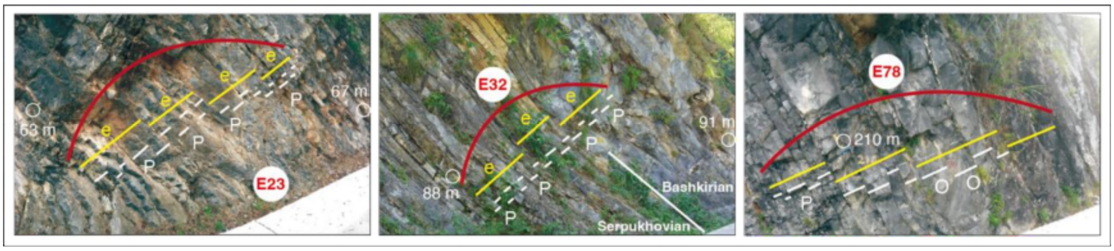

figure 15 Milanković cycles recorded in the lithological combination in Narao section.

Response to reviewer #3:

General comments:

The manuscript uses a new dataset of densely sampled marine carbon isotopes from the Carboniferous of China to argue for the cyclicity of orbital forcing to be driving the patterns and then draws comparison between Paleozoic and Cenozoic ice ages to argue that have a similar time course due to their shared influence of orbital forcing. **It is a provocative perspective and was interesting to read**, but it felt increasingly tenuous as the manuscript progressed and the later sections took off at a gallop with more and more connections and new ideas thrown in, each stated as a positive truth without distinguishing between what was directly observable in the rock record versus a modeling-based suggestion or what was a global pattern versus a local pattern, and with no reflection on what it would mean if any of the assertions expressed with certainty happened not to be true. And once the end was reached it was surprising to see that there never was acknowledgment of all the other factors that might have been driving climate that were left untouched or that it was a choice to truncate the length of the Paleozoic ice age to match up with the time course of the Cenozoic ice age and there was no justification of why that choice was appropriate. Finally, much of the material is far from my expertise and there were many interesting assertions that were new to me backed up by literature that I didn't know, so I had homework to do. And in following up with those references, they were not always appropriate for how they were being used. Honestly, the manuscript would be stronger if the second half was cut off and the authors focused in on the isotopic record, the potential periodicity in that record, and comparison to Cenozoic glacial forcing.

Response: Thank you very much for all the suggestion and comments. We address all of your concerns in the following responses to each issue.

Major comments:

First, it is true that there has been a big change in perspective in recent years regarding the causes of glaciation. In previous decades, continental configurations and how they channeled ocean currents with their heat transport and salinity differences were given great emphasis with the opening of the Southern Ocean and the closing of the Isthmus of Panama. Recent literature has focused much more heavily on CO_2 as the key driver above all else. So, the emphasis here has precedent, and yet the comparison is between the Cenozoic (when continents have been maximally dispersed) and the Carboniferous (with Euramerica and the coalbeds discussed in this paper being the key link in the assembly of Pangea). Shouldn't there be some discussion of Pangea versus Cenozoic geography, even if just to argue that it doesn't matter and justify why? At the least the geometry of Pangea should matter for the discussion of the changing latitudes of coal deposits—in part, that reflects where there was land in a pangean world.

As another example of what is not considered, the authors draw their record of glaciation through the Carboniferous from reference 55 (Soreghan et al. 2019), but that paper is an argument that volcanism is a crucial driver of Paleozoic volcanism—as has also been argued for the Ordovician glaciation in recent years. And yet, there is no

mention of volcanism at all in the current manuscript. It fits into a general concern with the manuscript in that arguments build out further and further in a straight line—culminating in orbital forcing causing both human and tetrapod evolution—but there never seems to be consideration of what it would mean if any of the logical connections in the series might be wrong or incomplete.

Response: Thank you for the suggestion. We fully appreciate that for much of the LPIA, changes in paleo-CO_2, temperatures, and glaciation history, were influenced to varying degrees by plate convergence, continental collisions, and changes in ocean circulation patterns, in particular during its onset and early phases of stepwise cooling and continental ice accumulation. We have taken several steps to address the reviewer's well-founded comments.

First, we added discussion of the different paleogeographies of the two icehouses: '**Since the late Paleozoic Era, continental areas in the Northern Hemisphere have consistently increased in extent relative to those in the Southern Hemisphere[9]. Specifically, the Pangaean supercontinent had large areas of southern high-latitude land and the Central Pangaean Mountains extended across tropical continent (Fig. 1a), which is substantially different with the late Cenozoic paleogeography[9] (Fig. 1b). The discovery in this study of analogous thresholds, despite the different paleogeographic background, enhances confidence in a more comprehensive comparison of the evolutionary patterns between the two icehouses, e.g., climate rhythmicity and transient events.**'

Second, we added discussion of tectonic time-scale climate changes, "**With a comprehensive understanding of Cenozoic climate, there is a tendency to attribute the Cenozoic cooling/warming events to multiple contributing factors. Revisiting the Eocene–Oligocene climatic transition as an example, its primary drivers include the tectonic-oceanic hypothesis and the CO_2 hypothesis. The first hypothesis posits that the enhanced thermal isolation of Antarctica, due to the widening of oceanic passages (such as the Drake and Tasman seaways), facilitated the expansion of Antarctic glaciation[54]. The second suggests that the dominating factor in the latest Eocene ice expansion is the decline in atmospheric CO_2 levels down to a specific threshold[55]. However, the predominant forcing of the climate changes remains a subject of debate, due to the intricate feedback mechanisms among the ocean, atmosphere, and Earth's surface. Classically, climate regulation on Earth is predominantly associated with atmospheric pCO_2 levels, which are influenced by the variations in carbon sources (e.g., volcanism) and sinks (e.g., continental weathering, organic carbon burial on land or in the ocean)[56]. There is no clear consensus regarding the factors responsible for long-term climatic shifts; debates often focus on whether carbon sources or sinks have been more influential. For instance, studies have connected both carbon sources or sinks to major climate changes of the LCIA[57-59], and this is also the case for the LPIA[47,53,60]. Both factors were largely controlled by plate tectonics, resulting in gradual changes that predominantly occurred unidirectionally over multi-million-year timescales.**"

Third, we added some comments to explain the observed quasi-synchronization of the transient climatic events in two separated icehouses, which is "**Beyond atmospheric CO_2 levels, the extraterrestrial factors may play a significant role in maintaining quasi-synchronization of these transient climatic events between two icehouses. We propose that tectonically controlled CO_2 variations brought the climate to threshold conditions, and then orbital forcing may have modulated these transient climatic events (thereby determining their timing), as a means of providing a rapid push across critical thresholds. The shifts in other boundary conditions—such as paleogeography and topography, oceanic gateway locations and bathymetry—cannot fully account for the climatic quasi-synchronization due to their independent changes within two icehouses.**"

Also, to address the reviewer's concern about the reference to the Soreghan et al. (2019) paper and inquiry as to why volcanism is not addressed in the manuscript, we provide the following response. Major changes in the amount of sulfate aerosols in the atmosphere triggered by volcanic emissions are not relevant to this study as their impact on climate is on the yearly to decadal scale. Continued volcanism could extend this to hundreds to 10s of 1000s of years if recurrent volcanic eruptions occurred proximal to one another. That said, once the short-term impact of individual (or closely spaced multiple) volcanic events waned (in years to decades at best), it is the longer-term process of silicate weathering due to release of C-based greenhouse gasses during eruption that would have the larger-scale impact — i.e., CO_2 drawdown through silicate weathering — and which would most closely couple the C cycling and climate (see papers by K. Maher, Godderis, and many more). Therefore, we focus on C-cycle and astronomical forcing components of climate change during the LPIA. We consider that this is the key factor to keep the quasi-synchronization of the climate-carbon cycle in two separated icehouses

Additionally, we have removed the section related to biological evolution, as the reviewer indicated it may extend beyond the scope of our research.

Another bigger thing to consider is that the manuscript is built around the comparison of Cenozoic and Carboniferous time series and how both go through a transition about 7 Myr before the end, and there is even the fortuitous simplicity of the durations and dates matching up with 35-0 Ma for the Cenozoic and 335-300 Ma for the Carboniferous. The explicit sentence in the Abstract is "Paralleling LPIA (335–301 Ma) and LCIA (34 Ma to the present) records using this event, we reveal the quasi-synchronization in the interaction of astronomical forcing, carbon cycling and glacial events from the onset to apex of two icehouses." However, only one of those time series is complete. That Soreghan reference source that is used has glaciation continually from the Late Devonian through most of the Permian. How could there be quasi-synchronization from onset to apex if the Paleozoic onset is about 30 Myr earlier than the truncated onset of the include time series and the Paleozoic peak is in the Permian

just after the manuscript's time series ends. Overall, the included Paleozoic time series only about 40% of the total time series and includes neither onset nor apex.

Response: We disagree that the LPIA extended from the late Devonian through early to middle Permian. There is a plethora of studies that have documented that this is not the case. Yes, you are very correct that continental ice accumulation occurred intermittently from the late Devonian through early Permian but for the following reasons, the LPIA is considered to have initiated — with stepwise cooling and ice accumulation — between 335 and 330 Ma and to have reached its peak proximal to the Carboniferous–Permian boundary (~300 Ma).

Given the availability of high-precision U-Pb zircon ages for glacial deposits in Gondwana (Southern Hemisphere), it is now possible to much more critically evaluate the timing and geographic extent of events of major continental ice build-up through the latter half of the Paleozoic. Figure 1 of Montañez (2022) presents the temporal and geographic distribution of paleo-glaciers in the Southern Hemisphere during the LPIA. Given these geochronologic constraints, it is now evident that the onset of the LPIA is in the mid-to-late Visean. Evidence of the onset of glaciation and stepwise increase in continental ice beginning ~335–330 Ma beyond the initial presence of glacial deposits and indicators in many basins across Gondwana, please see a review in Montañez (2022). It also includes the onset of low-latitude deposition of meter-scale stratigraphic cycles with erosive/karstified tops suggesting 10s of meters of eustatic change on the 10^4-yr scale, and multiple lines of geochemical evidence (again see Montañez 2022 for a full review of all of these aspects).

During the late Devonian–Mississippian, there is evidence of continental ice accumulation. Extensive glaciation occurred during the Famennian Stage of the latest Devonian Period. The distribution of glacial deposits in the latter half of the Paleozoic further suggests possible glaciation in late middle to early late Tournasian (earliest Mississippian); however, geographically quite restricted with minimal chronostratigraphic constraints on the age of these deposits (please see the details in Montañez, 2022). It also has been proposed that the Late Devonian glaciation is distinguishable from the LPIA given that it bears greater resemblance to the Late Ordovician glaciation due to its short duration and possible loss of large-scale ice accumulation (albeit there may have been alpine glaciers) (e.g., Raymond and Metz, 2004) over the 20 Myr period between the end of the Devonian glaciation and the onset of evidence for large scale accumulation of continental ice in the Visean.

Given that the chronostratigraphically constrained glacial deposits across the Southern Hemisphere indicate that the late Devonian glaciation was discrete from that of the LPIA by 10s of millions of years and that the evidence for geographically restricted glaciers in the early Mississippian (Tournasian and early Visean; 360 to 340 Ma) is very poorly dated (see Fig. 1 of Montañez, 2022), we consider (as have many others) that the LPIA spanned ~335–330 Ma to 280 Ma with peak glaciation between 300 and 290 Ma (an interval spanning the latest stage of the Carboniferous and the first 1 to 2 stages

of the Permian). This temporal scenario for the LPIA is presented in dozens of published papers with some debate as to whether the apex of glaciation was in the latest Carboniferous stage (Gzhelian) or earliest Permian. Whether the peak of the glaciation was in the earliest Permian (1st 2 stages: Asselian and Sakmarian as proposed by Soreghan et al. 2019) or in the latest Carboniferous (Fielding et al. 2015; Macdonald et al. 2019; Griffis et al., 2018, 2021, 2023) remains debated. Newly published boron isotope composition of aqueous borate indicate the CO_2 was the lowest round 300 ± 5 Ma (Jurikova et al., 2025). Consequently, the comparison of the Cenozoic and Paleozoic time series in this manuscript is of two records that both span the onset to peak of the ice ages. We addressed this overall concern in the beginning of the manuscript, as "**This new $\delta^{13}C_{carb}$, integrated with a GR time series, span the interval from the onset (~335–330 Ma[8]) to apex (300 ± 5 Ma[22]; referred to as 300 ± 1 Ma[23] herein) of the LPIA, providing new insight into the response of global carbon cycling to astronomical forcing during the penultimate icehouse.**".

We addressed this overall concern in the section 'Astrochronologic constraints on the Carboniferous climate-carbon cycle history', as '**Three positive $\delta^{13}C_{carb}$ excursions during the late Visean–earliest Serpukhovian interval (red bars in Fig. 3e) overlap with the proposed timings of the stepwise onset of the LPIA[8] (Fig. 3b). Astrochronologic constraints for England shallow marine carbonates from the U.K. indicate a basal late Asbian age of 334.48 ± 0.35 Ma[40]. Thus, the $\delta^{13}C_{carb}$ positive excursion at ~335–334.6 Ma is likely time-equivalent with a cooling pulse inferred from an earliest Asbian positive shift in biogenic apatite $\delta^{18}O$ values and ice buildup[41], suggesting a possible mechanistic link between the onset of the LPIA and the enhanced carbon burial.**'

And finally, there is a series of problematic referencing that adds to the tenuousness:
Response: Thank you for pointing out the referencing problem. We apologize for any miscommunication or errors on our part. This issue partly stems from errors in the numbering of references during the editing process; however, this is also misunderstandings regarding the cited references. Below, we provide a full response addressing each point individually.

"Net positive terrestrial carbon sequestration occurred during late Carboniferous glacials driven by the predominance of wetland forests (13)." (Lines 58-60)
Response: We have changed the cited reference to Montañez et al. (2016; Nature Geoscience), in which they indicate, "In stark contrast to the Late Pleistocene when the terrestrial organic carbon reservoir served as a C sink during interglacials[29], a net positive terrestrial C sink was established during late Palaeozoic glacials due to the unprecedented geographic expanse and carbon sequestration potential of the palaeo-tropical wetland forests"

“A loss of arborescent lycopsids would decrease the terrestrial carbon burial rates at low latitudes during glacial periods (13), which would have shifted the centre of terrestrial carbon burial from low to high latitudes.” (Lines 156-158)
Response: We have provided more explanation on this issue in the third paragraph of the section ‘Eccentricity-scale climate-carbon cycling’, as “**In contrast, we propose that for the subsequent interval between ~307–300 Ma, eccentricity minima led to cold and dry climate conditions, resulting in a shrinkage of the ^{12}C-enriched continental carbon reservoirs at high latitudes. This shift in carbon cycle dynamics coincides with a global cooling (~307–304.5 Ma), characterized by ~50% drop in pCO_2[10,47] (Fig. 3d), a short-lived but intense glaciation[32] (Fig. 3f, i) (Fig. 3c). The subsequent increase of two to three times in pCO_2 during the Kasimovian–Gzhelian transition (Fig. 3d) appears to be insufficient to reverse this dynamic (Fig. 3j). We speculate that the centre of terrestrial carbon burial may have shifted from the low to high latitudes in response to the onset of long-term tropical aridification at the close of the Middle Pennsylvanian. Key evidence supporting this hypothesis includes precipitous loss of ‘coal’ forests and peat burial by end of the Middle Pennsylvanian[14,48], which served as the primary source of terrestrial carbon sequestration prior to 307 Ma[20]. Furthermore, ecosystem modeling of late Carboniferous terrestrial ecosystems indicates major changes in high-latitude forest cover during glacial and interglacial extremes in particular the higher tolerance of drought-adapted vascular plants to freezing conditions[16], highlighting the potential for the increasing importance of different high-latitude carbon reservoirs, e.g., tundra, ices, and boreal forests, in response to climate deterioration and amelioration. We suggest that global carbon cycle dynamics could have been substantially influenced by such spatial redistribution in high-latitude biomes. Glacial advances during attenuated eccentricity cycles would have caused an upsurge in tundra areal extent at high latitudes, accounting for the anti-phase relationship observed between $\delta^{13}C$ and GR after ~307 Ma.**”

None of that is really in reference 13. The only mention of glaciation is a reference to the same DiMichele 2014 paper that is already reference 10 in this manuscript (although incorrectly cited as DiMichele & Herendeen 2014). And no claim is made that lycopsids are uniquely important; instead it is emphasized that carbon sequestration remains high once the transition occurs to tree fern dominance.
“At ~307 Ma, most lycopsids abruptly disappeared (14), which potentially invokes a significant change in the global carbon cycle to orbital forcing.” (Lines 60-61)
This is cast as a global statement, but that reference is specific to Peoria, Illinois as a local venue to speak about Euramerica. What about all of the lycopsids that persisted in eastern Pangea and China?
Response: Thank you so much for this point. During the decline in pCO_2, the aerial extent of the ‘coal’ forests dropped by half (Cleal and Thomas, 2005). And within the short-term CO_2 low arborescent lycopsids of the wetland forests did go extinct throughout Euramerica and seasonally dry tropical floras shifted from cordaitalean dominance to walcian conifer dominance (DiMichele et al. 2009; Falcon-Lang et al.

2018). That these occurred coincidently with a drop in CO_2 below 400 ppm supports the previously hypothesized CO_2 threshold theory for the Pennsylvanian ecologic turnovers (Beerling et al. 1998; Beerling and Berner 2000; Montanez et al. 2016). The only lycopsids that continued to exist after this time are those in Cathaysia. This is all discussed in Richey et al. 2020 (p. 1767 to 1768).

In the revived manuscript, we stress the potential importance of consistent loss of tropical ‘coal’ forests during the long-term pantropical aridification, instead of focusing the importance of a specific vegetation of lycopsids. We have edited the reference and changed this part into "**At 307 Ma, arborescent lycopsids abruptly disappeared from Euramerican biomes[17,18], coincident with the onset of long-term tropical aridification[18], remaining only in Cathaysia (China)[20,21] that hold everwet condition through the late Paleozoic[19]. The continental-scale aridification likely intensified the repeated vegetational turnovers that characterized the remainder of the LPIA[12]. Thus, it is possible that the widespread loss of tropical ‘coal’ forests may have led to a change in the response of the global carbon cycle to astronomical forcing. This hypothesis, however, remains untested.**"

"Both Earth System modeling (33) and empirical records (35) reveal that shifts in tundra and boreal forest ecosystems occurred in response to orbitally driven climatic change during the late Carboniferous. Global carbon cycle dynamics may have been affected by such changes in land cover given major differences in the carbon storage capacities between the high-latitude biomes (33)." (Lines 160-164). That empirical record 35 is from a single place and time, how could it record a shift? And no mention is made of orbital forcing in 35. Reference 33 does address the topic of that first sentence regarding tundra versus forest and orbital forcing, but it does not touch on the carbon storage of that second citation. However, even the first 33 reference feels like a misrepresentation as far as the way that reference is used. The modeling employs modern biome characteristics and applies them to the Paleozoic even though the conifers, flowering plants, and even the lichens of those modern biomes had not evolved in the Paleozoic. What they did in that paper was correct in the context of that paper, which was show that they couldn't get the glacial cycling without including a dynamic vegetation model, but that does not amount to endorsing a particular vegetational distribution from model outputs or that the characteristics of the vegetation in the model reflects reality.

Response: Thank you very much for the constructive suggestion. We misunderstood the cited references. We re-wrote this part as ‘**In contrast, we propose that for the subsequent interval between ~307–300 Ma, eccentricity minima led to cold and dry climate conditions, resulting in a shrinkage of the ^{12}C-enriched continental carbon reservoirs at high latitudes. This shift in carbon cycle dynamics coincides with an ~50% drop in pCO_2[10,47] (Fig. 3d), a short-lived but intense glaciation[32] (Fig. 3f, i), and loss of arborescent lycopsid throughout the Euramerican tropical rainforests[11,13] (Fig. 3c). We propose that the centre of terrestrial carbon burial may have shifted from the low to high latitudes in response to the onset of tropical**

aridification at the close of the Middle Pennsylvanian coupled with short-term intensified cooling. Key evidence supporting this hypothesis includes precipitous loss of 'coal' forests and peat burial by end of the Middle Pennsylvanian[14,48], which served as the primary source of terrestrial carbon sequestration prior to 307 Ma[20]. Furthermore, ecosystem modeling of late Carboniferous terrestrial ecosystems indicates major changes in high-latitude forest cover during glacial and interglacial extremes in particular the higher tolerance of drought-adapted vascular plants to freezing conditions[16], highlighting the potential for the increasing importance of different high-latitude carbon reservoirs, e.g., tundra, ices, and boreal forests, in response to climate deterioration and amelioration. We suggest that global carbon cycle dynamics could have been substantially influenced by such spatial redistribution in high-latitude biomes. Glacial advances during attenuated eccentricity cycles would have caused an upsurge in tundra areal extent at high latitudes, accounting for the anti-phase relationship observed between δ^{13}C and GR after ~307 Ma.'

"First, Carboniferous climate simulations reveal that ocean circulation strengthened during glacials (36). Stronger ocean circulation during glacial phases would have favored enhanced transfer of organic matter to deep waters via the biological pump, causing a decrease in deep-sea $\delta^{13}C_{DIC}$. (Lines 170-173)

Why would stronger circulation enhance the biological pump? Is this about nutrient upwelling? No explanation is offered and that the reference included with the lead-in sentence does not mention the biologic pump.

"Third, reduced atmospheric pCO_2 would have had a profound impact on the paleo-plants' physiological forcing on the hydrological cycle that would have altered weathering patterns (38) and regional marine carbon burial." (Lines 178-181).

Weathering comes up only once, and it is an aside mention that if evapotranspiration decreased, then runoff would increase and maybe that would affect weathering. But, as they note, that would only be true if precipitation does not change with evapotranspiration, when surely it does. So, again, there is no relevance here.

Response: Thank you very much for the constructive suggestion. Based on your suggestion, we have re-written this part into "**Although we tend to support the biome areal competition hypothesis, other studies have stressed the relevance of organic carbon burial in the ocean during the LPIA[49-51]. Iron-rich dust flux likely increased substantially during late Carboniferous glacials, which may have stimulated marine productivity that would have enhanced marine carbon burial[49,51]. This hypothesis was proposed to explain the secular decoupling of continental weathering and *p*CO_2 after ~303 Ma[50]. Despite more vigorous ocean circulation at glacial phases during late Carboniferous[52], it remains uncertain as to which boundary conditions would have driven reversals in the response of organic carbon burial in the ocean to astronomical forcing. Given the great capacity of the terrestrial biosphere to influence the LPIA carbon cycling[10], we view the areal competition between low- and high-latitude biomes as a more plausible explanation for the observed shift in phase relationships of the**

eccentricity-scale climate-carbon cycling at ~307 Ma. Future investigations will be necessary to determine whether climate-carbon cycle feedbacks shifted along major deglaciation events and vegetation turnovers during the early Permian period[53].”

References referred to in responses and not assigned #s (refs in main text).

Barnett, A.J., Wright, V.P., 2008. A sedimentological and cyclostratigraphic evaluation of the completeness of the Mississippian–Pennsylvanian (Mid-Carboniferous) global stratotype section and point, Arrow Canyon, Nevada, USA. Journal of the Geological Society 165, 859-873.

Beerling, D.J., Woodward, F.I., Lomas, M.R., Wills, M.A., Quick, W.P., Valdes, P.J., 1998. The influence of Carboniferous palaeoatmospheres on plant function: an experimental and modelling assessment. Philosophical Transactions of the Royal Society B: Biological Sciences 353, 131-140.

Beerling, D.J., Berner, R.A., 2000. Impact of a Permo-Carboniferous high O_2 event on the terrestrial carbon cycle. Proceedings of the National Academy of Sciences 97, 12428-12432.

Bishop, J.W., Montañez, I.P., Gulbranson, E.L., Brenckle, P.L., 2009. The onset of mid-Carboniferous glacio-eustasy: Sedimentologic and diagenetic constraints, Arrow Canyon, Nevada. Palaeogeography, Palaeoclimatology, Palaeoecology 276, 217-243.

Blake, B.M., Beuthin, J.D., 2008. Deciphering the mid-Carboniferous eustatic event in the central Appalachian foreland basin, southern West Virginia, USA. Geological Society of America Special Papers 441, 249-260.

Buggisch, W., Wang, X., Alekseev, A.S., Joachimski, M.M., 2011. Carboniferous–Permian carbon isotope stratigraphy of successions from China (Yangtze platform), USA (Kansas) and Russia (Moscow Basin and Urals). Palaeogeography, Palaeoclimatology, Palaeoecology 301, 18-38.

Chen, J., Montañez, I.P., Qi, Y., Wang, X., Wang, Q., Lin, W., 2016. Coupled sedimentary and $\delta^{13}C$ records of late Mississippian platform-to-slope successions from South China: Insight into $\delta^{13}C$ chemostratigraphy. Palaeogeography, Palaeoclimatology, Palaeoecology 448, 162-178.

Chen, J., Montañez, I.P., Qi, Y., Shen, S., Wang, X., 2018. Strontium and carbon isotopic evidence for decoupling of pCO_2 from continental weathering at the apex of the late Paleozoic glaciation. Geology 46, 395-398.

Chen, J., Montañez Isabel, P., Zhang, S., Isson Terry, T., Macarewich Sophia, I., Planavsky Noah, J., Zhang, F., Rauzi, S., Daviau, K., Yao, L., Qi, Y.-p., Wang, Y., Fan, J.-x., Poulsen Christopher, J., Anbar Ariel, D., Shen, S.-z., Wang, X.-d., 2022. Marine anoxia linked to abrupt global warming during Earth's penultimate icehouse. Proceedings of the National Academy of Sciences 119, e2115231119.

Davydov, V.I., Crowley, J.L., Schmitz, M.D., Poletaev, V.I., 2010. High-precision U-Pb zircon age calibration of the global Carboniferous time scale and Milankovitch band cyclicity in the Donets Basin, eastern Ukraine. Geochem. Geochemistry Geophysics Geosystems 11, Q0AA04, doi:10.1029/2009GC002736.

DiMichele, W.A., Montañez, I.P., Poulsen, C.J., Tabor, N.J., 2009. Climate and vegetational regime shifts in the late Paleozoic ice age earth. Geobiology 7, 200-226.

Eros, J.M., Montañez, I.P., Osleger, D.A., Davydov, V.I., Nemyrovska, T.I., Poletaev, V.I., Zhykalyak, M.V., 2012. Sequence stratigraphy and onlap history of the

Donets Basin, Ukraine: insight into Carboniferous icehouse dynamics. Palaeogeography, Palaeoclimatology, Palaeoecology 313, 1-25.
Falcon-Lang, H.J., Nelson, W.J., Heckel, P.H., Dimichele, W.A., Elrick, S.D., 2018. New insights on the stepwise collapse of the Carboniferous Coal Forests: Evidence from cyclothems and coniferopsid tree-stumps near the Desmoinesian–Missourian boundary in Peoria County, Illinois, USA. Palaeogeography Palaeoclimatology Palaeoecology 490, 375-392.
Fielding, C.R. and Frank, T.D. 2015. Onset of the glacioeustatic signal recording late Palaeozoic Gondwanan ice growth: new data from palaeotropical East Fife, Scotland. Palaeogeography, Palaeoclimatology, Palaeoecology, 426, 121-138.
Fielding, C., 2021. Late Palaeozoic cyclothems–A review of their stratigraphy and sedimentology. Earth-Science Reviews 217, 103612.
Griffis, N.P., Mundil, R., Montañez, I.P., Isbell, J., Fedorchuk, N., Vesely, F., Iannuzzi, R., Yin, Q.Z., 2018. A new stratigraphic framework built on U-Pb single-zircon TIMS ages and implications for the timing of the penultimate icehouse (Paraná Basin, Brazil). Geological Society of America Bulletin 130, 848-858.
Griffis, N., Montañez, I., Mundil, R., Le Heron, D., Dietrich, P., Kettler, C., Linol, B., Mottin, T., Vesely, F., Iannuzzi, R., 2021. High-latitude ice and climate control on sediment supply across SW Gondwana during the late Carboniferous and early Permian. GSA Bulletin 133, 2113-2124.
Griffis, N., Mundil, R., Montanez, I., Le Heron, D., Dietrich, P., Iannuzzi, R., 2023. A Carboniferous Apex for the late Paleozoic Icehouse. Geological Society, London, Special Publications 535, SP535-2022-2256.
Goddéris, Y., Donnadieu, Y., Carretier, S., Aretz, M., Dera, G., Macouin, M., and Regard, V.: Onset and ending of the late Palaeozoic ice age triggered by tectonically paced rock weathering, Nature Geoscience, 10, 382-386.
Heckel, P.H., 1986. Sea-level curve for Pennsylvanian eustatic marine transgressive-regressive depositional cycles along midcontinent outcrop belt, North America. Geology 14, 330-334.
Heckel, P., 2008. Pennsylvanian cyclothems in Midcontinent North America as far-field effects of waxing and waning of Gondwana ice sheets. Special Paper of the Geological Society of America 441, 275-289.
Heard, T.G., Pickering, K.T., Robinson, S.A., 2008. Milankovitch forcing of bioturbation intensity in deep-marine thin-bedded siliciclastic turbidites. Earth and Planetary Science Letters 272, 130-138.
Hinnov, L.A., Wu, H., Fang, Q., 2016. Reply to the comment on ‘Geologic evidence for chaotic behaviour of the planets and its constraints on the third-order eustatic sequences at the end of the Late Paleozoic Ice Age’ by Qiang Fang, Huaichun Wu, Linda A. Hinnov, Xiuchun Jing, Xunlian Wang and Qingchun Jiang [Palaeogeography, Palaeoclimatology, Palaeoecology, 400 (2015), 848-859]. Palaeogeography, Palaeoclimatology, Palaeoecology 461, 475-480.
Kemp, D.B., 2016. Optimizing significance testing of astronomical forcing in cyclostratigraphy. Paleoceanography 31, 1516-1531.

Laepple, T., Lohmann, G., 2009. Seasonal cycle as template for climate variability on astronomical timescales. Paleoceanography 24. PA4201, doi:10.1029/2008PA001674.

Lane, H.R., Brenckle, P.L., Baesemann, J., Richards, B., 1999. The IUGS boundary in the middle of the Carboniferous: Arrow Canyon, Nevada, USA. Episodes 22, 272-283.

Macdonald, F.A., Swanson-Hysell, N.L., Park, Y., Lisiecki, L., Jagoutz, O., 2019. Arc-continent collisions in the tropics set Earth's climate state. Science, 364, 181–184

Maher, K. and Chamberlain, C. P.: Hydrologic Regulation of Chemical Weathering and the Geologic Carbon Cycle, Science, 343, 1502-1504.

Martin, L.G., Montañez, I.P., Bishop, J.W., 2012. A paleotropical carbonate-dominated archive of Carboniferous icehouse dynamics, Bird Spring Fm., Southern Great Basin, USA. Palaeogeography, Palaeoclimatology, Palaeoecology 329, 64-82.

Meyers, S.R., 2019. Cyclostratigraphy and the problem of astrochronologic testing. Earth-Science Reviews 190, 190-223.

Montañez, I.P., McElwain, J.C., Poulsen, C.J., White, J.D., DiMichele, W.A., Wilson, J.P., Griggs, G., Hren, M.T., 2016. Climate, pCO_2 and terrestrial carbon cycle linkages during late Palaeozoic glacial-interglacial cycles. Nature Geoscience 9, 824-828.

Montañez, I.P., 2022. Current synthesis of the penultimate icehouse and its imprint on the Upper Devonian through Permian stratigraphic record. Geological Society, London, Special Publications 512, 213-245.

Payros, A., Martínez-Braceras, N., 2014. Orbital forcing in turbidite accumulation during the Eocene greenhouse interval. Sedimentology 61, 1411-1432.

Qi, Y., Barrick, J.E., Hogancamp, N.J., Chen, J., Hu, K., Wang, Q., Wang, X., 2020. Conodont faunas across the Kasimovian–Gzhelian boundary (Late Pennsylvanian) in South China and implications for the selection of the stratotype for the base of the global Gzhelian Stage. Papers in Palaeontology 30, 1-46.

Raymond, A., Metz, C., 2004. Ice and Its Consequences: Glaciation in the Late Ordovician, Late Devonian, Pennsylvanian-Permian, and Cenozoic Compared. The Journal of geology 112, 655-670.

Richey, J.D., Montañez, I.P., Goddéris, Y., Looy, C.V., Griffis, N.P., DiMichele, W.A., 2020. Influence of temporally varying weatherability on CO_2-climate coupling and ecosystem change in the late Paleozoic. Climate of the Past 16, 1759-1775.

Sadler, P.M., 1981. Sediment accumulation rates and the completeness of stratigraphic sections. The Journal of Geology 89, 569-584.

Saltzman, M.R., 2003. Late Paleozoic ice age: Oceanic gateway or pCO_2? Geology 31, 151–154.

Saunders, W.B., Ramsbottom, W.H.C., 1986. The mid-Carboniferous eustatic event. Geology 14, 208-212.

Schmitz, M.D., Davydov, V.I., 2012. Quantitative radiometric and biostratigraphic calibration of the Pennsylvanian–Early Permian (Cisuralian) time scale and pan-Euramerican chronostratigraphic correlation. Geological Society of America Bulletin 124, 549-577.

Tian, X., Chen, J., Yao, L., Hu, K., Qi, Y., Wang, X., 2019. Glacio-eustasy and $\delta^{13}C$ across the Mississippian–Pennsylvanian boundary in the eastern Paleo-Tethys Ocean (South China): Implications for mid-Carboniferous major glaciation. Geological Journal 55, 2704-2716.

Weedon, G.P., Page, K.N., Jenkyns, H.C., 2019. Cyclostratigraphy, stratigraphic gaps and the duration of the Hettangian Stage (Jurassic): insights from the Blue Lias Formation of southern Britain. Geological Magazine 156, 1469-1509.

Weedon, G.P., 2022. Problems with the current practice of spectral analysis in cyclostratigraphy: Avoiding false detection of regular cyclicity. Earth-Science Reviews 235, 104261.

Weltje, G., de Boer, P.L., 1993. Astronomically induced paleoclimatic oscillations reflected in Pliocene turbidite deposits on Corfu (Greece): implications for the interpretation of higher order cyclicity in ancient turbidite systems. Geology 21, 307-310.

Westerhold, T., Marwan, N., Drury, A.J., Liebrand, D., Agnini, C., Anagnostou, E., Barnet, J.S.K., Bohaty, S.M., Vleeschouwer, D.D., Florindo, F.J.e., 2020. An astronomically dated record of Earth's climate and its predictability over the last 66 million years. Science 369, 1383-1387.

Yang, W., Chen, J., Gao, B., Zhong, Y., Huang, X., Wang, Y., Qi, Y., Shen, K.-S., Mii, H.-S., Wang, X.-d., Shen, S.-z., 2023. Sedimentary facies and carbon isotopes of the Upper Carboniferous to Lower Permian in South China: Implications for icehouse to greenhouse transition. Global and Planetary Change 221, 104051.

Vaughan, S., Bailey, R.J., Smith, D.G., 2011. Detecting cycles in stratigraphic data: Spectral analysis in the presence of red noise. Paleoceanography 26, PA4211, doi:10.1029/2011PA002195.

Vaughan, S., Bailey, R.J., Smith, D.G., 2015. Cyclostratigraphy: data filtering as a source of spurious spectral peaks. Geological Society, London, Special Publications 404, 151-156.

Weedon, G.P., Page, K.N., Jenkyns, H.C., 2019. Cyclostratigraphy, stratigraphic gaps and the duration of the Hettangian Stage (Jurassic): insights from the Blue Lias Formation of southern Britain. Geological Magazine 156, 1469-1509.

Weedon, G.P., 2022. Problems with the current practice of spectral analysis in cyclostratigraphy: Avoiding false detection of regular cyclicity. Earth-Science Reviews 235, 104261.

Zeebe, R., Lourens, L., 2022. Geologically constrained astronomical solutions for the Cenozoic era. Earth and Planetary Science Letters 592, 117595.

Zeebe, R.E., Lantink, M.L., 2024. A Secular Solar System Resonance that Disrupts the Dominant Cycle in Earth’s Orbital Eccentricity (g_2-g_5): Implications for Astrochronology. The Astronomical Journal 167, 204.

Revision notes:

Responses to reviewers:

We appreciate the reviewers' comments, leading to significant improvements of our manuscript. Below the reviewers' comments, and we provide our responses to reviewer comments in blue text.

Response to reviewer #1:

General comments:

The various actions that the authors have taken to address my concerns are comprehensive, which I appreciate.

Response: We appreciate the positive comments on our efforts to improve our work. We have carefully evaluated the reviewer's suggestions and performed additional data analysis.

Major points:

I'm still intrigued by the observation that the inferred (abrupt) change from in-phase to anti-phase relation between $\delta^{13}C$ and gamma ray signal around ~189 m height (~307 Ma) coincides almost exactly with an abrupt change (i.e., increase) in inferred sedimentation rate (according to the cyclostratigraphic interpretation/405 kyr-tuned astrochronologic framework). Is that a pure coincidence, or could there be a link here? OR: could it be the case that an alternative cyclostratigraphic interpretation is possible for this upper interval -> e.g. can you exclude the possibility that in this upper/younger part of the record you're looking in fact at a different expression of dominant Milanković cycles in the GR series (and not necessarily a change in sedimentation rate)? For example, if the large-amplitude swings with a thickness of 4-5 m that start to appear above 189 m are not ~405 kyr eccentricity but instead obliquity amplitude modulation cycles around ~1 Myr, that would mean sedimentation rate remained relatively constant. And in terms of the phase relationship analysis, this would mean that one is comparing apples with oranges (i.e., eccentricity vs eccentricity between $\delta^{13}C$ and GR in the lower part of the record, eccentricity vs obliquity amplitude in the upper part of the record). I guess the alternative obliquity interpretation has not been tested with eTimeOpt, which does not consider obliquity + its AM components. I recommend to discuss this alternative possibility in the manuscript and/or explain why you think this is not a valid option, but then also explain the coincidence between an abrupt change in sedimentation rate and phase relationship.

Response: Thank you for your valuable feedback and suggestions. We have thoroughly checked our astronomical calibration results. Please see Figure 1 below, which includes the calculated sedimentation rate after 405-kyr calibration. A short-term increase in sedimentation rate is observed at ~190.75 m (indicated by 'arrow A'), corresponding to a thicker GR cycle labeled 'No. 72'. This increase can be calibrated to ~307.8 Ma, occurring ~0.8 Myr prior to the interval marked by a change in phase relation between GR and $\delta^{13}C_{carb}$ at ~195 m, as indicated by 'arrow C'. We think there is no direct connection between these two events due to the time difference.

Another increase in sedimentation rate is observed at ~196 m (indicated by 'arrow B') and remained stable throughout the Kasimovian interval at Narao. This increase in

sedimentation rate occurs immediately following a change in the phase relationship between GR and $\delta^{13}C_{carb}$. We attribute this shift in the phase relationship between climate and carbon cycles to the collapse of coal forests around 307 Ma. This event coincides with a decline in CO_2 levels (Montañez et al., 2016) as well as short-lived (~1 Myr) but intense glaciation development during the early Kasimovian (Eros et al., 2012; Richey et al., 2020; Montañez, 2022). The development of high-latitude glaciation resulted in lowered sea level at low latitudes, leading to shallower sedimentary environments in the studied regions. Consequently, this facilitated increased terrestrial inputs along with the elevated sedimentation rates.

We have added statements that address this in the main text: "An abrupt increase in the sedimentation rate (blue arrow in Fig. 1e), accompanied by enhanced terrestrial input (Fig. 1f), is observed at a stratigraphic depth of ~196 m, which has been astronomically calibrated to an age of ~306.9 Ma. We suggest that it may represent a regional response to glacio-eustatic sea-level lowering during the early Kasimovian[33], occurring within the context of secular cooling towards the Carboniferous–Permian transition[22,23]". We also have added a sentence in the Discussion section 'Eccentricity-scale climate-carbon cycling': 'The in-phase behaviour inverts to anti-phase at ~307 Ma (purple shading in Fig. 3j), along with an abrupt increase in sedimentation rate and GR values (Fig. 1e, f).'.

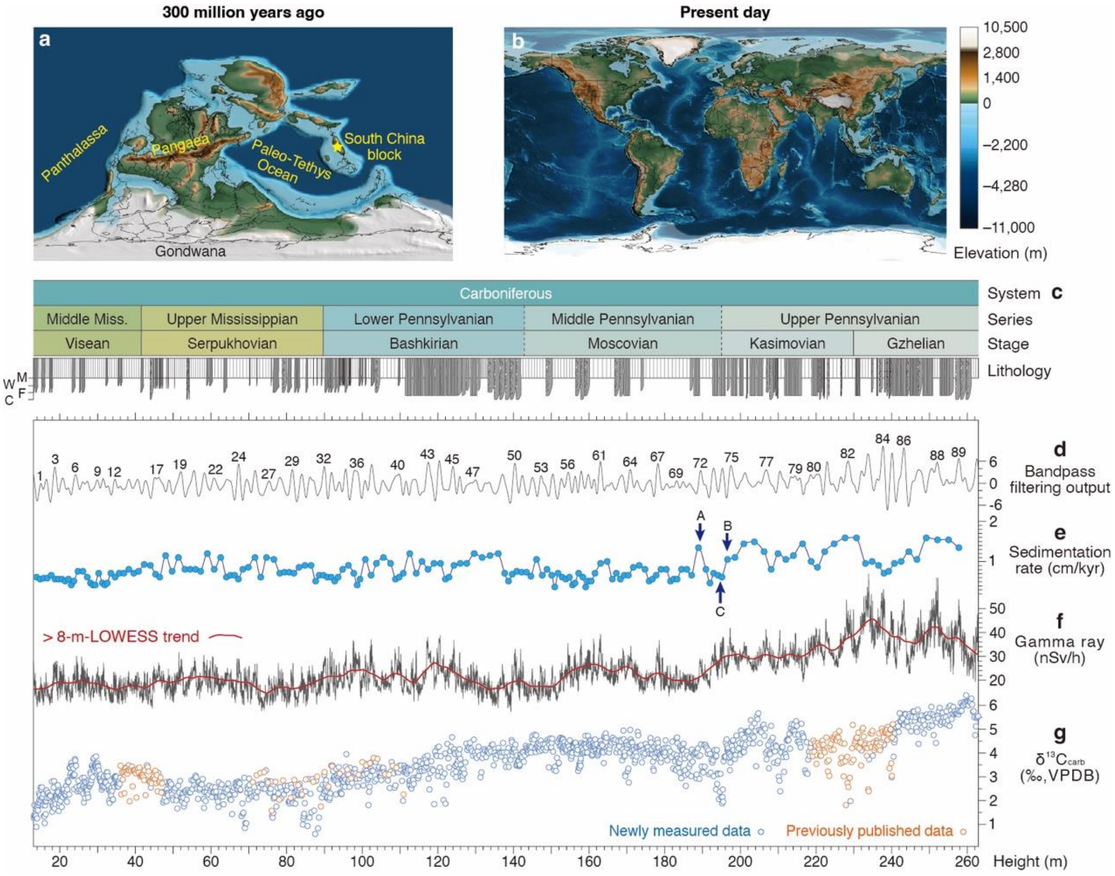

Figure 1 Newly revised Fig. 1 in the main text.

We understand your concerns regarding the validity of observed increases in sedimentation rate. We have tested for this with eTimeOpt analysis utilizing both the obliquity cycle and its long-period amplitude modulation cycle; however, we did not obtain a positive result. We remain confidence in our 405-kyr calibration, which is supported by robust stratigraphic correlations as follows. Global $\delta^{13}C_{carb}$ stratigraphic correlation indicates that the stratigraphic position of the basal Kasimovian in the Narao section can be estimated at ~195 m. Thus, an average sedimentation rate of ~1.05 cm/kyr is derived from dividing the 34.6 m of stratigraphic thickness between the basal Kasimovian and the basal Gzhelian (229.6 m; Chen et al., 2022) by a total duration of 3.3 Myr (Aretz et al., 2020). This result is consistent with astronomical calibration and eTimeOpt when calibrated to the 405-kyr cycle. Even though flaws exist in the eCOCO method, eCOCO helps to evaluate frequency ratios as predicted in astronomical solutions (including the obliquity frequency and amplitude). The eCOCO method also suggests an abrupt increase in the sedimentation rate at this level, as shown in Figure 2 below.

Given the consistency of sedimentation rates derived from astronomical calibration, eTimeOpt and eCOCO, coupled with a comprehensive stratigraphic analysis, we do not consider that it is necessary to separately address the validity of the astronomical calibration in this context at length; however, we have extended our discussion about it slightly in the revised manuscript.

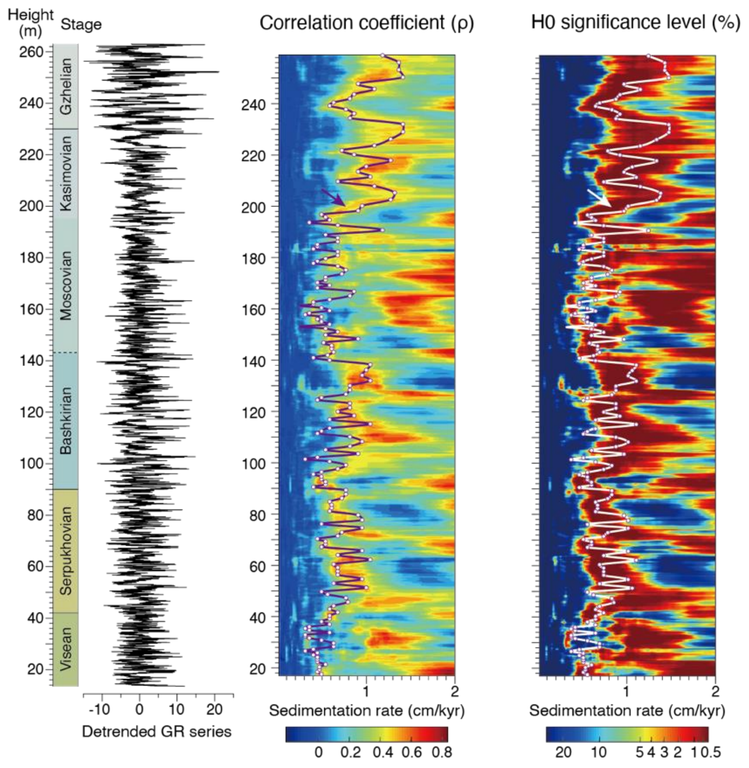

Figure 2 Sedimentation rate obtained from eCOCO.
Evolutionary null hypothesis (H0) and significance level of the GR. The lines are the calculated sedimentation rate from 405-kyr astronomical calibration.

Secondly: l. 382: "Mudstones with low calcium carbonate content were excluded." -> this is potentially problematic, since the interpreted eccentricity-scale fluctuations in GR signal are reflecting alternations in clay minerals. See the controversy between Mitchell et al. 2008 and Lanci et al. 2010 about the orbital phase relationship of Cretaceous deep marine anoxia (whether they occurred during eccentricity maxima or minima). It turned out that Lanci et al. had arrived at an opposite phase relationship the one proposed by Mitchell et al., 2008 because they had excluded the black cherts from the $CaCO_3$ analysis, which are the main recorder of the astronomical cyclicity (Batenburg et al. 2012 EGU abstract). For the present manuscript, I understand the argument about the sufficient sampling resolution to capture short eccentricity-scale variability in $\delta^{13}C$ (resolution of ~25 kyr), but that does not exclude the possibility of bias arising from systematically not sampling a particular lithology that is in fact are key recorder of the astronomical-induced climate cyclicity. Or am I missing something here?

Response:

Thank you for your suggestions. We sincerely apologize for any confusion caused by the manner in which we communicated the information. The two studied sections represent typical deep-water carbonate successions. No lithological sampling bias was introduced during the collection of rock samples. Only two out of 1782 analyzed samples from two sections exhibited insufficient calcium carbonate content, rendering them unsuitable for inorganic carbon isotope analysis. In the revised manuscript we now include the following statement: "Rock samples were consistently collected at ~10-cm intervals along the horizons where GR measurements were conducted. No lithological bias occurred during the field sampling process."

Minor points:

l. 93-94 "which is further substantiated by results derived from the ZB23-N64 astronomical solution (Zeebe and Lantink 2024b)" -> the fact that a disruption of the ~405 kyr eccentricity cycle does not occur in the 64 solutions of ZB23 for the late Carboniferous time interval is not an argument that it could not have happened. See p. 5 of Zeebe and Lantink 2024a: the estimated timescale for a possible σ12-resonance occurrence is of order 10^8–10^9 yr. Because so far only 64 solutions have been tested, the youngest possible age of a σ12-resonance interval that could be detectable in the geologic record is yet unknown. So just say that it did not occur in the 64 solutions that currently are available. Also: I would refer to the solutions as "ZB23" not "ZB23-N64".

Response: Thank you for your comments, which improve our understanding about the new ZB23 astronomical solution. We have changed this text to: "Although the stability of the 405-kyr eccentricity cycle in deep time remains a topic of ongoing debate[29,30], geochronological evidence supports its relatively stable period during much of the Carboniferous[31-33], which is further substantiated by results derived from 64 solutions in the ZB23 astronomical solution[34] that currently are available (Supplementary Fig. 3)."

l.116-121: The chaotic nature of secular planetary orbital motion within our solar system was shown decades ago based on astronomical (numerical) evidence (Laskar, 1989) and has been independently confirmed by geological evidence (Ma et al., 2017; Zeebe and Lourens, 2019; Olsen et al., 2019). Hence the phrase "were proposed to be chaotic" is not appropriate as it suggests this is still a topic of debate. In addition, the phrase "It likely supports the non-chaotic behavior of the solar system" is somewhat misleading, because you strictly can't speak of chaotic vs. non-chaotic behavior if the system is inherently chaotic/ not precisely predictable. So I would rephrase (and condense) this part by simply mentioning that the results of amplitude modulation analysis suggest that the periods of the very long eccentricity and obliquity amplitude modulation cycles were similar to those of the more recent past.

Response: We have now chosen not to include discussion on the chaotic or non-chaotic behavior of the solar system. Based on your suggestion, we condensed the text as follows: "Results show main periods of ~1.17 Myr and ~2.36 Myr for the long-period obliquity and eccentricity amplitude modulation cycles, respectively (Supplementary Fig. 5c, d). The periods of million-year-scale astronomical cycles during ~337–300 Ma are indistinguishable from those obtained by the astronomical solution (e.g., ZB18a) for the past 36 Myr[37] (Supplementary Fig. 6)."

l .115: "long-term obliquity" -> long-period obliquity amplitude modulation cycle

Response: Those in manuscript were all modified.

l. 112-113 "Modulation analysis." -> Amplitude modulation analysis

Response: Modified.

l. 426 "ZB23-N64 solution" -> "ZB23"

Response: Changed.

Fig. 2ace: Power spectra are way too small; I can't distinguish areas of increased spectral power from areas of lower spectral power (idem for Supplementary Fig. 2)

Response: We have expanded Fig. 2 and Supplementary Fig. 2 both horizontally and vertically to facilitate a clearer observation of the increased spectral power for readers. Please see the new Fig. 2 and Supplementary Figure 3 below.

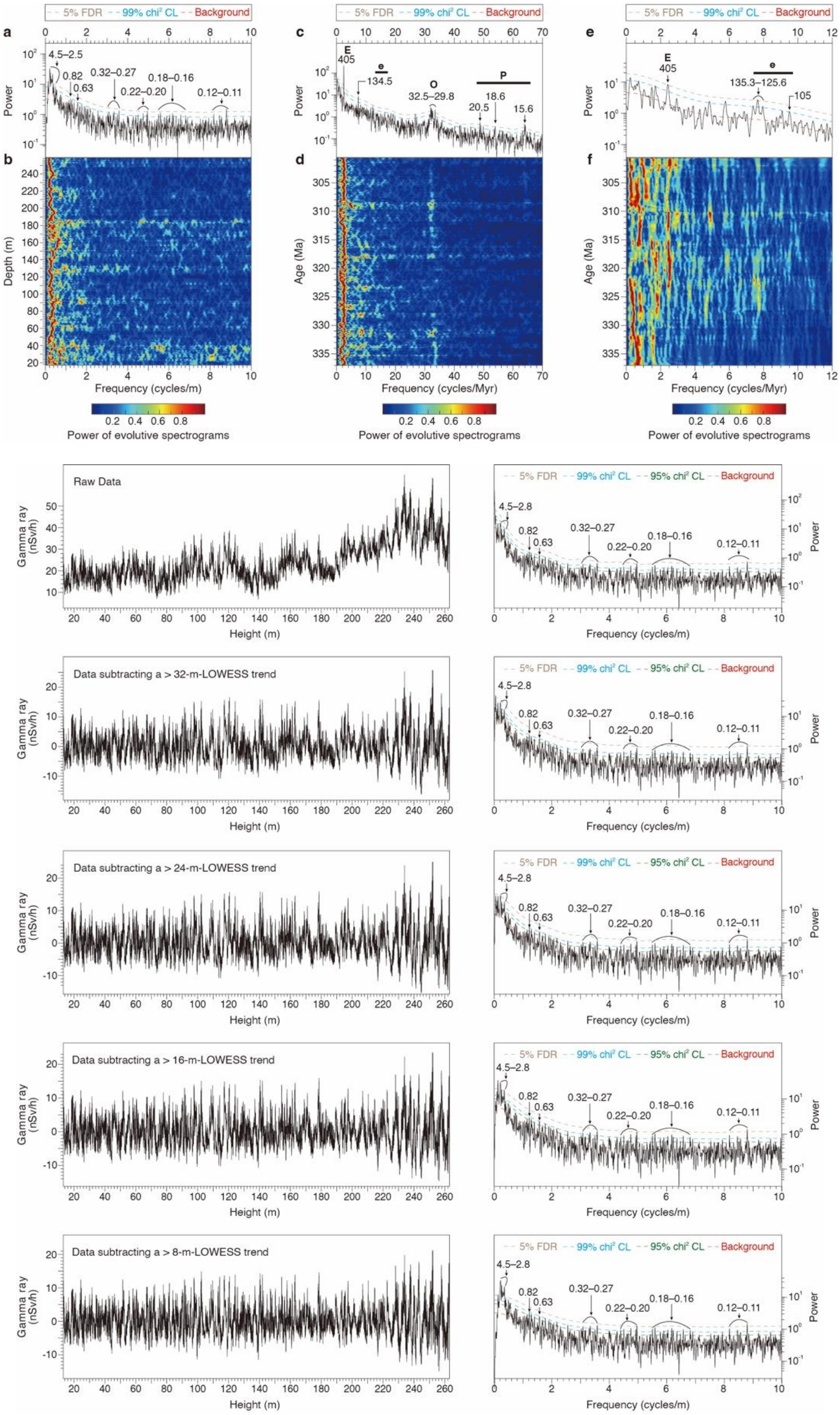

Figure 3 Revised Fig. 2 in the main text and Supplementary Fig. 2 in Supplementary Materials.

I recommend that the authors include Supplementary Fig. 9 in the main article, because it shows the inferred phase relation shift much more clearly than Fig. 3.

Response: After careful consideration, we feel that adding the Supplementary Fig. 9 into the main text might lead to some repetition. To include more information in the main text, we have therefore added a simple schematic diagram in Figure 3j, to enhance the understanding of 'in phase' and 'anti-phase' relations in a more intuitive way for the readers. Please see Figure R4 below.

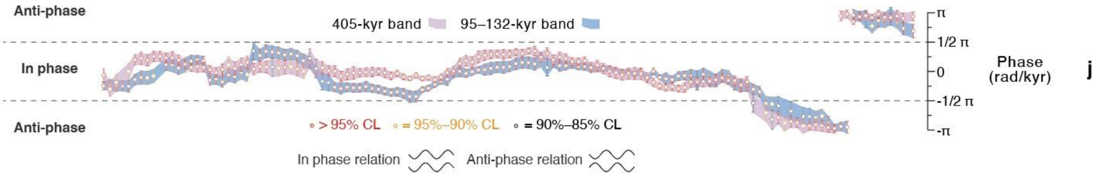

Figure 4 Revised Fig. 3j in the main text.

Supplementary Fig. 4: the translucent gray line (indicating the interpreted average sedimentation rate) is barely visible -> can't you use a thick white line instead?

Response: Modified.

Check for spelling errors – e.g. "ampliutude" an "Costal onlap" in Supplementary Fig. 7.

Response: Corrected.

Response to reviewer #2:

General comments:

The authors have addressed all critiques that I raised in my review, mostly to satisfaction. This must have taken quite some effort, and I commend them for their work. I think that some of the additions to the supplement warrant a mention in the main text, so that readers are aware of the extra analyses that were performed.

Response: Thank you very much for your positive comments on our work. We have thoroughly considered all your suggestions and made modifications accordingly.

Minor points:

The resolution of figure 13/S2 is too low to be able to tell if the spectral analysis on the raw/undetrended record shows the same spectral peaks or not. The spectral peaks associated with long eccentricity at 4.5-2 cycles/m seem to fall below the 99% chi-squared confidence level that the authors use throughout the text, but it's hard to see.

Response: This comment is similar to that of Reviewer #1. As mentioned above, we have expanded Fig. 2 and Supplementary Fig. 2 both horizontally and vertically to facilitate a clearer observation of the increased spectral power for readers. Please see Figure R3 shown above. We have also added a statement on the spectral analysis of the detrended results: "These cycles can be clearly observed in the raw data series and data after applying different ways of detrending (Supplementary Fig. 2)."

The new paragraph that was added starting at L116 is confusing, however, and needs some revision. The solar system has long been known to be chaotic, see e.g., Laskar 1989, Peterson 1993, and many others. Hence, the first sentence needs to be revised. The next sentence needs a qualifier that periods *in the studied time interval* are indistinguishable from those of the past 36 Myr. The concluding sentence is also confusing: the chaos in the solar system is not invalidated, even though the particular feature of the resonance transition that in some solutions resulted in a weak 405 kyr cycle has not manifested in the solutions for this time interval. I would revise to something along the lines of "the long eccentricity cycle appears stable in all currently available numerical solutions for this time interval." Furthermore, I appreciate that the authors performed spectral analysis on the available astronomical solutions for this time interval (ZB23-N64). Their spectral analysis figure shows that in some of the solutions, the spectral power of the long eccentricity cycle is much lower (about a quarter) than the others, indicating variability in the expression of the long eccentricity cycle.

Response: This comment overlaps with a similar comment by Reviewer #1. In this revision we have chosen not to discuss the chaotic/non-chaotic behavior of the solar system. We indicate that the periods of million-year-scale astronomical cycles in the late Carboniferous are the same as those from the past 36 Myr. We condensed the text as follows: "Results show main periods of ~1.17 Myr and ~2.36 Myr for the long-period obliquity and eccentricity amplitude modulation cycles, respectively (Supplementary Fig. 5c, d). The periods of million-year-scale astronomical cycles during ~337–300 Ma are indistinguishable from those obtained by the astronomical solution (e.g., ZB18a astronomical solution) from the past 36 Myr[37] (Supplementary Fig. 6)."

In the response to another reviewer I saw that the authors wrote “Mudstones with low calcium carbonate content were excluded.” This sentence made me worry about mudstones appearing regularly, potentially excluding a part of the cycle. It is essential to explain where these mudstones that were excluded occurred in the record and how frequent they were to make sure that the cyclostratigraphic interpretation is not based on a partial signal.

Response: We have addressed this in a response to a similar comment by Reviewer #1. The two studied sections are deep-water carbonate successions. Only two samples had insufficient calcium carbonate content, rendering them unsuitable for inorganic carbon isotope analysis. To avoid potential misunderstandings, we revised the text as follows: “Rock samples were consistently collected at ~10-cm intervals along the horizons where GR measurements were conducted. No lithological bias occurred during the field sampling process.”.

L416: These analyses were conducted in the Acycle72. -> remove 'the' or add 'software'. Similar for L458--460. Software names are singular.

Response: Modified.

References referred to in responses and not assigned #s (refs in main text).

Montañez, I. P. A. Late Paleozoic climate window of opportunity. *Proc. Natl. Acad. Sci. USA* **13**, 2334–2336 (2016).

Eros, J. M. et al. Sequence stratigraphy and onlap history of the Donets Basin, Ukraine: insight into Carboniferous icehouse dynamics. *Palaeogeogr. Palaeoclimatol. Palaeoecol.* **313**, 1–25 (2012).

Revision notes:

Responses to reviewers:

We appreciate the reviewers' comments, leading to significant improvements of our manuscript. Below the reviewers' comments, and we provide our responses to reviewer comments in blue text.

Response to reviewer #1:

General comments:

I have no further major comments – congratulations with this thought-provoking paper. The suggested similarities between secular climatic trends of the late Paleozoic versus late Cenozoic are intriguing. One recommendation for any follow-up study (?) could be to look in much higher resolution (i.e., resolving precession and obliquity-scale signals) at the key interval where the hypothesized transition in phase relation in the eccentricity band took place. Also, I would still be in favor of adding a zoom-in to Fig. 3 (or something) that shows the phase relation pattern in the raw data (such as in Fig. 1 of Turner, 2014)).

Response: Thank you very much for your positive comments. In response to your suggestion, we will examine precession and obliquity-scale signals where the shift in phase relation in the eccentricity band took place. Additionally, we have conducted a similar analysis on the latter half of the LPIA, and some interesting results have already shown.

We have added a figure 3k to show phase relation pattern in the raw datasets, which allows readers to observe phase changes more clearly. Please refer to the newly revised Fig. 3 below.

Minor points:

l. 113: (*dark* blue arrow in Fig. 1e)

Response: Have added.

l. 206: "ices"?

Response: Have deleted.

l. 258: "We also discovered" -> "We also note/observe…"

Response: Changed into "We also observe".

l. 296-299: This sentence is a bit confusing to me. Do you mean that astronomical forcing played (only) a "passive" role i.e. by modulating/pacing climate change, or actively caused climate change by pushing the system beyond a critical threshold?

Response: What we are referring to is the latter. We changed into "and then orbital forcing may have caused these transient climatic events (thereby determining their timing), <u>by pushing the system beyond the critical thresholds</u>."

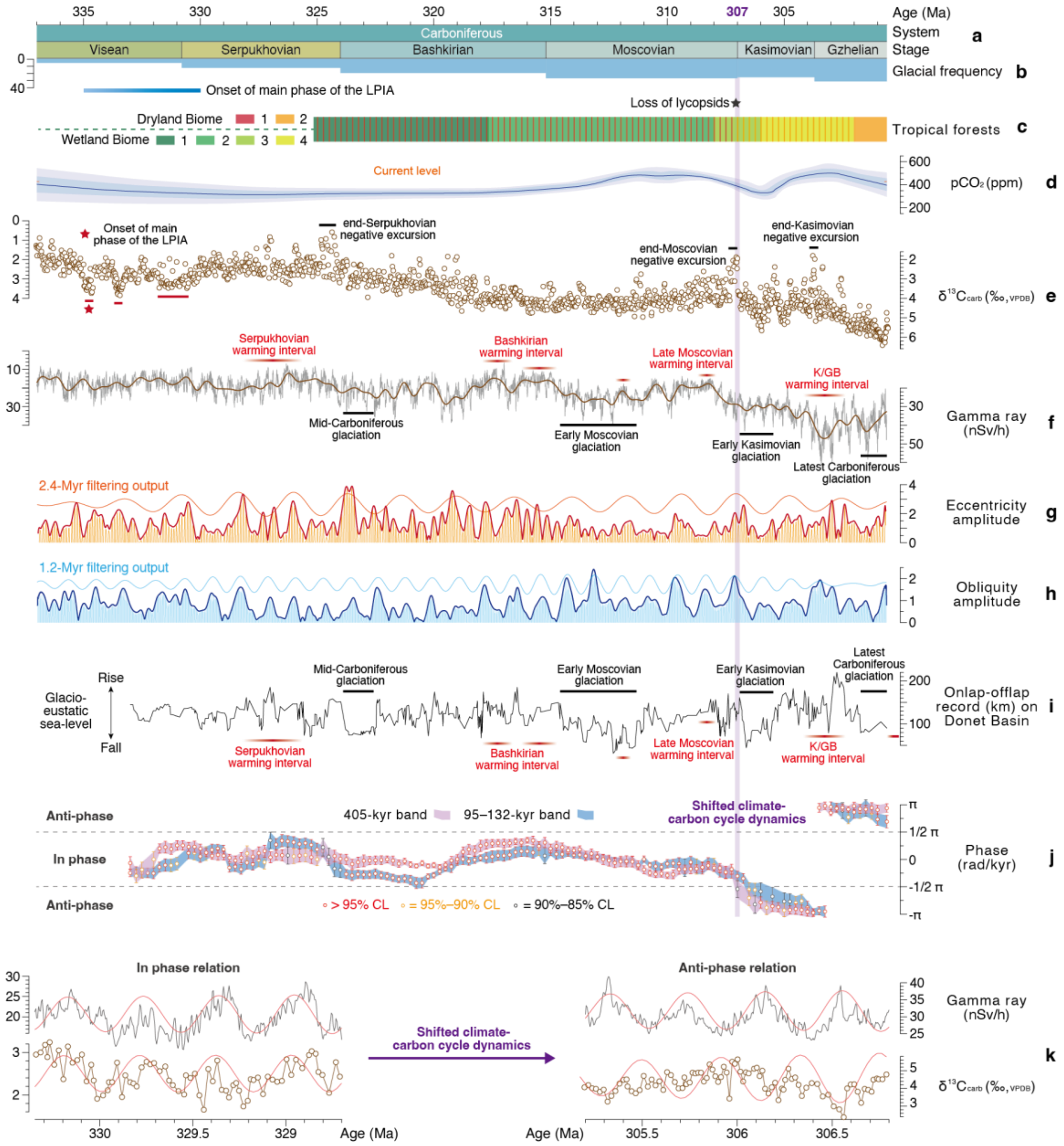

The revised Figure 3

Response to reviewer #2:

General comments:

The revised manuscript addresses most of the remaining critiques.

However, now that we have higher-resolution figures of the spectra, I cannot agree with this newly added sentence on line 90 and 91: "These cycles can be clearly observed in the raw data series and data after applying different ways of detrending (Supplementary Fig. 2)."

The revised fig S2 shows that ONLY the peak near 8.8 cycles/m as well as the lowest-frequency peak near 0.1 cycles/m cross the 5% FDR. The remainder of the peaks in the raw data do not cross the 5% FDR level. The indicated bands do cross the 99% chi-square CL.

Further down in the methods, you state: "A confidence level of ≥ 99% was utilized to validate the existence of significant stratigraphic cycles, minimizing Type II (false negative) errors70. Astronomical calibration can partially reduce disruptions caused by variation in sedimentation rate. To avoid erroneously accepting a null hypothesis indicating no astronomical forcing, a false discovery rate (FDR) threshold of ≤ 5% was established to detect robust signals of astronomical cycles after astronomical calibration, avoiding Type I (false positive) errors69.

So which is it, are you applying a 99% chi-square CL or a 5% FDR?

If the methods would say you choose to use the 99% level in favor of the 5% FDR and articulate why, it would be consistent. But you cannot say you apply both criteria and then not meet those of one of them.

Sorry for being pedantic about the statistics, but this subject has been a hot topic for debate in our field and having a paper published in nature with such confusing wording/inconsistency would only worsen the situation.

Response: We are grateful that you have noticed this issue, and we agree that it is necessary to explain it more clearly. Based on your suggestion, we kept the 99% chi-square CL and removed the 5% FDR in all the spectral analyses (e.g., Fig. 2, S2, S10) in the updated manuscript. We have changed the explanation in the 'Method' section, which was updated to "In cyclostratigraphic studies, confidence levels of >99.9% may effectively minimize the risk of Type I (false positive) errors; however, such excessively stringent confidence levels may increase the likelihood of Type II (false negative) errors[70]. In this study, the confidence levels of 99–99.9% were employed to validate the presence of significant cycles. These levels could balance the probability of incorrectly rejecting and incorrectly accepting a null hypothesis of no astronomical forcing[70]."

Review of "Synchronizing climate-carbon cycle heartbeats in the Phanerozoic vegetated icehouses"

Ilja J. Kocken

October 16, 2024

1 Review

This study presents an impressive record of novel bulk carbonate δ18O measurements, with a new astronomical time scale based on gamma-ray data. The authors argue that the Late Paleozoic Ice Age shows interesting parallels to our current glacial world, and that this record can be used to test this hypothesis. I think the work is significant and warrants publication.

However, there are some major issues with the astronomical solutions and data analysis that should be addressed, requiring major revisions.

2 major points

2.1 Astronomical Solutions

It is problematic to make a comparison between astronomical forcing at ~300.6–337.0 Ma to astronomical forcing for the past 35 Myr without mentioning this much more explicitly than just "in the Cenozoic Era" (lines 95 and 96). The inherent chaos in the solar system makes it impossible to identify a single astronomical solution for this time interval based on physical models alone. This means that it is highly unlikely that the astronomical forcing at around 300 Ma was similar enough to 0–34 Ma to make a direct comparison.

It is surprising to see the authors choose to use astronomical solution La10d because up to the Cretaceous–Paleogene boundary, solutions ZB18a, ZB20x, and to a lesser extent La10a, La10b, and La10c are most compatible with data (Zeebe & Lourens, 2022, EPSL).

The La10x orbital solutions do not include the obliquity and climatic precession components, but only the eccentricity. How did the authors get these? (i.e. L93: "long-term obliquity" and L641: "Raw obliquity signal from La2010d nominal solution24").

Assuming that Acycle was used, it would be based on the methodology presented in Wu et al., (2013), who are not cited here. Furthermore, this approach is problematic because it applies a Fortran script from Laskar et al. (1993) that assumes that input is given the ecliptic reference frame to the La10x solutions, which are given in the invariant reference frame, leading to incorrect results for the obliquity.

The authors also use the La04 solution for determining the target periods for the eCOCO routine. The La04 solution eccentricity is already invalid for time periods prior to ~40 Myr (e.g., Laskar 2011). The obliquity and climatic precession estimates have even more uncertainty and should therefore not be used for a time period of ~300 Ma.

There is a recent ensemble of 64 astronomical solutions available that cover this time period (ZB23-N64, between 0 and -3.5 Gyr). These solutions do include the climatic precession and obliquity (Zeebe and Lantink, 2024, Astronomical Journal, `https://doi.org/10.3847/1538-3881/ad32cf`. Solutions available on `https://www.soest.hawaii.edu/oceanography/faculty/zeebe_files/Astro.html`).

Furthermore, it was recently demonstrated that the long eccentricity cycle (the "405 kyr cycle") is not stable over long time periods as previously thought (Zeebe & Lantink 2024, Astronomical Journal). In the ensemble of simulations, most LEC cycles appear stable over the past 300 Myr, but the period starts to vary and some solutions diverge significantly after several hundred million years. This needs to be addressed, because the authors rely on this assumption to anchor their 4.5–2.5 m cycles to a regular 405 kyr cycle (perhaps it is also relevant to SI L17: "However, the uppermost Serpukhovian and Bashkirian cyclothems70 fail to conform to the 405-kyr pattern18.").

2.2 Spectral analysis

The authors make use of an MTM spectrum and plot a background estimation using an AR-1 model as well as the 95% significance level. They refer to the spectral peaks that cross this 95% CL as "significant spectral peaks", implying statistical null hypothesis significance testing. Previous studies (Weedon 2022, Earth-Science Reviews `https://doi.org/10.1016/j.earscirev.2022.104261`; Smith 2023 P3 `https://doi.org/10.1016/j.`

palaeo.2023.111744) have indicated problems with this application of spectral peak identification, and it appears from the extended data figure 2 that the authors are aware of this because they also present their analysis using an alternative SWA method with 5% false discovery rates.

However, they do not comment on why they present this alternative analysis or which they prefer, and the main manuscript figure still only uses MTM and refers to spectral peaks that stick out above the offset spectral background estimation as "significant". I realize that this debate is not settled yet and there are as yet no recommended "best practices" that everyone agrees on in the field of cyclostratigraphy, but I believe some mention of this is warranted.

L75: is this 8 m detrending also done prior to spectral analysis?

The effects of first removing the 8-m trend on the spectral analysis are not discussed. See attached "Analysis" section for an illustration of how detrending the data prior to spectral analysis hardly affects the spectrum, but shifts the spectral background estimation of the AR-1 process down a lot, leading to more significant peaks.

Using eTimeOpt requires inputting frequencies for the long and short eccentricity bands, but there are very few constraints on the periods of these cycles for this time interval. From L512 it is unclear what these periods are based on.

Then, for the description of the eCOCO routine at L535 the authors describe that they arrive at these periods by extrapolating from the La04 solution's 249 Ma. As described above, the La04 solution eccentricity is already invalid for time periods prior to ~40 Myr already (Laskar 2011). The obliquity and climatic precession estimates have even more uncertainty and should therefore not be used for a time period of ~300 Ma.

Extended data fig 2: "FDR at 5% is equivalent to the conventional 95% CL"

This is factually incorrect. The false discovery rate is:

$$\text{FDR} = \frac{N\text{reject null is true}}{N\text{reject null is true} + N\text{reject null is false}} \tag{1}$$

It is way more strict than the 95% CL. According to Weedon (2018, Geological Magazine, https://doi.org/10.1017/S0016756818000808, Fig. 3) it corresponds to the 99.9899% CL .

In this case I recommend elaborating more on what the FDR is or removing this sentence.

L618: Extended data fig. 3: estimated sedrate of 0.7 cm/kyr, but tested sedimentation rate range was limited to 0 to 2 cm. Have the authors tried

this analysis with a larger range of sedimentation rates?

3 minor points

L39: Why do you refrain from using the kyr and Myr abbreviations commonly used for durations? i.e. 10^4–10^5-yr → 10 kyr to 100 kyr etc.

L70: The carbonates from … **were** deposited in **a** deep-marine environment.

L78: "stable component throughout the geological history" needs to be changed in light of major comments.

L95: "These periods are indistinguishable to those obtained for the astronomical solution (~1.2 Myr and ~2.4 Myr) in the Cenozoic Era19,24 (Extended Data Fig. 5)." There are many different astronomical solutions. Even in the two citations given, there are already 5 (La04, La10a-d).

L597: Photos in Extended data fig 1. seem washed out (backlit, from the glare in the top right of the middle bottom photo?) and could do with some contrast enhancement. It is hard to see the patterns indicated with the lines.

4 analysis

Reproduce spectral analysis

4.1 load libraries

```
library(tidyverse)
library(readxl)
library(astrochron)

# some wrappers for spectral analysis, available on
↪  https://github.com/japhir/CretaceousConstraints
##
↪  remotes::install_github("https://github.com/japhir/CretaceousConstraints")
library(CretaceousConstraints)
```

4.2 load data from supplement

```
si_files <-
↪  list.files("~/SurfDrive/Postdoc1/review/2024-09-26_Wu_carboncycle_heartbeats/539400_0_other_1_sk59w
                          "xlsx",
                          full.names = TRUE)
```

```r
GR_height <- read_excel(si_files[1], range = "A2:B5003", sheet =
↪  "Figure1c-f")
GR_height <- GR_height |> rename(height = `Height (m)`, gamma_ray = `Gamma
↪  ray (nSv/h)`)

GR_height_det <- read_excel(si_files[2], range = "A2:B5003") # detrended
GR_height_det <- GR_height_det |> rename(height = `Height (m)`, gr_det =
↪  `Detrended GR`)
```

4.3 perform spectral analysis

```r
GR_height_spec <- spectral_analysis(GR_height, x = height, y = gamma_ray)
GR_height_spec_det <- spectral_analysis(GR_height_det, x = height, y =
↪  gr_det)
```

4.4 plot spectral analysis

This shows how the spectral background estimation is shifted by detrending.

```r
GR_height_spec |>
  mutate(method = "raw") |>
  bind_rows(GR_height_spec_det |> mutate(method = "detrended")) |>
  plot_spectrum(group = "method",
              periods = c(4.5, 2.5,
                          0.28, 0.20,
                          0.18, 0.15,
                          0.13, 0.10)) +
  theme(legend.position = "inside", legend.position.inside = c(.8, .8))
  ## geom_vline(xintercept = c(0.43 + c(-1, 1) * 0.28), colour = "red")
```

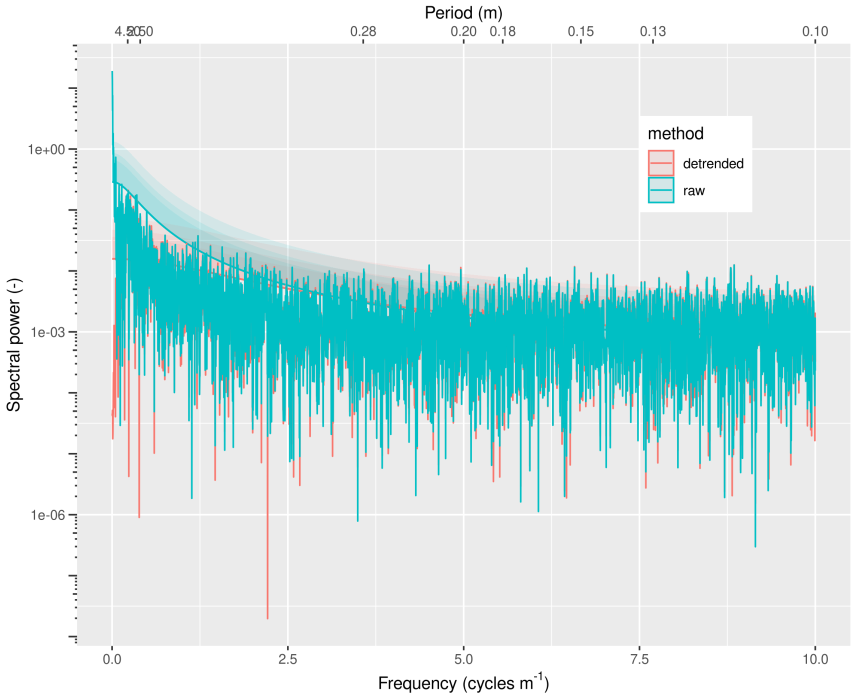

4.5 bandpass filtering

```
GR_bp <- bandpass_filter(GR_height,
                         frequencies = tibble(flow = 0.43 - 0.28, # in
                         ↪  cycles/m
                                              fhigh = 0.43 + 0.28,
                                              target = "LEC"),
                         x = height, y = gamma_ray,
                         window = 1)

GR_bp_det <- bandpass_filter(GR_height_det,
                             frequencies = tibble(flow = 0.43 - 0.28, # in
                             ↪  cycles/m
                                                  fhigh = 0.43 + 0.28,
                                                  target = "LEC"),
                             x = height, y = gr_det,
                             window = 1)
```

4.6 plot bandpass filters

For bandpass filtering, detrending first does not matter.

```
GR_bp |>
  mutate(method = "raw") |>
  bind_rows(GR_bp_det |> mutate(method = "detrend")) |>
  ggplot(aes(x = height, y = gamma_ray, colour = method)) +
  geom_line(alpha = .1) +
  geom_line(aes(y = gr_det), alpha = .1) +
  geom_line(aes(y = filter))##  +
  # to verify: this is nearly identical
  ## geom_line(aes(y = filter - mean(filter)),
  ##           colour = "gold", linetype = "dashed",
  ##           data = GR_bp)
```

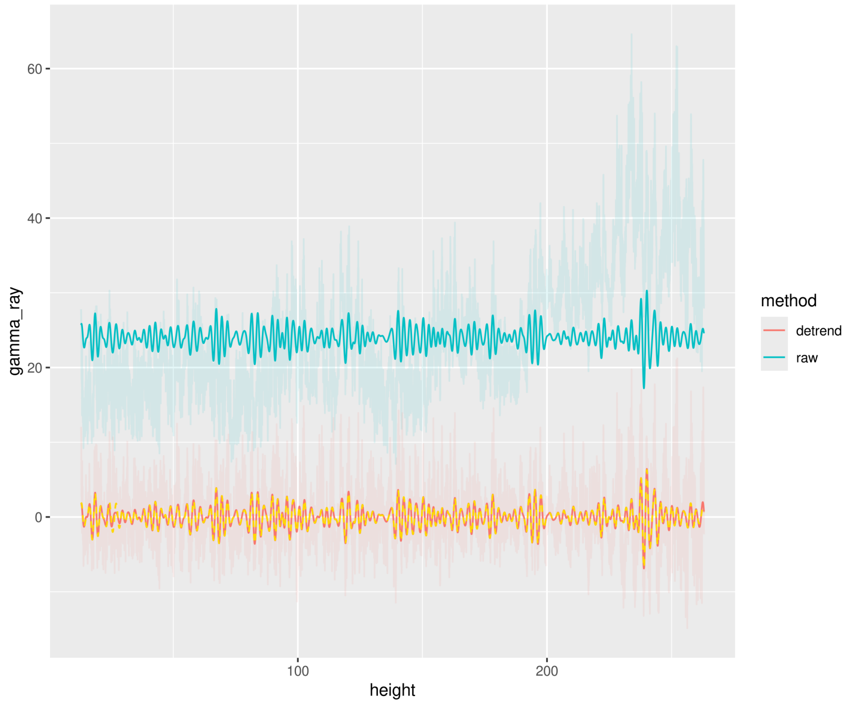

4.7 spectral analysis in time domain

```
GR <- read_excel(si_files[2], range = "D2:E5003")
GR <- GR |> mutate(age = `Age (Ma)` * 1e3)
GR_spec <- spectral_analysis(GR, x = age, y = `Detrended GR`)

plot_spectrum(GR_spec, domain = "time")
```

4.8 eTimeOpt

```r
eto <- GR |>
  linterp(genplot = FALSE) |>
  eTimeOpt(
    sedmin = 0, sedmax = 2, numsed = 200,
    flow = 1 / 160, fhigh = 1 / 80,
    # where do these periods come from???
    targetE = c(405, 132.1, 124.1, 98.7, 95.3))
```

```
----- PERFORMING EVOLUTIVE TimeOpt ANALYSIS -----

----- APPLYING PIECEWISE-LINEAR INTERPOLATION TO STRATIGRAPHIC SERIES -----

 * Sorting data into increasing height/depth/time, removing empty entries

 * Number of samples= 5001
 * Determining median sampling interval for series
 * Will interpolate to median sampling interval of 0.00675
 * New number of samples= 5400
 * Number of data points in stratigraphic series: 5400
 * Stratigraphic series length (space or time): 36.44325
 * Sampling interval (space or time): 0.00675

 * Number of data points per window: 101
 * Moving window size (space or time): 0.675
 * Window step points: 10
 * Window step (space or time): 0.0675
 * Number of windows: 530

 * PLEASE WAIT: Performing Optimizations

0%       25%       50%       75%       100%
===========================================
```
